# An open source knowledge graph ecosystem for the life sciences

**DOI:** 10.1038/s41597-024-03171-w

**Published:** 2024-04-11

**Authors:** Tiffany J. Callahan, Ignacio J. Tripodi, Adrianne L. Stefanski, Luca Cappelletti, Sanya B. Taneja, Jordan M. Wyrwa, Elena Casiraghi, Nicolas A. Matentzoglu, Justin Reese, Jonathan C. Silverstein, Charles Tapley Hoyt, Richard D. Boyce, Scott A. Malec, Deepak R. Unni, Marcin P. Joachimiak, Peter N. Robinson, Christopher J. Mungall, Emanuele Cavalleri, Tommaso Fontana, Giorgio Valentini, Marco Mesiti, Lucas A. Gillenwater, Brook Santangelo, Nicole A. Vasilevsky, Robert Hoehndorf, Tellen D. Bennett, Patrick B. Ryan, George Hripcsak, Michael G. Kahn, Michael Bada, William A. Baumgartner, Lawrence E. Hunter

**Affiliations:** 1 Computational Bioscience Program, University of Colorado Anschutz Medical Campus, Aurora, CO 80045 USA; 2 Department of Biomedical Informatics, Columbia University Irving Medical Center, New York, NY 10032 USA; 3 Computer Science Department, Interdisciplinary Quantitative Biology, University of Colorado Boulder, Boulder, CO 80301 USA; 4 AnacletoLab, Dipartimento di Informatica, Universit`a degli Studi di Milano, Via Celoria 18, 20133 Milan, Italy; 5 Intelligent Systems Program, University of Pittsburgh, Pittsburgh, PA 15260 USA; 6 Department of Physical Medicine and Rehabilitation, School of Medicine, University of Colorado Anschutz Medical Campus, Aurora, CO 80045 USA; 7 Division of Environmental Genomics and Systems Biology, Lawrence Berkeley National Laboratory, Berkeley, CA 94720 USA; 8 Semanticly, Athens, Greece; 9 Department of Biomedical Informatics, University of Pittsburgh School of Medicine, Pittsburgh, PA 15206 USA; 10 Laboratory of Systems Pharmacology, Harvard Medical School, Boston, MA 02115 USA; 11 Division of Translational Informatics, University of New Mexico School of Medicine, Albuquerque, NM 87131 USA; 12 SIB Swiss Institute of Bioinformatics, Basel, Switzerland; 13 Berlin Institute of Health at Charité-Universitatsmedizin, 10117 Berlin, Germany; 14 ELLIS, European Laboratory for Learning and Intelligent Systems, Milan Unit, Italy; 15 Department of Biomedical Informatics, University of Colorado School of Medicine, Aurora, CO 80045 USA; 16 Data Collaboration Center, Critical Path Institute, 1840 E River Rd. Suite 100, Tucson, AZ 85718 USA; 17 Computer, Electrical and Mathematical Sciences & Engineering Division, Computational Bioscience Research Center, King Abdullah University of Science and Technology, Thuwal, 23955-6900 Kingdom of Saudi Arabia; 18 Department of Pediatrics, University of Colorado School of Medicine, Aurora, CO 80045 USA; 19 Janssen Research and Development, Raritan, NJ 08869 USA; 20 Division of General Internal Medicine, University of Colorado School of Medicine, Aurora, CO 80045 USA

**Keywords:** Software, Software

## Abstract

Translational research requires data at multiple scales of biological organization. Advancements in sequencing and multi-omics technologies have increased the availability of these data, but researchers face significant integration challenges. Knowledge graphs (KGs) are used to model complex phenomena, and methods exist to construct them automatically. However, tackling complex biomedical integration problems requires flexibility in the way knowledge is modeled. Moreover, existing KG construction methods provide robust tooling at the cost of fixed or limited choices among knowledge representation models. PheKnowLator (Phenotype Knowledge Translator) is a semantic ecosystem for automating the FAIR (Findable, Accessible, Interoperable, and Reusable) construction of ontologically grounded KGs with fully customizable knowledge representation. The ecosystem includes KG construction resources (e.g., data preparation APIs), analysis tools (e.g., SPARQL endpoint resources and abstraction algorithms), and benchmarks (e.g., prebuilt KGs). We evaluated the ecosystem by systematically comparing it to existing open-source KG construction methods and by analyzing its computational performance when used to construct 12 different large-scale KGs. With flexible knowledge representation, PheKnowLator enables fully customizable KGs without compromising performance or usability.

## Introduction

The worldwide growth of biomedical data is exponential, with the volume of molecular data alone expected to surpass more than four exabytes by 2025^1^. Translational science requires integrating data and knowledge at multiple scales of biological organization. Rapid advancements in sequencing and multi-omics technologies have made tremendous amounts of diverse data available for secondary use^2–5^. Multimodal data like these capture different views and, when properly combined, help characterize complex systems^6^. Unfortunately, these data are highly distributed and heterogeneous, can be difficult to access due to licensing restrictions, lack interoperability, and often have inconsistent underlying models or representations, which limit most researchers from fully utilizing them^7,8^.

**Fig. 1:**
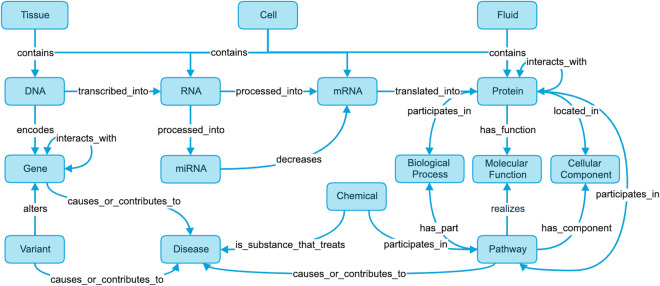
A Knowledge Representation of the Levels of Biological Organization Underlying Human Disease. This knowledge graph provides a representation of our currently accepted knowledge of the Central Dogma expanded to include pathways, variants, pharmaceutical treatments, and diseases^10^. At a high level this knowledge graph represents anatomical entities such as tissues, cells, and bodily fluids containing genomic entities such as DNA, RNA, mRNA, and proteins. DNA encodes genes that are processed into mRNA and translated into proteins, which can interact with each other. Genes can also be altered by variants and may cause disease. Finally, proteins also have molecular functions and participate in pathways and biological processes.

Knowledge graphs (KGs) have frequently been used to systematically model and interrogate the biology underlying complicated systems, organisms, and diseases^9^. For example, Fig. 1 provides a high-level overview of the main biomedical concepts needed to model our currently accepted knowledge of the Central Dogma^10^ and has been expanded to include pathways, variants, pharmaceutical treatments, and diseases. In the life sciences, KGs are usually constructed from a wide range of data sources such as Linked Open Data (http://www.w3.org/DesignIssues/LinkedData.html), ontologies, the scientific literature, data derived from electronic health records, and multi-omics experiments^8,11^. In the biomedical context, nodes usually represent different kinds of biological entities such as genes, proteins or diseases, and edges (or triples) are used to specify different types of relationships that can exist between a pair of nodes (e.g., “interaction”, “substance that treats”). Multiple definitions of KGs have been proposed in the literature, all sharing the assumption that KGs are more than simple large-scale graphs^12–14^. Existing definitions are best summarized by Ehrlinger’s and Wöb’s^12^ definition: “A knowledge graph acquires and integrates information into an ontology and applies a reasoner to derive new knowledge“^12^. We provide an alternative definition and consider a KG to be a graph-based data structure representing a variety of heterogeneous entities with multiple types of relationships between them that serves as an abstract framework capable of inferring new knowledge (as well as revealing and resolving discrepancies or contradictions) to address a variety of applications and use cases.

KG construction is not a simple process, requiring significant data preprocessing or wrangling before edge lists can be assembled. Fortunately, several methods have been developed to tackle the primary challenges faced when constructing a KG, including: the integration or harmonization of disparate resources (e.g., SPOKE^15^, RTX-KG2^16^, Petagraph^17^, Bio2RDF^18^, and Hetionet^19^), processing and formatting of structured data and KGs (e.g., Dipper [https://github.com/monarch-initiative/dipper] and the Knowledge Graph Exchange [KGX; https://github.com/biolink/kgx]), enhancement or extraction of relationships (e.g., Biomedical Knowledge Discovery Engine [BioKDE]^20^ and KG-COVID-19^21^) and evidence (e.g., PrimeKG^22^) from the literature, and the exchange or sharing of constructed KGs (e.g., Network Data Exchange [NDEx]^23^ and KGX). Recently, several frameworks such as KG-HUB^24^, the Clinical KG (CKG)^25^, RTX-KG2^16^, BioCypher^26^, and the Knowledge Base Of Biomedicine (KaBOB)^7^ which provide all of the aforementioned functionalities, have been developed. While methods have been developed for each of the processes or steps required to construct KGs, robust tools and resources to evaluate constructed KGs are lacking^8^. Traditionally, the evaluation of constructed KGs has been task- or domain-specific and largely limited to case studies^15,16,19,21,22,25,26^. Ideally, constructed KGs would be evaluated in the same manner as other network science (e.g., community detection and link prediction algorithms) and KG or node embedding methods using benchmarks such as Zachary’s Karate Club graph^27^, DBPedia (https://www.dbpedia.org/resources/knowledge-graphs), and OpenBioLink^28^. KG benchmarks could be used to assess the computational performance of KG construction methods as well as to evaluate the implications of different knowledge representations on specific tasks. To the best of our knowledge, no existing benchmarks exist to systematically evaluate knowledge representation.

**Fig. 2:**
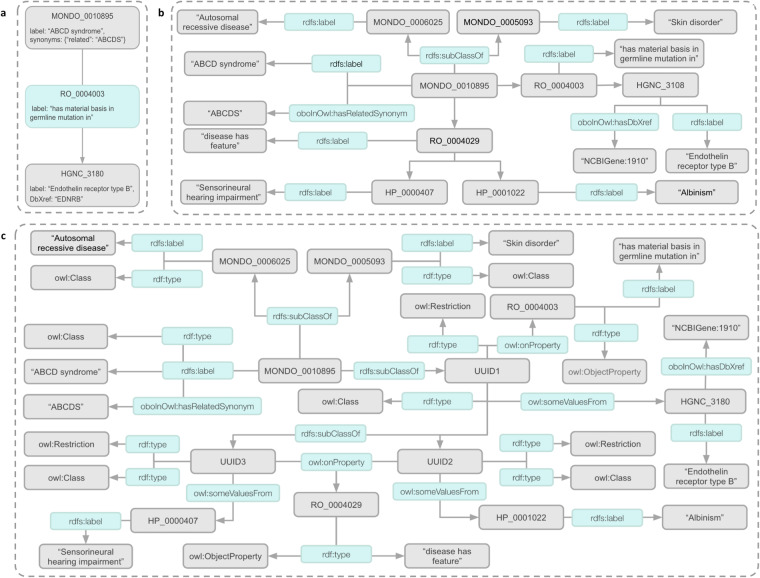
Types of Knowledge Graphs used in the Life Sciences. This figure provides examples of three types of knowledge graphs that are typically used in the Life Sciences. All knowledge graphs are modeling the Mondo concept ABCD syndrome (*MONDO:0010895*). (**a**) illustrates a simple graph-based representation where two nodes are connected by an edge and nodes and edges are assigned attributes in the form of key-value pairs. (**b**) illustrates a hybrid or property graph-based representation where edges are represented as sets of three nodes (each composed of a subject, predicate, and object) called triples, often based on the RDF/RDFS standards. (**c**) illustrates a complex or OWL-graph-based representation where edges are represented as triples and these representations are augmented with additional OWL expressivities such as domain/range or cardinality restrictions. Acronyms: HP (Human Phenotype Ontology); MONDO (Mondo Disease Ontology); OWL (Web Ontology Language); RDF (Resource Description Framework); RDFS (Resource Description Framework Syntax); RO (Relation Ontology).

Tackling complex problems within the life sciences requires flexible knowledge representations. An important limitation of existing KG construction methods is fixed or limited flexibility in the way that knowledge is modeled. Within the biomedical domain, knowledge is typically modeled in one of three ways (Fig. 2), though the nomenclature used to describe these different approaches differs widely in the literature. For simplicity’s sake, we will refer to the three different approaches as simple, hybrid, and complex. The first approach results in a simple graph (Fig. 2a). Simple graphs (Fig. 2a) are the most common type of network used in the literature. Examples of simple graphs include Zachary’s Karate Club graph^27^, Hetionet^19^, and SPOKE^15^. In these graphs, entities are represented as nodes, and edges are used to model relationships between them. These graphs usually lack formal semantics for the edges and nodes. Edges are often semantically overloaded, ignoring the distinction between data (e.g., a protein participating in a process) and metadata (e.g., the source of information about the protein’s participation in that process). Simple graphs are usually straightforward to construct and can be stored as key-value pairs, resulting in small file sizes and using modest amounts of memory. Disadvantages of simple graphs include ad hoc semantics, which decreases interoperability, and a lack of clear specification, making machine inference difficult. The second approach results in a hybrid or property graph (Fig. 2b). Example hybrid graphs include KG-COVID-19^21^, DisGeNET^29^, OpenBioLink^28^, Petagraph^17^, the Monarch KG^30^, and Bio2RDF^18^. Hybrid graphs aim to model entities and their relations using a mix of standard network representations and formal semantics, usually the Resource Description Framework (RDF; https://www.w3.org/RDF) and RDF Schema (RDFS; https://www.w3.org/TR/rdf11-mt). Compared to simple graphs, standards-based hybrid graphs facilitate integration with other resources^31^ and are more amenable to automated inference. They also provide faceted querying as nodes and edges are typed. One cost of hybrid graphs is that they require substantially more storage space than simple graphs. The third approach results in a complex graph, such as KaBOB^7^, often built on the Web Ontology Language (OWL; https://www.w3.org/TR/owl-features) (Fig. 2c). Complex graphs are more expressive, facilitating the generation of new knowledge via deductive inference^32^. By enforcing explicit semantics, OWL provides advantages over RDF/RDFS in the integration of large biomedical data^33^. Complex graphs are fully machine-readable, highly expressive, and, because they are built on Description Logics^32^, can leverage reasoners to verify their logical consistency and do deductive inference. Unlike simple graphs, both hybrid and complex graphs can distinguish between data and metadata as demonstrated in Fig. 2. Unless defining custom relations, hybrid graphs do this by primarily using RDFS and resources like the OBO Format metamodel (https://www.bioontology.org/wiki/OboInOwl:Main_Page), whereas complex graphs formally define these types and their attributes using RDF and OWL. Unfortunately, complex graphs are very large, can be difficult for humans to understand, and have been shown to perform poorly on some inductive inference tasks^34^. To date, none of the existing KG construction methods enable the construction of multiple or alternative versions of the same KG utilizing different underlying knowledge representations, making comparisons, and benchmarking difficult.

**Fig. 3:**
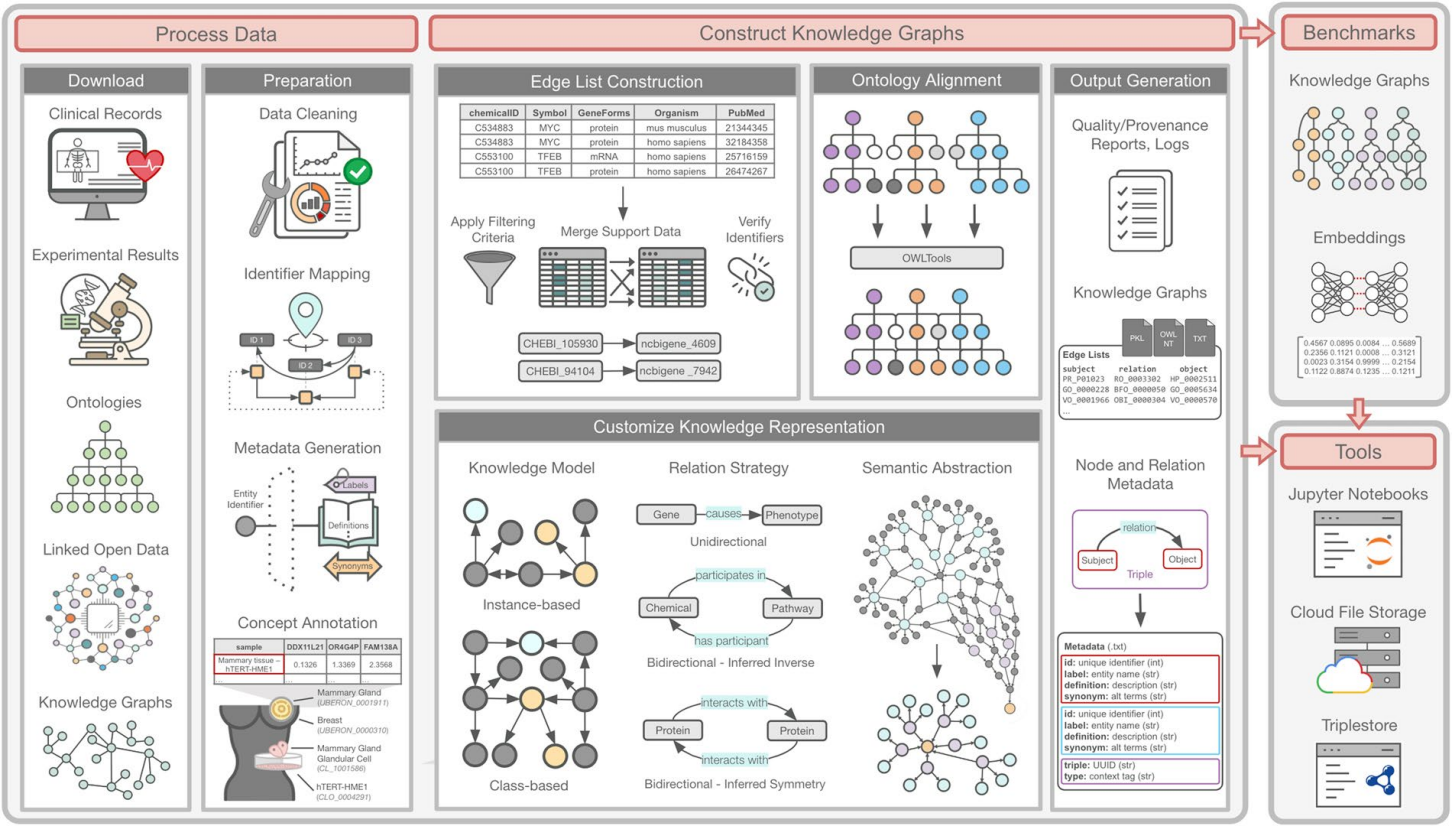
The PheKnowLator Ecosystem. This figure provides an overview of the PheKnowLator ecosystem^106^. The ecosystem consists of three components as indicated by the gray boxes: (1) **Knowledge Graph Construction Resources**, which consist of resources to download and process data and an algorithm to customize the construction of large-scale heterogeneous biomedical knowledge graphs; (2) **Knowledge Graph Benchmarks**, which consist of prebuilt KGs that can be used to systematically assess the effects of different knowledge representations on downstream analyses, workflows, and learning algorithms; and (3) **Knowledge Graph Tools** to use knowledge graphs, cloud-based data storage, APIs, and triplestores. Acronyms: NT (N-Triples file format); OWL (Web Ontology Language); PKL (Python pickle file format); SPARQL (SPARQL Protocol and RDF Query Language).

To address the lack of relevant benchmarks and flexibility in knowledge representation, we developed PheKnowLator (Phenotype Knowledge TransLator, referred to as “PKT” throughout the remainder of this manuscript), a semantic ecosystem for automating the FAIR (Findable, Accessible, Interoperable, and Reusable)^35^ construction of ontologically grounded KGs with fully customizable knowledge representation. The ecosystem consists of three components (Fig. 3): (1) **KG Construction Resources**, a set of tools to download and process heterogeneous data and algorithms to construct custom KGs; (2) **KG Benchmarks**, a collection of prebuilt KGs that can be used to systematically assess the effects of different knowledge representations on downstream analyses, workflows, and learning algorithms; and (3) **KG Tools** to analyze KGs, including Jupyter Notebook-based tutorials, archive-based data storage, application programming interfaces (APIs), and triplestores. We evaluate the PheKnowLator ecosystem by systematically comparing its components with existing open-source KG construction software using a survey developed to assess the functionality, availability, usability, maturity, and reproducibility of KG construction software. We also assess the ecosystem’s computational performance when constructing 12 different types of benchmark KGs designed to provide alternative representations for modeling the molecular mechanisms underlying human disease.

## Results

PheKnowLator is open-source and available through GitHub (https://github.com/callahantiff/PheKnowLator) and PyPI (https://pypi.org/project/pkt-kg). Important manuscript definitions are provided in Supplementary Table 1, acronyms are provided in Supplementary Table 2, and PheKnowLator ecosystem resources are listed in Supplementary Tables 3 and 4.

### Evaluation

The PheKnowLator ecosystem was evaluated in two ways. First, publicly available software to construct biomedical KGs was identified and systematically compared using a survey developed to assess each method’s functionality, availability, usability, maturity, and reproducibility. Second, the computational performance of the ecosystem was assessed when used to construct 12 different types of benchmark KGs designed to provide alternative representations for modeling the molecular mechanisms underlying human disease. The resources used for each task are listed in Supplementary Table 4.

#### Systematic comparison of open-source KG construction software

**Fig. 4:**
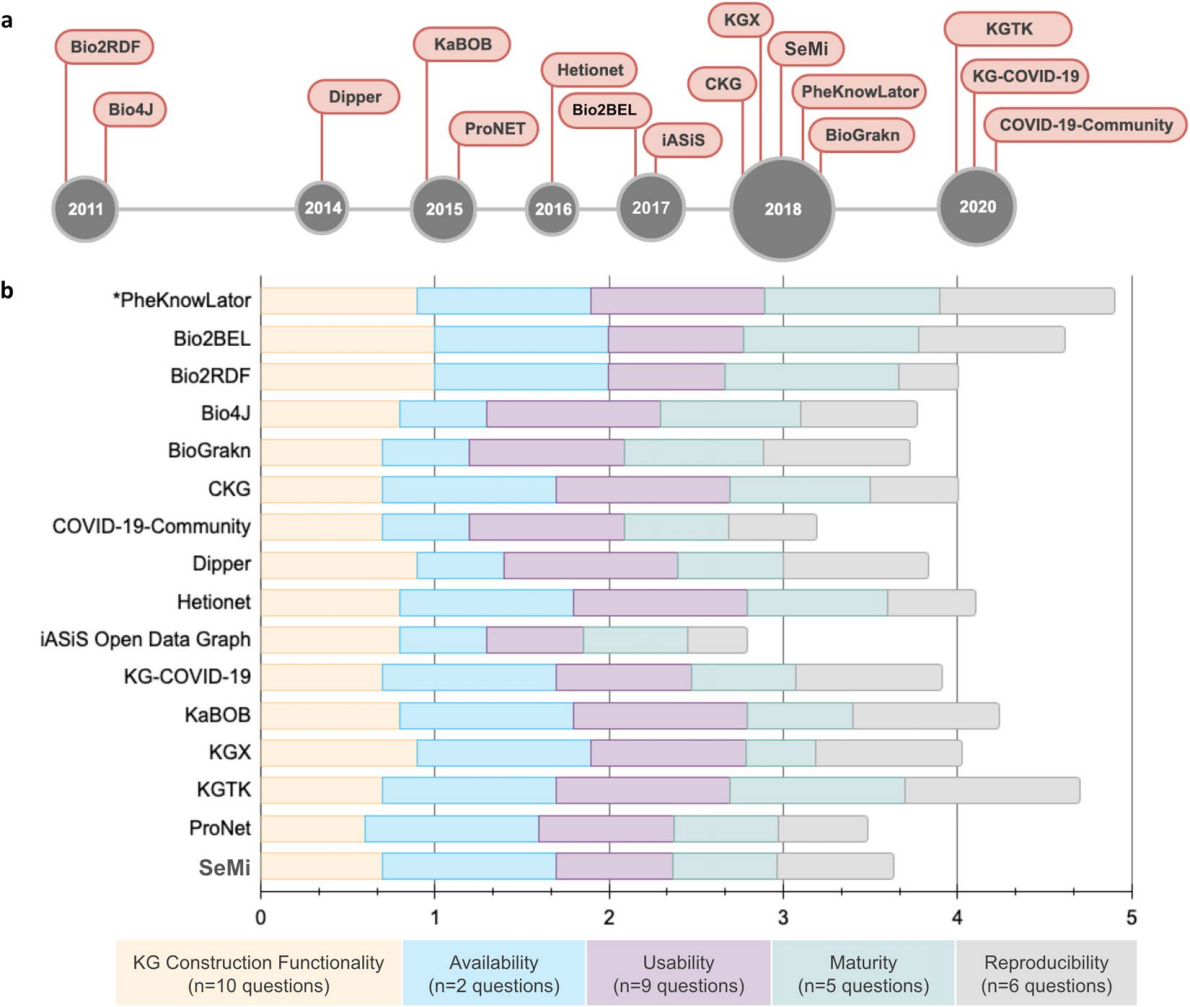
Open-Source Knowledge Graph Construction Methods - Survey Results. This figure presents the open-source knowledge graph construction methods identified on GitHub and the results of the survey assessment. (**a**) The final set of 16 knowledge graph construction methods surveyed according to the year they were first published on GitHub. (**b**) A chart of the methods evaluated in terms of the different survey categories. The survey was scored out of a total score of five points, which was derived as the sum of the ratios of coverage, each out of one point, for the five categories: KG Construction Functionality (10 questions); Availability (two questions); Usability (nine questions); Maturity (five questions); and Reproducibility (six questions). Acronyms: iASiS, Automated Semantic Integration of Disease-Specific Knowledge; KaBOB, Knowledge Base Of Biomedicine; KG, (Knowledge Graph); KGX (Knowledge Graph Exchange); KGTK (Knowledge Graph Toolkit); SeMi (SEmantic Modeling machine).

Open-source biomedical KG construction methods available on GitHub were identified and compared to the PheKnowLator ecosystem. A survey was used to compare the methods for the task of constructing biomedical KGs and consisted of 44 questions designed to assess five criteria: KG construction functionality, maturity, availability, usability, and reproducibility (Supplementary Table 5). Of the 1,905 repositories identified on GitHub, 231 contained course, tutorial, or presentation material (i.e., manuscript reviews and slide decks), 278 were duplicate or cloned repositories, 79 were KG applications or services, 60 were websites or resource lists, and 1,253 were determined to be irrelevant (i.e., mislabeled, not biomedical, or not a KG construction method). This initial list was supplemented with 11 methods identified through a review article^8^ or were recommended by a collaborator. The final list included 15 methods (see Table 1 with additional details provided in Supplementary Table 6): Bio2Bel (ttps://github.com/bio2bel), Bio2RDF (https://github.com/bio2rdf), Bio4J (https://github.com/bio4j/bio4j), BioGrakn (https://github.com/vaticle/biograkn), the Clinical Knowledge Graph (https://github.com/MannLabs/CKG), COVID-19-Community (https://github.com/covid-19-net/covid-19-community), Dipper, Hetionet (https://github.com/hetio/hetionet), IASiS Open Data Graph (https://github.com/tasosnent/Biomedical-Knowledge-Integration), KG-COVID-19 (https://github.com/Knowledge-Graph-Hub/kg-covid-19), KaBOB (https://github.com/UCDenver-ccp/kabob), KGX, the Knowledge Graph Toolkit (https://github.com/usc-isi-i2/kgtk), ProNet (https://github.com/cran/ProNet), and the SEmantic Modeling machIne (https://github.com/giuseppefutia/semi). The methods are visualized by date of GitHub publication in Fig. 4a.

**Table 1:** Open-Source Knowledge Graph Construction Methods.

Method	GitHub Repository
Bio2BEL	https://github.com/bio2bel/
Bio2RDF	https://github.com/bio2rdf
Bio4J	https://github.com/bio4j/bio4j
BioGrakn	https://github.com/graknlabs/biograkn
Clinical Knowledge Graph (CKG)	https://github.com/MannLabs/CKG
COVID-19-Community	https://github.com/covid-19-net/covid-19-community
Dipper	https://github.com/monarch-initiative/dipper
Hetionet	https://github.com/hetio/hetionet
iASiS Open Data Graph	https://github.com/tasosnent/Biomedical-Knowledge-Integration
KG-COVID-19	https://github.com/Knowledge-Graph-Hub/kg-covid-19
Knowledge Base Of Biomedicine (KaBOB)	https://github.com/UCDenver-ccp/kabob/tree/bg-integration
Knowledge Graph Exchange (KGX)	https://github.com/NCATS-Tangerine/kgx
Knowledge Graph Toolkit (KGTK)	https://github.com/usc-isi-i2/kgtk/
ProNet	https://github.com/cran/ProNet
SEmantic Modeling machIne (SeMi)	https://github.com/giuseppefutia/semi

The average coverage score of the five assessment criteria was 3.93 (min = 2.79, max = 4.90). The coverage of each assessment criterion by method is shown in Fig. 4b. Examining the results by assessment criteria revealed interesting patterns. **KG Construction Functionality** (Supplementary Table 7): The majority of the methods (81.3%; n = 13) included functionality to download data, while 56.3% (n = 9) were able to process experimental data and 37.5% (n = 6) were able to process clinical data. **Availability** (Supplementary Table 8): Three-fourths of the methods (75%; n = 12) were written in Python and 43.8% (n = 7) were written in a Java-based language. All the methods but one were licensed with GPL, MIT, or BSD-3. **Usability** (Supplementary Table 9): Sample data were provided by 93.8% (n = 15) of the methods, and 75% (n = 12) provided tutorials via R Markdown or Jupyter Notebook. **Maturity** (Supplementary Table 10): On average, the number of commits per year ranged from 17 to 1,000. Over half of the methods (68.8%, n = 11) had been published, and 43.8% (n = 7) provided collaboration guidelines. **Reproducibility** (Supplementary Table 11): Tools to enable reproducible workflows and aid in installing the method were provided by 75% (n = 12) of the methods. Most often, these tools included Docker containers (n = 6) and Jupyter or R Notebooks (n = 8).

While the PheKnowLator ecosystem was comparable to the other methods on the assessed criteria, we identified three important differentiating factors relative to the other methods: (i) tools to assess the quality of underlying ontologies; (ii) logging and documentation of metadata including the KG construction process, the data downloaded, the processing steps applied to each data source, and the node and edge types each source contributes to; and (iii) customizable knowledge representation making it possible to take advantage of advanced Semantic Web tools like description logic reasoners (which we have successfully applied in the construction of KGs by the PheKnowLator ecosystem). The ability to generate multiple versions of the same KGs enables the ecosystem to provide benchmark KGs, which can be used to evaluate modeling decisions and to study the impact of knowledge representation on downstream learning. PheKnowLator included all the functionalities in the five assessment criteria except for tools to process clinical data, which only 37.5% (n = 6) of the methods provided.

#### Human disease knowledge graph benchmark comparison and construction performance

The PheKnowLator ecosystem enables users to fully customize KG construction by providing the following parameters (described in detail in the *Construct Knowledge Graphs* section of **Component 1: Knowledge Graph Construction Resources** in the Methods): knowledge model (i.e., complex graphs using class- or instance-based knowledge models), relation strategy (i.e., standard directed relations or inverse bidirectional relations), and semantic abstraction (i.e., transformation of complex graphs into hybrid graphs) with or without knowledge model harmonization (i.e., ensuring a hybrid KG is consistent with the class- or instance-based complex graph it was abstracted from). These parameters enable 12 different versions or benchmarks of each KG to be constructed for a given build. Descriptive statistics and computational performance of the PheKnowLator ecosystem was assessed when used to build a large-scale heterogeneous KG designed to represent the molecular mechanisms underlying human disease and its 12 associated KG types or benchmarks (referred throughout the remainder of manuscript as the PKT [PheKnowLator] Human Disease benchmark KGs).

##### Benchmark comparison

**Fig. 5:**
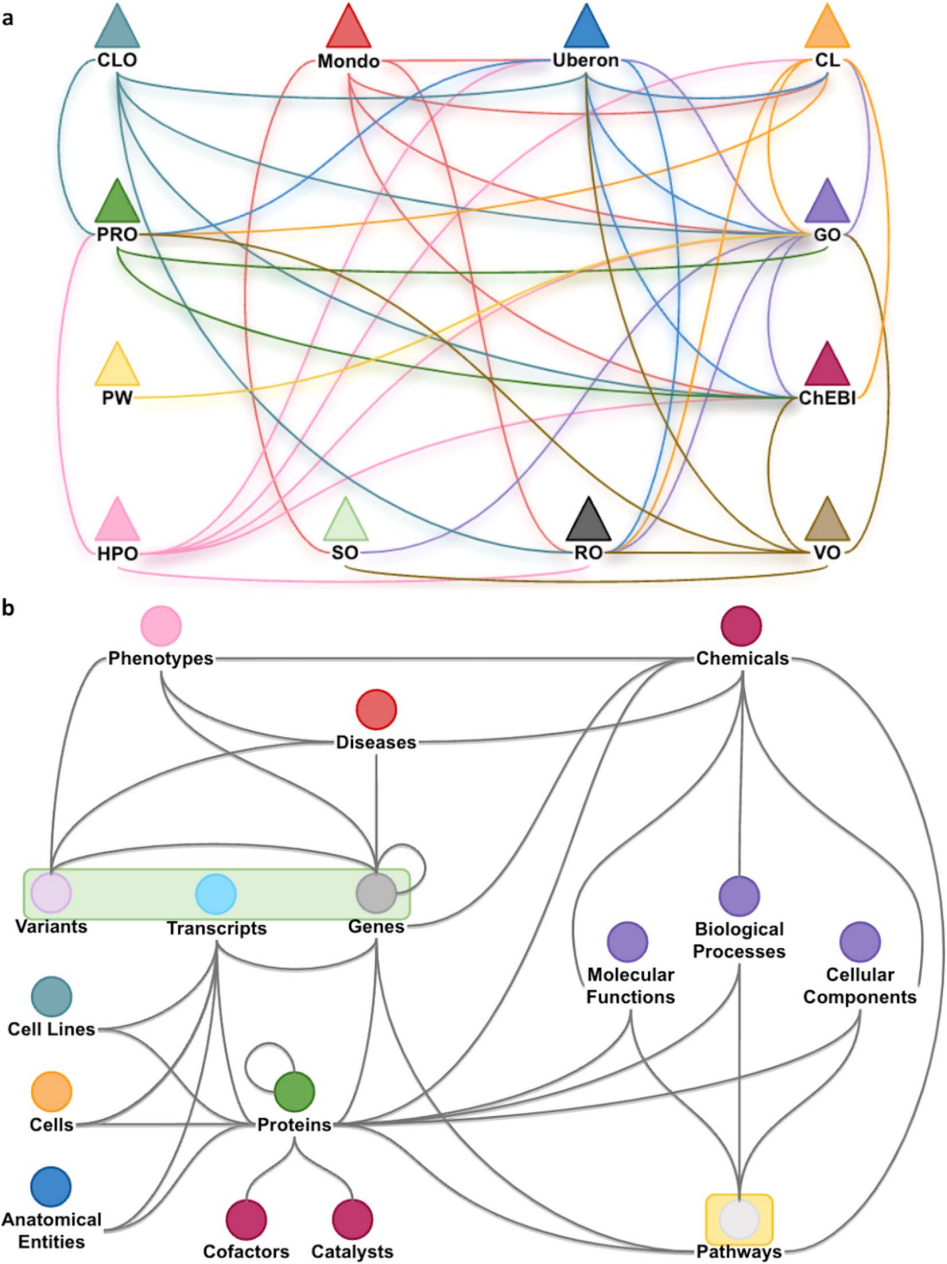
An Overview of the PKT Human Disease Mechanism Knowledge Graph. This figure provides a high-level overview of the primary node and edge types in the PKT Human Disease Mechanism knowledge graph. (**a**) illustrates the relationships between the core set of Open Biological and Biomedical Ontology (OBO) Foundry ontologies when including their imported ontologies (as of August 2022). (**b**) illustrates the edges or triples that are added to the core set of merged ontologies in (**a**). Shared colors between (**a**) and (**b**) represent a single resource. For example, chemicals, cofactors, and catalysts share the same color (maroon) and are part of ChEBI. This is the same for the RO, which is represented in (b) as the black lines between nodes. The green and yellow rectangles indicate data sources that are not from an OBO Foundry ontology and the specific ontology used to integrate them with the core set of ontologies in (**a**). For example, variant, transcript, and gene data are connected to the core ontology set via the SO. Acronyms: CL (Cell ontology); CLO (Cell Line Ontology); ChEBI (Chemical Entities of Biological Interest); GO (Gene Ontology); HPO (Human Phenotype Ontology); Mondo (Mondo Disease Ontology); PRO (Protein Ontology); PW (Pathway Ontology); SO (Sequence Ontology); VO (Vaccine Ontology); Uberon (Uber-Anatomy Ontology).

Under the advice of domain experts (ALS, IJT, LH, and CJM), the PKT Human Disease benchmark KGs were constructed from 12 OBO Foundry ontologies, 31 Linked Open Data sets, and results from two large-scale molecular experiments (all build data are listed and described in Supplementary Table 12). The knowledge representation used for the build is shown in Supplementary Figure 1. A simplified overview of this knowledge representation is provided in Fig. 5, which highlights the connectivity between the 12 OBO Foundry ontologies (Fig. 5a) and their relationship to the primary node types. The 18 primary node types are listed in Table 2 (visualized in Fig. 5b), and 33 primary edge types are shown in Table 3. The primary node and edge types do not include all possible node and edge types made available in the core set of 12 OBO Foundry ontologies, only those that are explicitly modeled in our knowledge representation.

**Table 2:** PKT Human Disease Knowledge Graph Primary Node Types.

Node	Universal Resource Identifier
Anatomical Entities	http://purl.obolibrary.org/obo/UBERON
Biological Processes	http://purl.obolibrary.org/obo/GO
Catalysts	http://purl.obolibrary.org/obo/CHEBI
Cells	http://purl.obolibrary.org/obo/CL
Cell Lines	http://purl.obolibrary.org/obo/CLO
Cellular Components	http://purl.obolibrary.org/obo/GO
Chemicals	http://purl.obolibrary.org/obo/CHEBI
Cofactors	http://purl.obolibrary.org/obo/CHEBI
Diseases	http://purl.obolibrary.org/obo/MONDO
Genes	http://www.ncbi.nlm.nih.gov/gene/
Molecular Functions	http://purl.obolibrary.org/obo/GO
Pathways^a^	http://purl.obolibrary.org/obo/PW https://reactome.org/content/detail/R-HSA-
Phenotypes	http://purl.obolibrary.org/obo/HP
Proteins	http://purl.obolibrary.org/obo/PR
Sequences^b^	http://purl.obolibrary.org/obo/SO
Transcripts	https://uswest.ensembl.org/Homo_sapiens/Transcript/Summary?t=ENST
Vaccines^b^	http://purl.obolibrary.org/obo/VO
Variants	https://www.ncbi.nlm.nih.gov/snp/rs

Note: The node types listed above apply to the PKT Human Disease KG v2.1.0. The node types listed above do not include all of the classes that exist in each Open Biological and Biomedical Ontology (OBO) Foundry ontology. The Cell Ontology is included with the extended version of Uberon.

^a^Two URIs are shown for pathways as the OBO Found ontology is the core ontology used to connect Reactome entities to the core set of OBO Foundry ontologies.

^b^OBO node type. Includes all of the classes that are contained in the ontology even though they are not all explicitly listed here.

Acronyms: CL (Cell ontology); CLO (Cell Line Ontology); CHEBI (Chemical Entities of Biological Interest); GO (Gene Ontology); HPO (Human Phenotype Ontology); MONDO (Mondo Disease Ontology); PKT (PheKnowlator); PRO (Protein Ontology); PW (Pathway Ontology); SO (Sequence Ontology); VO (Vaccine Ontology); UBERON (Uber-Anatomy Ontology).

**Table 3:** PKT Human Disease Knowledge Graph Primary Edge Types by Relation.

Relations	Edge Types
participates in (RO_0000056) has participant (RO_0000057)	chemical-pathway; gene-pathway; protein-biological process; protein-pathway
has function (RO_0000085) function of (RO_0000079)	pathway-molecular function; protein-molecular function
located in (RO_0001025) location of (RO_0001015)	protein-anatomy; protein-cell^a^; protein-cellular component; transcript-anatomy; transcript-cell^a^
has component (RO_0002180)^b^	pathway-cellular component
has phenotype (RO_0002200) phenotype of (RO_0002201)	disease-phenotype
has gene product (RO_0002205) gene product of (RO_0002204)	gene-protein
interacts with (RO_0002434)^c^	chemical-gene; chemical-protein
genetically interacts with (RO_0002435)^c^	gene-gene
molecularly interacts with (RO_0002436)^c^	chemical-biological process; chemical-cellular component; chemical-molecular function; protein-catalyst; protein-cofactor; protein-protein
transcribed to (RO_0002511) transcribed from (RO_0002510)	gene-transcript
ribosomally translates to (RO_0002513) ribosomal Translation of (RO_0002512)	transcript-protein
causally influences (RO_0002566) causally influenced by (RO_0002559)	variant-gene
is substance that treats (RO_0002606) is treated by substance (RO_0002302)	chemical-disease; chemical-phenotype
causes or contributes to condition (RO_0003302)^b^	gene-disease; gene-phenotype; variant-disease; variant-phenotype
realized in response to (RO_0009501)^b^	biological process-pathway

Note: The primary relations and edge types listed above apply to the PKT Human Disease KG v2.1.0. These relations are added to the core set of Open Biological and Biomedical Ontology Foundry ontologies.

^a^The word “cell” above is used to represent cell lines from the Cell Line Ontology and cell types from the Cell Ontology.

^b^Relation Ontology concepts that do not have an inverse.

^c^Relations with symmetrical inverse relations.

Acronyms: PKT (PheKnowLator).

Descriptive statistics for the OBO Foundry ontologies, pre- and post-data quality assessment, are shown in Table 4 (and detailed statistics are provided in Supplementary Table 13). Please note that when reporting results, we will refer to edges as triples, but they both refer to node-relation-node statements. The size of the ontologies varied widely, with the Chemical Entities of Biological Interest (ChEBI)^36^ containing the largest number of triples (n = 5,190,458) and the Protein Ontology (PRO; modified to exclude all non-human proteins)^37^ containing the most classes (n = 148,243). The Relation Ontology (RO)^38^ contained the fewest triples (n =7,873), and the Sequence Ontology (SO)^39^ contained the fewest classes (n = 2,569). The merged set of cleaned OBO Foundry ontologies (i.e., core OBO Foundry ontologies; for additional detail on the ontology cleaning process, please see the Component 1: Knowledge Graph Construction Resources section of the Methods) contained 545,259 classes and 13,748,009 triples. Statistics for triples added to the core OBO Foundry ontologies are listed by edge type in Table 5. The largest edge sets were protein-protein (n = 618,069 triples), transcript-anatomy (n = 439,917 triples), and disease-phenotype (n = 408,702 triples). The smallest edge sets were biological process-pathway (n = 665 triples), gene-gene (n = 1,668 triples), and protein-cofactor (n = 1,961 triples).

**Table 4:** Ontology Statistics Pre- and Post-Data Quality Assessment.

Ontology	Before Cleaning		After Cleaning	
	Classes	Triples	Classes	Triples
Cell Line Ontology	111,712	1,387,096	111,696	1,422,153
Chemical Entities of Biological Interest	156,098	5,264,571	137,592	5,190,485
Gene Ontology	62,237	1,425,434	55,807	1,343,218
Human Phenotype Ontology	38,843	884,999	38,530	885,379
Mondo Disease Ontology	55,478	2,313,343	52,937	2,277,425
Protein Ontology^a^	148,243	2,079,356	148,243	2,079,356
Pathway Ontology	2,642	35,291	2,600	34,901
Relation Ontology	116	7,970	115	7,873
Sequence Ontology	2,910	44,655	2,569	41,980
Uber-Anatomy Ontology^b^	28,738	752,291	27,170	734,768
Vaccine Ontology	7,089	86,454	7,085	89,764
Core OBO Foundry ontologies (merged)^c^	548,947	13,746,883	545,259	13,748,009

Note: The numbers for the ontologies are calculated using the versions of the ontologies that include all imported ontologies referenced by the primary ontology. This means that the counts of classes include all OWL classes used for logical definitions, not only those that are explicitly part of the primary ontology’s namespace.

^a^The Protein Ontology version references the human subset created for the PheKnowLator ecosystem.

^b^The extended version of the Uber-Anatomy Ontology contains the Cell Ontology.

^c^Consistency was evaluated using the ELK reasoner. The reasoner was only applied to individual ontologies.

**Table 5:** PKT Human Disease Knowledge Graph Descriptive Statistics by Primary Edge Type.

Edge	Relation	Subjects	Objects	Standard Relations	Inverse Relations
chemical-disease	substance that treats	4,289	4,494	167,681	335,362
chemical-gene^a^	interacts with	462	11,922	16,639	33,278
chemical-biological process^a^	molecularly interacts with	1,338	1,569	287,068	574,136
chemical-cellular component^a^	molecularly interacts with	1,085	226	40,992	81,984
chemical-molecular function^a^	molecularly interacts with	1,105	200	25,385	50,770
chemical-pathway	participates in	2,104	2,213	28,685	57,370
chemical-phenotype	substance that treats	4,053	1,712	107,962	215,924
chemical-protein^a^	interacts with	4,178	6,379	64,991	129,982
disease-phenotype	has phenotype	11,620	9,714	408,702	817,404
gene-disease^b^	causes or contributes to	5,031	4,420	12,717	–
gene-gene^a^	genetically interacts with	247	263	1,668	3,336
gene-pathway	participates in	10,371	1,809	104,906	209,812
gene-phenotype^b^	causes or contributes to	6,780	1,528	23,501	–
gene-protein	has gene product	19,327	19,143	19,534	39,068
gene-transcript	transcribed to	25,529	179,870	182,736	365,472
biological process-pathway^b^	realized in response to	471	665	665	–
pathway-cellular component^b^	has component	11,134	99	15,846	–
pathway-molecular function	has function	2,412	726	2,416	4,832
protein-anatomy	located in	10,747	68	30,682	61,364
protein-catalyst^a^	molecularly interacts with	3,024	3,730	23,629	47,258
protein-cell^c^	located in	10,045	125	73,530	147,060
protein-cofactor^a^	molecularly interacts with	1,584	44	1,961	3,922
protein-biological process	participates in	17,527	12,246	137,812	275,624
protein-cellular component	located in	18,427	1,757	81,602	163,204
protein-molecular function	has function	17,779	4,324	68,633	137,266
protein-pathway	participates in	10,852	2,468	117,182	234,364
protein-protein^d^	molecularly interacts with	14,320	14,230	618,069	–
transcript-anatomy	located in	29,104	102	439,917	879,834
transcript-cell^c^	located in	14,038	127	64,427	128,854
transcript-protein	ribosomally translates to	44,144	19,200	44,147	88,294
variant-disease^b^	causes or contributes to	13,291	3,565	37,861	–
variant-gene	causally influences	121,790	3,236	121,790	243,580
variant-phenotype^b^	causes or contributes to	1,822	371	2,470	–

Please see Table 3 for Relation Ontology for inverse relations and identifiers.

^a^Symmetric relations were computationally inferred.

^b^The Relation Ontology does not provide an inverse relation.

^c^The word “cell” above is used to represent cell lines from the Cell Line Ontology and cell types from the Cell Ontology.

^d^The data source already included symmetrical edges.

Acronyms: PKT (PheKnowlator).

**Fig. 6:**
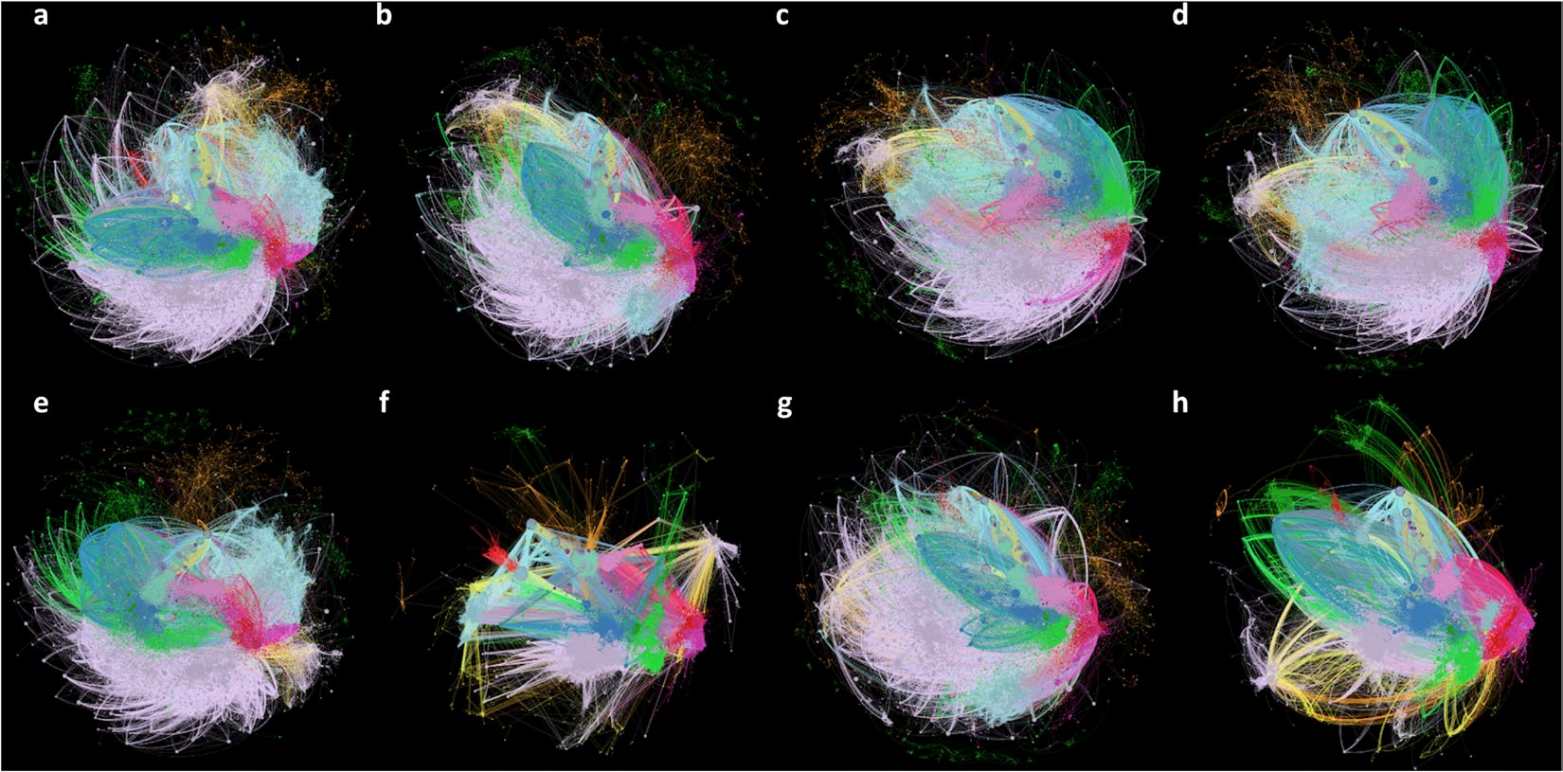
The Impact of Knowledge Model Harmonization on the Semantically Abstracted PKT Human Disease Knowledge Graphs. The figure visualizes the impact of knowledge model harmonization on the semantically abstracted PKT Human Disease benchmark Knowledge Graphs. The top row of figures (**a**–**d**) were built using the class-based knowledge model varying: (**a**) standard relations without harmonization; (**b**) standard relations with harmonization; (**c**) inverse relations without harmonization; (**d**) inverse relations with harmonization. The bottom row of figures (e-h) were built using the instance-based knowledge model varying: (**e**) standard relations without harmonization; (**f**) standard relations with harmonization; (**g**) inverse relations without harmonization; (**h**) inverse relations with harmonization. Nodes are colored by type: anatomical entities (light blue), chemical entities (light purple), diseases (red), genes (purple), genomic features (light green), organisms (yellow), pathways (dark green), phenotypes (magenta), proteins (dark blue), molecular sequences (orange), transcripts (turquoise), and variants (light pink).

Descriptive statistics for the 12 PKT Human Disease benchmark KGs are shown in Table 6. The PKT Human Disease benchmark KGs constructed using the class-based knowledge model with inverse relations and without semantic abstraction were the largest (13,803,521 nodes; 41,116,791 triples). All the PKT Human Disease benchmark KGs built without semantic abstraction, regardless of the knowledge model or relation strategy, contained two connected components and three self-loops. All the PKT Human Disease benchmark KGs were highly sparse, with the average density ranging from 2.16 × 10^−7^ to 3.50 × 10^−7^ and 3.03 × 10^−7^ to 3.40 × 10^−7^ for benchmark KGs constructed using class-based and instance-based knowledge models, respectively. When applying semantic abstraction, the PKT Human Disease benchmark KGs constructed using instance-based knowledge models (743,829 nodes; 4,967,391 to 9,624,232 triples) were on average larger than those constructed using the class-based knowledge models (743,829 nodes; 4,967,427 to 7,629,599 triples). All PKT Human Disease benchmark KGs constructed using the instance-based knowledge model with semantic abstraction, regardless of the relation strategy employed, were larger, had a higher average degree, and contained more self-loops when knowledge model harmonization was applied. The average density (6.68 standard relations; 10.26 inverse relations) and number of self-loops (445 standard and inverse relations) did not differ for the PKT Human Disease benchmark KGs constructed using the class-based knowledge model with semantic abstraction and when applying knowledge model harmonization. The PKT Human Disease benchmark KGs constructed with semantic abstraction, with and without knowledge model harmonization, are visualized in Fig. 6.

**Table 6:** PheKnowLator Human Disease Knowledge Graph Descriptive Statistics.

Knowledge Model	Relation Strategy	Semantic Abstraction	Edges (triples)	Nodes	Relations	Self-Loops	Average Degree
^a^Core OBO Foundry ontologies	N/A	N/A	4,044,658	1,399,756	847	3	2.89
Class-based	Standard Relations	None	25,143,729	8,479,167	847	3	2.97
		Semantic Abstraction Only	4,967,427	743,829	294	445	6.68
		Semantic Abstraction + Harmonization	4,967,429	743,829	293	445	6.68
	Inverse Relations	None	41,116,791	13,803,521	847	3	2.98
		Semantic Abstraction Only	7,629,597	743,829	301	445	10.26
		Semantic Abstraction + Harmonization	7,629,599	743,829	300	445	10.26
Instance-based	Standard Relations	None	21,770,455	8,479,167	847	3	2.57
		Semantic Abstraction Only	4,967,391	743,829	294	409	6.68
		Semantic Abstraction + Harmonization	7,285,496	743,829	293	649	9.79
	Inverse Relations	None	24,432,633	8,479,167	847	3	2.88
		Semantic Abstraction Only	7,629,594	743,829	301	409	10.26
		Semantic Abstraction + Harmonization	9,624,232	743,829	300	650	12.94

Note. Edges and triples are synonymous with respect to the results reported in this table.

^a^Relation Strategy and Semantic Abstraction information are not provided as this row of the table reports information on the core set of merged ontologies.

##### Construction performance

Performance metrics by KG construction step for each of the 12 PKT Human Disease benchmark KGs are shown in Supplementary Figure 2. On average, **Step 1** (*Data Download*) took 2.30 minutes (1.80–3.72 minutes) and used an average of 7.93 GB of memory (7.86–7.99 GB). **Step 2** (*Edge List Creation*) took an average of 4.82 minutes to complete (4.80–4.87 minutes) and used an average of 39.55 GB of memory (38.93–40.43 GB). **Step 3** (*Graph Construction*) took an average of 391.56 minutes (6.53 hours) to complete (265.98–615.92 minutes; 4.43–10.27 hours) and used an average of 118.69 GB of memory (104.30–147.10 GB). On average, the PKT Human Disease benchmark KGs constructed using class-based knowledge models took roughly the same amount of time and used roughly the same maximum amount of memory as those constructed using instance-based knowledge models. Additionally, regardless of the knowledge model, on average, the PKT Human Disease benchmark KGs built using inverse relations and semantic abstraction took longer to run and required more memory.

## Discussion

In this paper, we have presented PheKnowLator, a semantic ecosystem for automating the FAIR construction of ontologically grounded KGs with customizable knowledge representation. The ecosystem includes KG construction resources, analysis tools (i.e., SPARQL endpoint resources and cloud-based APIs), and benchmarks (i.e., prebuilt KGs in multiple formats and embeddings). PheKnowLator enables users to build Semantic Web-compliant complex KGs that are amenable to automatic OWL reasoning, conform to contemporary graph standards, and are importable by popular graph toolkits. By providing flexibility in the way KGs are constructed and generating multiple types of KGs, PheKnowLator also enables the use of cutting-edge graph-based learning and sophisticated inference algorithms. We demonstrated PheKnowLator’s utility by comparing its features to 15 existing open-source KG construction methods and by analyzing its computational performance when constructing 12 different large-scale heterogeneous benchmark KGs. Comparing these methods to PheKnowLator revealed similarities but also highlighted important differentiating factors lacking in other systems, namely: (i) tools to assess the quality of ontologies (which identify, repair, and document syntactic and semantic errors); (ii) logging and metadata documentation (which enable users to debug errors quickly and ensures builds can be rigorously reproduced); and (ii) customizable data preprocessing pipelines (which enable users to use ecosystem tools to develop custom pipelines for processing a wide variety of data, leverage complex mappings, and appropriately resolve missing data), knowledge representation (class- or instance-based), and benchmarks (the ability to construct different types of KGs from the same data, which enables users to empirically evaluate modeling decisions and find the optimal knowledge model or representation for a particular task). These differences highlight PheKnowLator’s ability to provide fully customizable KGs without compromising performance or usability.

One of the biggest challenges to developing novel KG construction methods is properly verifying and robustly validating the resulting KGs. Network-science-based algorithms and machine learning methods typically used within the biomedical domain such as link prediction and KG embedding are able to make use of well-established benchmarks like YAGO^40^, DBPedia, and Wikidata^41^, which are not specific to the biomedical domain. OpenBioLink^28^ was developed as a benchmark for biomedical KGs but is almost exclusively used for link prediction tasks. While it might not be possible to create a universal benchmark to verify or validate biomedical KG construction methods or biomedical KGs, development of trusted resources that are not task-specific (e.g., entity prediction or node classification) would benefit the community. The PheKnowLator ecosystem introduces a set of benchmarks to serve this purpose. These benchmarks were specifically designed to enable two types of tasks: (i) the validation of tools and algorithms designed to analyze KGs (e.g., link prediction algorithms and graph representation learning methods); and (ii) the validation and comparison of KGs built using different underlying knowledge representations. The ability to empirically evaluate knowledge modeling decisions is important when designing knowledge-based systems^8^ and will become more important as more performant graph representation learning methods are developed, especially with respect to explainability^42^.

### PheKnowLator applications and use cases

The majority of existing published KGs and KG construction software within the biomedical domain rely on case studies as a form of evaluation^15,17,19,21,22,26^. While we did not explicitly include case studies as part of our validation, the PheKnowLator ecosystem has fostered substantial collaborations and led to several publications. PheKnowLator benchmark KGs have been used in applications of toxicogenomic mechanistic inference^43^, to enable the exploration of large-scale biomedical hypergraphs^44^, and to facilitate deeper sub-phenotyping of pediatric rare disease patients^45^. Recently, PheKnowLator was used to create a disease-specific KG that combined ontology-grounded resources with literature-derived computable knowledge from machine reading^46^. The resulting KG was then used to identify causal features suitable for addressing confounding bias. PheKnowLator has also been used to generate hypotheses for potential pharmacokinetic natural-product/drug interactions, by facilitating the design and implementation of a KG involving biomedical ontologies, natural-product-ontology extensions, and machine reading from literature^47^. Finally, the PheKnowLator ecosystem was recently selected as the primary infrastructure to facilitate the development of a large-scale KG (denoted RNA-KG) dedicated to the study and development of RNA-based drugs by integrating more than 50 public data sources (https://github.com/AnacletoLAB/RNA-KG)^48^. PheKnowLator is also the foundation for novel KG approaches in microbiome research. The microbe-relevant KG Microbe-Gene-Metabolite Link (MGMLink) was constructed by augmenting PheKnowLator with information on microbes from the integrated database gutMGene. GutMGene relationships describing observed microbe-metabolite or microbe-gene associations were introduced to a PheKnowLator KG, enabling a search space for mechanistic understanding of microbial influence on disease at the molecular level (https://github.com/bsantan/MGMLink).

In addition to the use of the PheKnowLator KG construction software and benchmark KGs, the ecosystem has also contributed to the development of novel tools and resources. Although results are not yet available, PheKnowLator is currently included in the Continuous Evaluation of Relational Learning in Biomedicine (https://biochallenge.bio2vec.net/) task. This task aims to provide a means for evaluating prediction models as new knowledge becomes available over time. Results from this task will provide insight into the usefulness of the PheKnowLator builds and will be used to identify areas where the ecosystem can be improved. Additionally, subsets of prebuilt PheKnowLator KGs have been used to help develop and evaluate novel, cutting-edge graph embedding AI tools (i.e., GRAPE^49^), including random-walk-based embedding methods for extremely large-scale heterogeneous graphs using the PheKnowLator KG builds^50^. In addition to graph representation learning, prebuilt PheKnowLator KGs were used for prototyping a novel method for knowledge-driven mechanistic enrichment of ignorome genes (i.e., differentially expressed genes which are associated with a disease experimentally but that have no known association to the disease in the literature)^51^. When applied to preeclampsia, this method was able to identify 53 novel clinically relevant and biologically actionable disease associations. The National Institutes of Health (NIH) Common Fund Human BioMolecular Atlas Program (HuBMAP)^52^ needed to assemble a KG based on its own preferred graph schema (https://github.com/dbmi-pitt/UMLS-Graph)^53^, with one focus being to maximize the leverage of external references among ontologies for translation (https://github.com/hubmapconsortium/ontology-api). The PheKnowLator ecosystem tool OWL-NETS^34^ is currently being used to ingest other operational ontologies (whether in OWL or not) into HuBMAP and the NIH Common Fund Cellular Senescence Network (SenNet)^54^. PheKnowLator was also applied to methods in generating pathway diagrams using biomedically relevant KGs^55^. This novel approach was able to recapitulate existing figures regarding neuroinflammation and Down Syndrome from literature with more detailed and semantically consistent molecular interactions using PheKnowLator (https://github.com/UCDenver-ccp/Cartoomics).

### Limitations and future work

This current work has several important limitations. First, it is important to point out that the systematic comparison we performed of open-source KG construction methods on GitHub was subjective, included only three researchers actively involved in developing PheKnowLator, and was originally performed in 2020. While the results were updated in 2021 and re-reviewed in 2023, it is possible that new methods might not have been included. Further, only a qualitative comparison was carried out that only considered each method’s GitHub and associated publications. Ideally, a fair evaluation would be performed where each method would be downloaded and compared when used to build a KG from the same set of data. Unfortunately, this type of analysis requires significant resources and was not within the scope of our analysis. Similarly, given their success within the Semantic Web Domain, future work should formally evaluate our data integration and ontology alignment pipelines to tools like Web Karma^56^, OpenRefine (https://openrefine.org/), and mapping languages like R2RML (https://www.w3.org/TR/r2rml/). Second, computational performance metrics were only computed over a single build run due to the number of resources required to build the KGs. While it is not expected that the results for these metrics would significantly change, small deviations related to data provider constraints with respect to accessing build data could result in different outcomes. Third, we mention that the PheKnowLator ecosystem includes two types of benchmarks: KGs and embeddings. Currently, embeddings are only available for one build (v1.0.0^57^) because the size of the generated KGs were quite small. Subsequent builds have resulted in KGs that are so large that generating embeddings has not been feasible. Fortunately, the recent development of performant embedding tools like GRAPE will enable us to provide embeddings for future builds^49^ Fourth, while the ecosystem includes robust logging to monitor metadata and builds, it does not formally integrate resources like the Bioregistry^58^ and BioLink^59^, which are becoming important new KG standards^16,24^. Similarly, the PheKnowLator ecosystem relies heavily on OWLTools (https://github.com/owlcollab/owltools) but newer and more stable tools like ROBOT^60^ should be leveraged because it supports the integration of the OWL API and has improved Jena-based functionality. Fifth, as mentioned above, validating very large KGs, like the ones produced by PheKnowLator, is challenging but important. Additional validation of the PheKnowLator ecosystem, including the construction tools and benchmarks is needed, especially with respect to the different KG builds it produces. Finally, while we have worked hard to ensure that the ecosystem tools and infrastructure are user-friendly, additional work is needed to simplify the inputs and make them more machine-readable (e.g., converting input text files into configurable yaml files) and also develop Graphical User Interfaces for supporting the users in all the steps of KG construction.

## Methods

### The PheKnowLator ecosystem

The PheKnowLator ecosystem was developed to provide a more comprehensive resource to aid in the construction of KGs within the Life Sciences and consists of three components (Fig. 3): (1) **KG Construction Resources**; (2) **Benchmark KGs**; and (3) **KG Tools**. Each component is modular; all features and elements can be replaced or extended as technology evolves or to fit a particular use case. The PheKnowLator ecosystem resources are listed by component in Supplementary Table 3.

#### Component 1: Knowledge graph construction resources

This component is represented by the largest gray box in Fig. 3 and consists of two elements: (1) **Process Data**. Resources to process a variety of heterogeneous data; and (2) **Construct Knowledge Graphs**. An algorithm that enables the construction of different types of heterogeneous KGs. The resources that support these elements are detailed in the *ecosystem Component 1: Knowledge Graph Construction Resources* section of Supplementary Table 3.

##### Process data

This element consists of two features and was designed to help users download and prepare a wide variety of heterogeneous data sources needed to construct KGs. The two primary features of this component are: (i) Download and (ii) Preparation.

**Download**. This feature has been configured to download two types of data: (i) ontologies (e.g., HPO^61^, GO^62^, and PRO^37^) and databases (i.e., a data source not represented as an ontology), which includes Linked Open Data (e.g., Comparative Toxicogenomics Database^63^, UniProt Knowledgebase^64^, STRING^65^), data from molecular experiments (e.g., the Human Protein Atlas^66^, the Genotype-Tissue Expression Project^67^), and existing networks and KGs (e.g., Hetionet^19^, the Monarch KG^68^). Ontologies are downloaded using OWLTools (April 06, 2020 release) and databases are downloaded using a custom-built API capable of processing a variety of file formats (e.g., zip, gzip, tar) from different types of servers and APIs.

**Preparation**. A collection of tools were developed to help users perform a variety of tasks when preparing data that will be used to construct a KG. This feature provides services to map different types of identifiers (e.g., aligning gene identifiers from the Human Gene Nomenclature Committee [HGNC]^69^ to Entrez Gene^70^ and Ensembl^71^), annotate concepts (e.g., convert strings of tissue names from the Human Protein Atlas^66^ to Uber-Anatomy Ontology [Uberon]^72^ concepts), filter data (e.g., identify variant-disease relationships from Clinvar^73^ with a specific type of experimental validation), and process entity metadata (e.g., obtain PubMed identifiers for exposure-outcome relationships from the Comparative Toxicogenomics Database^63^ and extract synonyms and definitions for OBO Foundry ontology concepts). The Data Preparation Notebook (https://github.com/callahantiff/PheKnowLator/blob/master/notebooks/Data_Preparation.ipynb) illustrates some of these features. There are also tools to assess and repair OBO Foundry ontologies, which are known to have a variety of errors^74–76^. The Ontology Cleaning Notebook (https://github.com/callahantiff/PheKnowLator/blob/master/notebooks/Ontology_Cleaning.ipynb) includes detailed descriptions and examples of the data quality checks^77^. A report is generated after assessing the quality of each ontology, which provides statistics before and after applying each check (ontology_cleaning_report.txt).

##### Construct knowledge graphs

This element consists of four features designed to facilitate the construction of large-scale heterogeneous KGs. Together, these features comprise the core functionality of the PheKnowLator KG construction algorithm (referred to as PKT-KG throughout the remainder of the manuscript). The PKT-KG algorithm requires three input documents: (i) a list of one or more OBO Foundry ontologies; (ii) a list of one or more databases; and (iii) edge list assembly instructions (i.e., instructions for filtering input data sources and references to resources needed to normalize concept identifiers). Additional information on each input is available on GitHub (https://github.com/callahantiff/PheKnowLator/wiki/Dependencies). The four primary features of this component are: (i) Edge List Construction, (ii) Ontology Alignment, (iii) Customize Knowledge Representation, and (iv) Output Generation.

**Edge list construction**. Using information in the edge list assembly instructions, the edge list construction procedure merges data, applies filtering and evidence criteria, and removes unneeded attributes. To automate this process, we have developed a universal file parser (and constantly update it with procedures for parsing new file types) that currently processes more than 30 distinct file types. Once the edge lists are constructed, they are serialized in a JSON file.

**Ontology alignment**. OBO Foundry ontologies were selected because they represent canonical knowledge and exist for nearly all scales of biological organization^78^. PKT-KG assumes that every KG is logically grounded^79^ in one or more OBO Foundry ontologies. This feature leverages OWLTools to merge the ontologies into a single integrated core ontology.

**Customize Knowledge Representation**. To enable customization in the way that knowledge is represented when constructing a KG, three configurable parameters are provided:

1. **Knowledge Model**. Following Semantic Web standards^80^, PKT-KG defines a KG as *K* = 〈*T*, *A*〉, where *T* is the TBox and *A* is the ABox. The TBox represents the taxonomy of a particular domain^81,82^. It describes classes, properties or relationships, and assertions that are assumed to generally hold within a domain (e.g., a gene is a heritable unit of DNA located in the nucleus of cells [Fig. 7a]). The ABox describes attributes and roles of instances of classes (i.e., individuals) and assertions about their membership in classes within the TBox (e.g., A2M is a type of gene that may cause Alzheimer’s Disease [Fig. 7b])^81,82^. PKT KGs are logically grounded in one or more OBO Foundry ontology^79^. Database entities (i.e., entities from a data source that is not an OBO Foundry ontology) are added to the core OBO Foundry ontologies using either a TBox (i.e., class-based) or ABox (i.e., instance-based) knowledge model. For the class-based approach, each database entity is made a subclass of an existing core OBO Foundry ontology class (see the “Class-based” section of Supplementary Table 14). For the instance-based approach, each database entity is made an instance of an existing core OBO Foundry ontology class (see the “Instance-based” section of Supplementary Table 14). Both approaches require the alignment of database entities to an existing core OBO Foundry ontology class, which is managed by a dictionary that is constructed using tools in the Process Data Element of the **Knowledge Graph Construction Resources** component (subclass_construction_map.pkl).

**Fig. 7:**
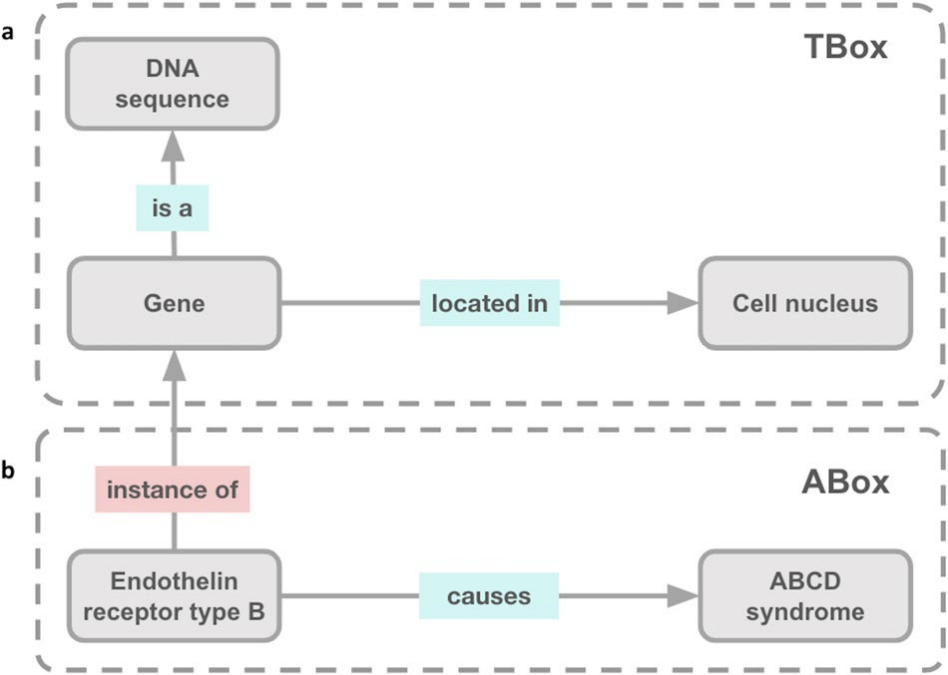
Description Logics Approaches to Knowledge Modeling. This figure provides a simple example of two approaches for modeling knowledge within a Description Logics architecture. (**a**) The TBox includes classes (i.e., “Gene”, “DNA sequence”, and “Cell nucleus”), properties (i.e., “located in” and “is a”), and the assertions between classes (i.e., “Gene is a DNA sequence” and “Gene located in Cell nucleus”). (**b**) The ABox includes instances of classes (i.e., “Endothelin receptor type B”) represented in the TBox and assertions about those instances (i.e., “Endothelin receptor type B, instance of, Gene” and “Endothelin receptor type B, causes, ABCD syndrome”). Please note that this figure is a simplification and was inspired by Fig. 2 from Thessen *et al*.^82^.
2. **Relation Strategy**. PKT-KG provides two relation strategies. The first strategy is standard or directed relations, through a single directed edge (e.g., “gene causes phenotype”). The second strategy is inverse or bidirectional relations, through inference if the relation is from an ontology like the RO (e.g., “chemical participates in pathway” and “pathway has participant chemical”) or through inferring implicitly symmetric relations for edge types that represent biological interactions (e.g., gene-gene interactions).
3. **Semantic Abstraction**. KGs built using expressive languages like OWL are structurally complex and composed of triples or edges that are logically necessary but not biologically meaningful (e.g., anonymous subclasses used to express TBox assertions with all-some quantification). PKT-KG currently uses the OWL-NETS^34^ semantic abstraction algorithm to convert or transform complex KGs into hybrid KGs. OWL-NETS v2.0 (https://github.com/callahantiff/PheKnowLator/wiki/OWL-NETS-2.0) includes additional functionality that harmonizes a semantically abstracted KG to be consistent with a class- or instance-based knowledge model. For class-based knowledge models, all triples containing *rdf:type* are updated to *rdfs:subClassOf*. For instance-based knowledge models, all triples containing *rdfs:subClassOf* are updated to *rdf:type*. For additional details, see OWL-NETS v2.0 documentation.

**Output Generation**. To ensure features of the Process Data element (**KG Construction Resources** component) are transparent and reproducible, metadata are output for all downloaded (downloaded_build_metadata.txt; Supplementary Document 1)) and processed (preprocessed_build_metadata.txt; Supplementary Document 2) data, including the details of the processing steps applied to each database (edge_source_metadata.txt; Supplementary Document 3) and OBO Foundry ontology (ontology_source_metadata.txt and ontology_cleaning_report.txt; Supplementary Documents 4,5). The PKT KG construction process is logged extensively (data download and preprocessing [pkt_builder_phases12_log.log; Supplementary Document 6] and KG construction [pkt_build_log.log; Supplementary Document 7]). PKT KGs, including node and relation metadata, are output to a variety of standard formats. A description of all file types is available from the Zenodo Community archive (PheKnowLator_HumanDiseaseKG_Output_FileInformation.xlsx)^83^.

#### Component 2: Knowledge graph benchmarks

**Fig. 8:**
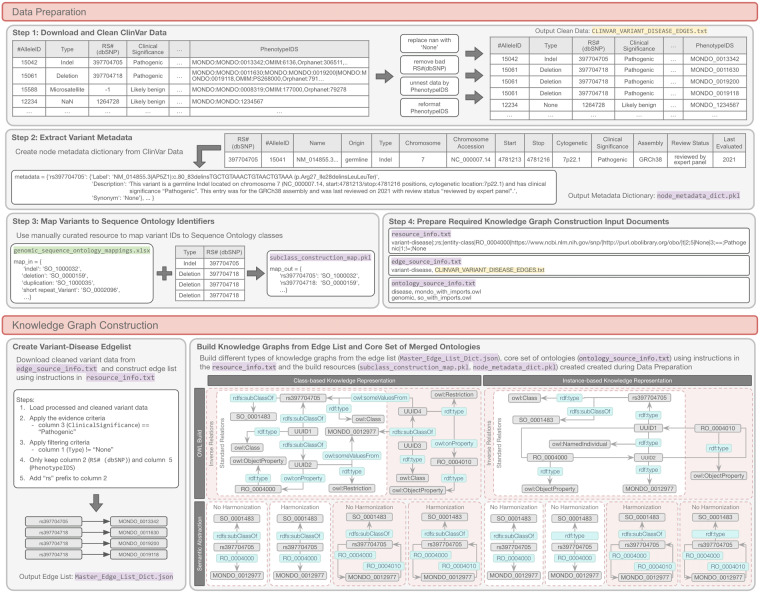
An Example of How Variant-Disease Edges are Created in the PKT Human Disease Mechanism Knowledge Graph. This figure provides an end-to-end example of how variant-disease edges are created in the PKT Human Disease Mechanism knowledge graph. Beginning with the Data Preparation stage, in Step 1, the primary data source (i.e., ClinVar data) is downloaded and cleaned, which includes steps such as replacing “NaN” values with “None”, removing bad or missing identifiers, unnesting the data, and reformatting identifiers. The cleaned data (highlighted in yellow) are output for ingestion into the Knowledge Graph Construction stage. In Step 2, metadata are extracted from the primary data source to create labels, synonyms, and descriptions for each identifier. Step 3 leverages a manually curated resource (highlighted in green) to map variant identifiers to a PKT core ontology. In this case, variant identifiers are aligned to the Sequence Ontology (SO) by their type, and the final mapping is output to subclass_construction_map.pkl which is one of the required inputs for constructing a knowledge graph (highlighted in purple; cited example is from the May 2021 Class-Standard Relation-OWL build). In Step 4, the final step of this stage, the remaining required input documents for constructing a knowledge graph are updated with the resources created in the prior steps. In the Knowledge Graph Construction stage, the cleaned variant data are downloaded and an edge list is built. This edge list can then be used to construct the 12 different knowledge graphs shown in the bottom right gray box. In this example, the class-based semantically abstracted knowledge graphs are the same whether harmonization is applied or not, which is often the case for class-based builds that leverage Open Biological and Biomedical Ontology Foundry ontologies. See the Data_Preparation.ipynb Jupyter Notebook (https://github.com/callahantiff/PheKnowLator/blob/master/notebooks/Data_Preparation.ipynb) for code to process all resources used in the PKT Human Disease knowledge graph. Acronyms: PKT (PheKnowLator). Note. A UUID is a blank or anonymous node that is created from an md5 hash of concatenated Universal Resource Identifiers (URIs). The URIs used in the hash string include the subject and object URIs (each appended with “subject” and “object,” respectively) in addition to a relation. All UUIDs created during construction are explicitly defined within the PKT namespace (https://github.com/callahantiff/PheKnowLator/pkt/).

This component consists of different types of prebuilt KGs or benchmarks that can be used to systematically assess the effects of different knowledge representations on downstream analyses, workflows, and learning algorithms (Fig. 3). Current benchmarks and their supporting features are detailed in the *ecosystem Component 2: Knowledge Graph Benchmarks* section of Supplementary Table 3. Currently, the PheKnowLator ecosystem supports two types of benchmarks: (i) KGs and (ii) embeddings. An end-to-end example demonstrating how a single data source is transformed through each build step of Component 2 is provided in Fig. 8. This figure also demonstrates how this data source would be modeled across the 12 different types of KGs that can be configured from a single build using the ecosystem.

##### Knowledge graphs

The PKT Human Disease KG was built to model mechanisms of human disease, which includes the Central Dogma and represents multiple biological scales of organization including molecular, cellular, tissue, and organ. The knowledge representation was designed in collaboration with a PhD-level molecular biologist (Supplementary Figure 1). The PKT Human Disease KG was constructed using 12 OBO Foundry ontologies, 31 Linked Open Data sets, and results from two large-scale experiments (Supplementary Table 12). The 12 OBO Foundry ontologies were selected to represent chemicals and vaccines (i.e., ChEBI^36^ and Vaccine Ontology [VO]^84,85^), cells and cell lines (i.e., Cell Ontology [CL]^86^, Cell Line Ontology [CLO]^87^), gene/gene product attributes (i.e., Gene Ontology [GO]^62,88^), phenotypes and diseases (i.e., Human Phenotype Ontology [HPO]^61^, Mondo Disease Ontology [Mondo]^89^), proteins, including complexes and isoforms (i.e., PRO^37^), pathways (i.e., Pathway Ontology [PW]^90^), types and attributes of biological sequences (i.e., SO^39^), and anatomical entities (Uberon^72^). The RO^38^ is used to provide relationships between the core OBO Foundry ontologies and database entities. As shown in Fig. 5, the PKT Human Disease KG contained 18 node types (Table 2) and 33 edge types (listed by relation in Table 3). Note that the number of nodes and edge types reflects those that are explicitly added to the core set of OBO Foundry ontologies and does not consider the node and edge types provided by the ontologies. These nodes and edge types were used to construct 12 different PKT Human Disease benchmark KGs by altering the Knowledge Model (i.e., class- vs. instance-based), Relation Strategy (i.e., standard vs. inverse relations), and Semantic Abstraction (i.e., OWL-NETS (yes/no) with and without Knowledge Model harmonization [OWL-NETS Only vs. OWL-NETS + Harmonization]) parameters. Benchmarks within the PheKnowLator ecosystem are different versions of a KG that can be built under alternative knowledge models, relation strategies, and with or without semantic abstraction. They provide users with the ability to evaluate different modeling decisions (based on the prior mentioned parameters) and to examine the impact of these decisions on different downstream tasks.

##### Embeddings

To provide a version of the benchmarks that can more easily be used for downstream learning tasks or to aid in the evaluation of graph-based machine learning algorithms, we have also made some of the monthly builds available with embeddings. A modified version of DeepWalk (https://github.com/xgfs/deepwalk-c) was used to create node embeddings for the v1.0.0 PKT Human Disease benchmark KGs. Embeddings were trained using 128, 256, and 512 dimensions (i.e., the length of the embedding), 100 walks (i.e., the number of paths generated for each node), a walk length of 20 (i.e., the length or number of nodes included in each path), and a sliding window length of 10 (i.e., the number of nodes to the right and left of the target node, which are used as training data for the target node embedding).

Eleven monthly PKT Human Disease benchmark KG builds were created between September 2, 2019 and November 1, 2021, each containing 12 different types of KGs. Each monthly build was executed using GitHub Actions-scheduled Cron jobs and implemented using dedicated Docker containers, which output all data directly to a Google Cloud Storage (GCS) Bucket. The PKT Human Disease benchmark KGs, metadata, and logs are made available through a dedicated Zenodo Community^83^.

#### Component 3: Knowledge graph tools

This component consists of tools to analyze and use KGs (Fig. 3), which includes Jupyter Notebook-based use cases and tutorials, cloud-based data storage, APIs, and triplestores. The features that support these elements are detailed in the *ecosystem Component 3: Knowledge Graph Tools* section of Supplementary Table 3. The Jupyter Notebooks are available on GitHub and currently include tutorials and examples for how to use the OWL-NETS algorithm (https://github.com/callahantiff/PheKnowLator/blob/master/notebooks/OWLNETS_Example_Application.ipynb), load, explore, and modify existing RDF resources (https://github.com/callahantiff/PheKnowLator/blob/master/notebooks/RDF_Graph_Processing_Example.ipynb), and search for paths between two entities in a PKT Human Disease KG (https://github.com/callahantiff/PheKnowLator/blob/master/notebooks/tutorials/entity_search/Entity_Search.ipynb). As mentioned above, KGs are publicly available through the PKT Human Disease benchmark KGs Zenodo Community. Code is provided within the GitHub repository to build and host a SPARQL Endpoint (http://sparql.pheknowlator.com/). The Database Center for Life Science SPARQL proxy web application (https://github.com/dbcls/sparql-proxy) is used as the front end, and the data is served from a Blazegraph triplestore (https://blazegraph.com/).

#### FAIR data principles

The PheKnowLator ecosystem is built on the FAIR principles^35^ (Supplementary Figure 3). **Findability**. Unique persistent identifiers are used for all data (i.e., downloaded, processed, and generated), metadata (i.e., for all downloaded and processed resources, data quality reports, and logged processes), and infrastructure (i.e., Docker containers, compute instances, and KG builds run via GitHub Actions [https://github.com/features/actions] and the Google AI Platform [https://cloud.google.com/ai-platform]). All benchmark KGs are built using standardized and persistent node and relation identifiers. **Accessibility**. All data (i.e., downloaded, processed, and generated), constructed KGs, and metadata generated during the KG construction process, are publicly available and accessible via RESTful API access to a dedicated Zenodo Community archive. Additionally, all builds are versioned on GitHub, Google’s Container Registry (https://cloud.google.com/container-registry), and DockerHub (https://hub.docker.com/). Finally, PheKnowLator provides Jupyter Notebooks and automated dependency generation scripts to improve the usability of its resources. **Interoperability**. The PheKnowLator ecosystem is built on Semantic Web standards, the KGs benchmarks and construction processes are grounded in OBO Foundry ontologies, and, whenever possible, standard identifiers are assigned for all database resources. Additionally, all constructed KGs and KG metadata are output to a variety of standardized file formats like RDF/XML, N-Triples, JSON, and text files. **Reusability**. Benchmark KG builds are automated, containerized, and deployed through GitHub Actions workflows, which makes the build process and resulting KGs consistent across versions. Semantic Versioning (https://semver.org/) is used for all code and documentation. The ecosystem is licensed (Apache-2.0; https://www.apache.org/licenses/LICENSE-2.0), and all ingested data sources are described transparently on the ecosystem’s GitHub Wiki by build version (https://github.com/callahantiff/PheKnowLator/wiki).

### Evaluation

The PheKnowLator ecosystem was evaluated in two ways: (1) **Systematic Comparison of Open-Source KG Construction Software**. Publicly available software to construct biomedical KGs was identified and systematically compared using a survey developed to assess the functionality, availability, usability, maturity, and reproducibility of each method. (2) **Human Disease KG Benchmark Comparison and Construction Performance**. The computational performance of the ecosystem was assessed when used to construct 12 benchmark KGs designed to represent the molecular mechanisms underlying human disease. The resources used for each task are listed in Supplementary Table 4.

#### Systematic comparison of open-source KG construction software

A systematic comparison was performed to examine how the PheKnowLator ecosystem compared to existing open-source biomedical KG construction methods available on GitHub. To provide an unbiased comparison, no assumptions were made regarding a specific set of user requirements. Instead, the goal of the comparison was to provide a detailed overview of existing methods. A survey^91^ was constructed from five criteria (adapted from the evaluation methodology of Babar *et al*.^92^) including: KG construction functionality, maturity, availability, usability, and reproducibility. Example questions used to assess each criterion are provided in Supplementary Table 5. The full set of survey questions (n = 44) are available as a Google Form from Zenodo^91^. Existing open-source biomedical KG construction methods were identified by performing a keyword search against the GitHub API. The following words were combined to form 31 distinct keyword phrases, which were queried against existing GitHub repository descriptions and README content: “biological”, “bio”, “medical”, “biomedical”, “life science”, “semantic”, “knowledge graph”, “kg”, “graph”, “network”, “build”, “construction”, “construct”, “create”, and “creation”. The GitHub scraper is publicly available from Zenodo^93^ and was run in May 2020. The systematic comparison was completed in May 2020 (and updated in June 2021) by TJC with consultation and oversight from WAB and LEH. The survey was scored out of a total score of five points, which was derived as the sum of the ratio of coverage out of one point for each category (i.e., the number of answerable questions out of the number of questions for that category): KG Construction Functionality (10 questions); Availability (two questions); Usability (nine questions); Maturity (five questions); and Reproducibility (six questions). The GitHub scraper and survey results are available from Zenodo^91^.

#### Human disease knowledge graph benchmark comparison and construction performance

Performance metrics were evaluated when building the PKT Human Disease benchmark KGs (v2.1.0 April 11, 2021; testing version not officially released, logs and descriptive statistics available from Zenodo^94^), which included total runtime (minutes) and minimum, maximum, and average memory use (GB). The PKT Human Disease benchmark KGs (v2.1.0 May 1, 2021) were used to compare builds and produce descriptive statistics. Statistics were calculated to help characterize each benchmark KG, including counts of nodes, edges or triples, self-loops, average degree, the number of connected components, and the density. The semantically abstracted (with and without knowledge model harmonization) PKT Human Disease benchmark KGs were visualized and examined for patterns. The v2.1.0_01MAY2021 PKT Human Disease benchmark KGs are publicly available in several formats from Zenodo^95–102^. Additional build details, including data sources, build metadata, and logs, can be found on GitHub (https://github.com/callahantiff/PheKnowLator/wiki/May-01%2C-2021).

### Technical specifications

The PheKnowLator ecosystem resources, including data used to construct KGs and constructed PKT Human Disease benchmark KGs, and code are listed by component in Supplementary Table 3. The PKT Human Disease KG builds were visualized using Gephi^103^ (v0.9.2). The OpenOrd Force-Directed layout^104^ was applied with an edge cut of 0.5, a fixed time of 0.2, and trained for 750 iterations. To help with interpretation, nodes were colored according to node type. When assessing computational performance, all PKT Human Disease KGs were constructed using Docker (v19.03.8) on a Google Cloud Platform N1 Container-Optimized OS instance configured with 24 CPUs, 500 GB of memory, and a 500 GB solid-state drive Boot Disk. PKT Human Disease KG statistics were calculated using Networkx (v2.4).

### Supplementary information

Supplementary Information

## Data Availability

**PKT Human Disease Benchmark KG Archive Resources**. Eleven monthly PKT Human Disease benchmark KG builds were created between September 2, 2019 and November 1, 2021. Each monthly build contains 12 different benchmarks or types of KGs, which were created by altering the following KG construction parameters: knowledge model (i.e., class- or instance-based), relation strategy (i.e., standard directed relations or inverse bidirectional relations), and semantic abstraction (i.e., transformation of complex graphs into OWL-NETS hybrid KGs) with or without knowledge model Harmonization (i.e., ensuring a OWL-NETS KG is consistent with the knowledge model it was abstracted from). The 12 different KG types created by altering these parameters are: 1. Class-based knowledge + Standard Relations + OWL 2. Class-based knowledge + Standard Relations + OWL-NETS 3. Class-based knowledge + Standard Relations + OWL-NETS + Harmonization 4. Class-based knowledge + Inverse Relations + OWL 5. Class-based knowledge + Inverse Relations + OWL-NETS 6. Class-based knowledge + Inverse Relations + OWL-NETS + Harmonization 7. Instance-based knowledge + Standard Relations + OWL 8. Instance-based knowledge + Standard Relations + OWL-NETS 9. Instance-based knowledge + Standard Relations + OWL-NETS + Harmonization 10. Instance-based knowledge + Inverse Relations + OWL 11. Instance-based knowledge + Inverse Relations + OWL-NETS 12. Instance-based knowledge + Inverse Relations + OWL-NETS + Harmonization The builds are available through a Zenodo Community archive (https://zenodo.org/communities/pheknowlator-benchmark-human-disease-kg) with all builds listed and linked on the primary archive page^83^. The monthly builds can also be accessed through the PheKnowLator GitHub Wiki (https://github.com/callahantiff/PheKnowLator/wiki/Archived-Builds). The GitHub Wiki build pages serve as a companion resource to each corresponding Zenodo build archive providing detailed descriptions of the output data files, links to input data sources and Jupyter notebook-based workflows, and lists of generated metadata and logs. Each Wiki build page also includes direct links to each of the 12 benchmark KGs on Zenodo. A detailed description of the build KG files types, including required input documents and curated data, generated build metadata and logs, and output KG data files can be found in the PheKnowLator_HumanDiseaseKG_Output_FileInformation.xlsx^83^ file available on the Zenodo Community archive and from each build’s GitHub Wiki page. This file is intended to provide high-level information on the build file types. It does not cite specific builds or resources but instead, provides explanations of the files that one can expect with each build. Please note that the Zenodo Community archive and associated GitHub pages list 8 KG types rather than 12 as the non-harmonization and harmonization OWL-NETS KG type files are combined into a single repository for each build. Within the Zenodo archives, the harmonized OWL-NETS KGs are referred to as “purified”. **Build data sources**. The curated data sources required for each build are provided in the Zenodo Community archive. All other data are not included with each monthly build due to the large number of required files and their size. These files are all publicly available and can be obtained using information provided with each build, including URL and date of download. For the September 3, 2021 build, links and date of download information are provided on the GitHub Wiki. For the May 01, 2020 build, see the build metadata (edge_source_metadata.txt and ontology_source_metadata.txt) and logs (*_Stats_Terminal_Output.txt), which are all available from the Zenodo Community archive. For all other builds, see downloaded_build_metadata.txt, also available from the Zenodo Community archive. A detailed description of the data sources used to build the PKT Human Disease KG is provided in Supplemental Material Table 12. This table includes the following information for each data source: data provider, filenames, download URLs, literature citations, license types, and a brief description of how each data source was used. **Evaluation resources**. The v2.1.0 May 2021 PKT Human Disease benchmark KGs are available through the GitHub Wiki (https://github.com/callahantiff/PheKnowLator/wiki/May-01%2C-2021), and on Zenodo by KG type: 1. Class-based + StandardRelations + OWL^95^ 2. Class-based + StandardRelations + OWL-NETS^96^ 3. Class-based + StandardRelations + OWL-NETS (purified)^96^ 4. Class-based + InverseRelations + OWL^97^ 5. Class-based + InverseRelations + OWL-NETS^98^ 6. Class-based + InverseRelations + OWL-NETS (purified)^98^ 7. Instance-based + StandardRelations + OWL^99^ 8. Instance-based + StandardRelations + OWL-NETS^100^ 9. Instance-based + StandardRelations + OWL-NETS (purified)^100^ 10. Instance-based + InverseRelations + OWL^101^ 11. Instance-based + InverseRelations + OWL-NETS^102^ 12. Instance-based + InverseRelations + OWL-NETS (purified)^102^ Build logs and statistics are also available for the v2.1.0 April 2021 PKT Human Disease benchmark KGs on Zenodo^94^. As mentioned in the prior section, a table describing the output file types for each build type can be found on the Zenodo Community archive (https://zenodo.org/communities/pheknowlator-benchmark-human-disease-kg)^83^. Descriptions of the data sources used to build the PKT Human Disease KG are provided in Supplemental Material Table 12. As mentioned above, within the Zenodo archives, the harmonized OWL-NETS KGs are referred to as “purified”.

## Code availability

The PheKnowLator ecosystem coding resources are described in detail in Supplementary Table 3 by ecosystem component. The PKT-KG algorithm is publicly available through GitHub (https://github.com/callahantiff/PheKnowLator) and PyPI (https://pypi.org/project/pkt-kg). The SPARQL Endpoint deployment code and documentation are also available through GitHub: https://github.com/callahantiff/PheKnowLator/tree/master/builds/deploy/triple-store#readme. A list of the computational resources used to evaluate the PheKnowLator ecosystem is provided in Supplementary Table 4. The code used to scrape the GitHub API is available from Zenodo^93^. The survey of open-source KG construction tools is also available on Zenodo^91^. The v2.1.0 PheKnowLator code is available on GitHub (https://github.com/callahantiff/PheKnowLator/releases/tag/v2.1.0) and from Zenodo (https://zenodo.org/record/4685943)^105^.
