# Supplementary material for: An open source knowledge graph ecosystem for the life sciences
Source: Sci Data. 2024 Apr 11;11:363. doi: 10.1038/s41597-024-03171-w (PMC11009265; doi:10.1038/s41597-024-03171-w)
Supplement: Supplementary file 1 — Supplementary Information [file 41597_2024_3171_MOESM1_ESM.pdf]

An Open-Source Knowledge Graph Ecosystem for the Life Sciences
SUPPLEMENTARY MATERIAL

Tiffany J. Callahan, Ignacio J. Tripodi, Adrianne L. Stefanski, Luca Cappelletti, Sanya B. Taneja, Jordan M. Wyrwa, Elena Casiraghi, Nicolas A. Matentzoglu, Justin Reese, Jonathan C. Silverstein, Charles Tapley Hoyt, Richard D. Boyce, Scott A. Malec, Deepak R. Unni, Marcin P. Joachimiak, Peter N. Robinson, Christopher J. Mungall, Emanuele Cavalleri, Tommaso Fontana, Giorgio Valentini, Marco Mesiti, Lucas A. Gillenwater, Brook Santangelo, Nicole A. Vasilevsky, Robert Hoehndorf, Tellen D. Bennett, Patrick B. Ryan, George Hripcsak, Michael G. Kahn, Michael Bada, William A. Baumgartner Jr, Lawrence E. Hunter

Table of Contents

Supplementary Tables and Figures **2**
Supplementary Table 1. Important Definitions. 2
Supplementary Table 2. Acronyms used in the Manuscript. 3
Supplementary Table 3. PheKnowLator Ecosystem Resources. 3
Supplementary Table 4. PheKnowLator Ecosystem Evaluation Resources. 6
Supplementary Table 5. Open-Source Knowledge Graph Construction Methods Survey Criteria. 7
Supplementary Table 6. Open-Source Knowledge Graph Construction Methods. 8
Supplementary Table 7. Open-Source Knowledge Graph Construction Survey - Functionality. 10
Supplementary Table 8. Open-Source Knowledge Graph Construction Survey - Availability. 11
Supplementary Table 9. Open-Source Knowledge Graph Construction Survey - Usability. 12
Supplementary Table 10. Open-Source Knowledge Graph Construction Survey - Maturity. 13
Supplementary Table 11. Open-Source Knowledge Graph Construction Survey - Reproducibility. 14
Supplementary Table 12. PKT Human Disease Knowledge Graph Resources - Ontologies. 15
Supplementary Table 13. Application of Data Quality Checks to OBO Foundry Ontologies. 18
Supplementary Table 14. PheKnowLator Knowledge Modeling Approaches. 19
Supplementary Figure 1. Human Disease Mechanism Graph Knowledge Representation. 20
Supplementary Figure 2. PKT Human Disease Knowledge Graph Construction - Computational Performance. 21
Supplementary Figure 3. The PheKnowLator Ecosystem on FAIR Principles. 22
Supplementary Documents **23**
Supplementary Document 1. downloaded_build_metadata.txt. 24
Supplementary Document 2. preprocessed_build_metadata.txt 29
Supplementary Document 3. edge_source_metadata.txt. 33
Supplementary Document 4. ontology_source_metadata.txt. 40
Supplementary Document 5. ontology_cleaning_report.txt. 43
Supplementary Document 6. pkt_builder_phases12_log.log (Data Download and Preparation). 47
Supplementary Document 7. pkt_build_log.log (Knowledge Graph Construction). 51

Supplementary Tables and Figures

Supplementary Table 1. Important Definitions.

Concept	Definition
Database	A data source not represented as an ontology, which can include Linked Open Data, data from experiments, clinical data, and existing networks and knowledge graphs.
Edge	Observed connections between nodes. Edges or triples can also be thought of as node-relation-node statements (e.g., geneA - interacts with - geneB).
Graph	An undirected, unweighted network $G(N,\ L)$, where N is the set of nodes and L is the set of observed edges between these nodes.
Knowledge Graph	A graph-based data structure representing a variety of heterogeneous entities (i.e., nodes) and multiple types of relationships between them and serving as an abstract framework that is able to infer new knowledge to address a variety of applications and use cases.
Knowledge Model	Within the PheKnowLator Ecosystem, there are two types of Knowledge Models that can be used when constructing a knowledge graph (KG): (i) class-based, in which, KGs are constructed using classes, with database entities connected to the core set of merged ontologies as subclasses of existing ontology classes and (ii) instance-based, in which KGs are constructed using instances, with database entities connected to the core set of merged ontologies as instances of existing ontology classes).
Node	Entities or concepts, which are the subject of a knowledge graph. In the biomedical context, nodes usually represent different kinds of biological entities like genes, proteins or diseases.
PKT Human Disease KG	PheKnowLator Ecosystem benchmark knowledge graphs that represent the molecular mechanisms of human disease.
PKT-KG	Phenotype Knowledge TransLator knowledge graph construction algorithm.
Relation	The relationship that connects two nodes in a triple or edge. Relations are used to specify different types of relationships (e.g., interaction, substance that treats) that can exist between a pair of nodes.
Relation Strategy	Within the PheKnowLator Ecosystem, relations can be modeled in two ways when constructing a knowledge graph: (i) Standard Relations (i.e., a unidirectional edge is used to connect a pair of nodes) and (ii) Inverse Relations (i.e., bidirectional edges created by inferring the inverse of relations from ontologies and implicitly symmetric relations like gene-gene interactions).
Semantic Abstraction	Within the PheKnowLator Ecosystem, the OWL-NETS algorithm is used to decode semantically complex OWL-based KGs into KGs that contain biologically meaningful information. Additionally, the Semantic Abstraction parameter, when used with OWL-NETS, includes functionality that can harmonize a KG to a specific kind of Knowledge Model. See the following link for more information: https://github.com/callahantiff/PheKnowLator/wiki/OWL-NETS-2.0.
Harmonization	Ensuring a hybrid KG is consistent with the class- or instance-based complex graph it was abstracted from.

Supplementary Table 2. Acronyms used in the Manuscript.

Concept	Definition
API	Application Programming Interfaces
BioKDE	Biomedical Knowledge Discovery Engine
ChEBI	Chemical Entities of Biological Interest Ontology
CKG	Clinical Knowledge Graph
CL	Cell Ontology
CLO	Cell Line Ontology
FAIR	Findable, Accessible, Interoperable, and Reproducible
GB	Gigabyte
GCS	Google Cloud Storage
GO	Gene Ontology
HGNC	Human Gene Nomenclature Committee
HPO	Human Phenotype Ontology
HuBMAP	Human BioMolecular Atlas Program
KaBOB	Knowledge Base Of Biomedicine
KG	Knowledge Graph
KGTK	Knowledge Graph Toolkit
KGX	Knowledge Graph Exchange
Mondo	Mondo Disease Ontology
MGMLink	Microbe-Gene-Metabolite Link
NDEx	Network Data Exchange
NIH	National Institutes of Health
OBO	Open Biological and Biomedical Ontology
OWL	Web Ontology Language
PheKnowLator	Phenotype Knowledge TransLator
PKT	PheKnowLator
PRO	Protein Ontology
PW	Pathway Ontology
RDF	Resource Description Framework
RDFS	Resource Description Framework Schema
RO	Relation Ontology
SeMi	SEmantic Modeling machIne
SO	Sequence Ontology
SPARQL	SPARQL Protocol and RDF Query Language
Uberon	Uber-Anatomy Ontology
VO	Vaccine Ontology
XML	Extensible Markup Language

Supplementary Table 3. PheKnowLator Ecosystem Resources.

Resource	URL
Ecosystem Component 1: Knowledge Graph Construction Resources	
GitHub	https://github.com/callahantiff/PheKnowLator
PyPI	https://pypi.org/project/pkt-kg/
Docker Container	https://github.com/callahantiff/PheKnowLator/blob/master/Dockerfile
DockerHub	https://hub.docker.com/repository/docker/callahantiff/pheknowlator
GitHub Actions	https://github.com/callahantiff/PheKnowLator/blob/master/.github/workflows/build-qa.yml
Algorithm Dependencies	https://github.com/callahantiff/PheKnowLator/wiki/Dependencies
Dependency Automation Script	https://github.com/callahantiff/PheKnowLator/blob/master/generates_dependency_documents.py
Testing Suite	https://github.com/callahantiff/PheKnowLator/tree/master/tests
Data Processing Jupyter Notebooks	https://github.com/callahantiff/PheKnowLator/blob/master/notebooks/Data_Preparation.ipynb<br>https://github.com/callahantiff/PheKnowLator/blob/master/notebooks/Ontology_Cleaning.ipynb
Ecosystem Component 2: Knowledge Graph Benchmarks	
Human Disease KG Benchmark Details	https://github.com/callahantiff/PheKnowLator/wiki/Benchmarks-and-Builds
Human Disease KG Benchmark Builds Archive	Zenodo<br>- https://zenodo.org/communities/pheknowlator-benchmark-human-disease-kg<br>- Monthly Build Archive: https://<br>- doi.org/10.5281/zenodo.10689968<br>GitHub<br>- https://github.com/callahantiff/PheKnowLator/wiki/Archived-Builds
Human Disease KG Benchmark Builds File Descriptions	https://zenodo.org/records/10065431/files/PheKnowLator_HumanDiseaseKG_Output_FileInformation.xlsx
Knowledge Graph Build Workflow Scripts	
Build Documentation[a]	https://github.com/callahantiff/PheKnowLator/blob/master/builds
Docker Containers	https://github.com/callahantiff/PheKnowLator/blob/master/builds/Dockerfile.phases12<br>https://github.com/callahantiff/PheKnowLator/blob/master/builds/Dockerfile.phase3
GitHub Actions	https://github.com/callahantiff/PheKnowLator/blob/master/.github/workflows/kg-build-part1.yml<br>https://github.com/callahantiff/PheKnowLator/blob/master/.github/workflows/kg-build-part2.yml
Build Requirements	https://github.com/callahantiff/PheKnowLator/blob/master/builds/build_requirements.txt
Build Utilities	https://github.com/callahantiff/PheKnowLator/blob/master/builds/build_utilities.py
Build Logging	https://github.com/callahantiff/PheKnowLator/blob/master/builds/job_monitoring.py<br>https://github.com/callahantiff/PheKnowLator/blob/master/builds/logging.ini
Phase 1 Build Scripts	https://github.com/callahantiff/PheKnowLator/blob/master/builds/phases1_2_entrypoint.py<br>https://github.com/callahantiff/PheKnowLator/blob/master/builds/build_phase_1.py<br>https://github.com/callahantiff/PheKnowLator/blob/master/builds/data_to_download.txt
Phase 2 Build Scripts	https://github.com/callahantiff/PheKnowLator/blob/master/builds/phases1_2_entrypoint.py<br>https://github.com/callahantiff/PheKnowLator/blob/master/builds/build_phase_2.py<br>https://github.com/callahantiff/PheKnowLator/blob/master/builds/data_preprocessing.py<br>https://github.com/callahantiff/PheKnowLator/blob/master/builds/ontology_cleaning.py

Resource	URL
Phase 3 Build Scripts	https://github.com/callahantiff/PheKnowLator/blob/master/builds/build_phase_3.py<br>https://github.com/callahantiff/PheKnowLator/blob/master/builds/phase3_log_daemon.py
Ecosystem Component 3: Knowledge Graphs Tools	
Zenodo Community	https://zenodo.org/communities/pheknowlator-ecosystem
Jupyter Notebooks	https://github.com/callahantiff/PheKnowLator/blob/master/main.ipynb<br>https://github.com/callahantiff/PheKnowLator/blob/master/notebooks/OWLNETS_Example_Application.ipynb<br>https://github.com/callahantiff/PheKnowLator/blob/master/notebooks/RDF_Graph_Processing_Example.ipynb<br>https://github.com/callahantiff/PheKnowLator/blob/master/notebooks/Tutorials/entity_search/Entity_Search.ipynb
SPARQL Endpoint	http://sparql.pheknowlator.com/ (deprecated 2024)<br>Code to facilitate the hosting of a PheKnowLator KG via a SPARQL Endpoint with custom front end web application and serve data from a Blazegraph triple store are available from GitHub: https://github.com/callahantiff/PheKnowLator/tree/36fd2d1dad805a58f4a7fb801b2a7c26dfb130a8/builds/deploy/triple-store#readme

[a]The build documentation provides a detailed description of all of the processes and code needed to generate the knowledge graph builds using Google Cloud Platform resources..

Acronyms: KG (knowledge graph); PKT (PheKnowlator); PKT-KG (PheKnowLator knowledge graph construction software).

Supplementary Table 4. PheKnowLator Ecosystem Evaluation Resources.

Resource	URL
Survey of Open Source KG Construction Software	
GitHub Scraper	https://doi.org/10.5281/zenodo.10052114
Survey	https://doi.org/10.5281/<br>zenodo.10052096
PKT Human Disease KGs	
PKT-KG Zenodo Release	https://doi.org/10.5281/zenodo.4685943
PyPI Release	https://pypi.org/project/pkt-kg/2.1.0/
Data Source Descriptions	https://github.com/callahantiff/PheKnowLator/wiki/May-01%2C-2021
GitHub Actions Workflows	https://github.com/callahantiff/PheKnowLator/.github/workflows/kg-build-part1.yml<br>https://github.com/callahantiff/PheKnowLator/.github/workflows/kg-build-part2.yml
Docker	Data Preparation: https://github.com/callahantiff/PheKnowLator/builds/Dockerfile.phases12<br>KG Construction: https://github.com/callahantiff/PheKnowLator/builds/Dockerfile.phase3
Data Sources[a]	data_to_download.txt
PKT-KG Input Dependencies[a]	edge_source_list.txt<br>ontology_source_list.txt<br>resource_info.txt
Build Metadata[a]	downloaded_build_metadata.txt<br>edge_source_metadata.txt<br>ontology_source_metadata.txt<br>preprocessed_build_metadata.txt
Ontology Quality Report[a]	ontology_cleaning_report.txt
Build Logs[a]	pkt_builder_phases12_log.log<br>pkt_build_log.log
Human Disease KG Benchmark Builds Zenodo Archive	*Class-based Knowledge Model*<br>Standard Relations without Semantic Abstraction: https://doi.org/10.5281/zenodo.10056053<br>Standard Relations with Semantic Abstraction: https://doi.org/10.5281/zenodo.10056054<br>Inverse Relations without Semantic Abstraction: https://doi.org/10.5281/zenodo.10056055<br>Inverse Relations with Semantic Abstraction: https://doi.org/10.5281/zenodo.10056056<br>*Instance-based Knowledge Model*<br>Standard Relations without Semantic Abstraction: https://doi.org/10.5281/zenodo.10056057<br>Standard Relations with Semantic Abstraction: https://doi.org/10.5281/zenodo.10056058<br>Inverse Relations without Semantic Abstraction: https://doi.org/10.5281/zenodo.10056061<br>Inverse Relations with Semantic Abstraction: https://doi.org/10.5281/zenodo.10056062<br><br>A table describing all files for each build KG type can be found on the Zenodo Community archive:<br>https://zenodo.org/records/10065431/files/PheKnowLator_HumanDiseaseKG_Output_FileInformation.xlsx

[a]Each referenced file can be found within each knowledge graph type for every build on Zenodo.

Acronyms: KG (knowledge graph); PKT-KG (PheKnowLator knowledge graph construction algorithm).

Supplementary Table 5. Open-Source Knowledge Graph Construction Methods Survey Criteria.

Criteria	Description	Example Questions
Construction Functionality	An assessment of how well the method covers the steps needed to construct a knowledge graph from downloading and processing data and building edge lists to generating and outputting a KG	Is there functionality to download data?<br>Can multiple types of KGs be constructed?<br>Is preprocessing or filtering performed as part of the construction process?
Maturity	An assessment of the level, stage or development phase of a method	Is a versioning system in place?<br>Have many releases been made?<br>Are procedures in place to enable collaboration?
Availability	An assessment of the openness of a method and the ease of obtaining a copy of the method	Is the method licensed?<br>What type of license is used?
Usability	An assessment of the efforts put in place to ensure that a user, with reasonable technical skills, could use the method	Is there a Wiki, Read the Docs, or GitPage associated with the method?<br>Are there examples of how to use the method?
Reproducibility	An assessment of whether or not the method provides tools or resources to help reproduce the KG construction process and maintain the code base	What tools are provided to help enable reproducibility (e.g., Docker container, Jupyter Notebook, R Markdown)?<br>Does the repository include any form of testing?

Note. The survey questions were adapted from http://dx.doi.org/10.1109/aswec.2004.1290484.

Supplementary Table 6. Open-Source Knowledge Graph Construction Methods.

Method	GitHub Repository[a]	Publication DOI	Primary Goal or Objective (from GitHub)[b]	Method Validation	Most Recent Repository Interaction[c]
Bio2BEL	bio2bel/	10.1101/631812v1	Bio2BEL uses the Biological Expression Language as a common schema for integrating a wide variety of biomedical databases including causal, correlative, and associative relationships between entities on the molecular, process, cellular, systems, and population levels	N/A	Within the last month
Bio2RDF	bio2rdf	10.1016/j.jbi.2008.03.004	Bio2RDF is an open-source project that uses Semantic Web technologies to build and provide the largest network of Linked Data for the Life Sciences	Examined impact of four transcription factors in Parkinson's disease	Within the last week
Bio4J	bio4j/bio4j	10.1101/016758	Bio4j aims to offer a platform for the integration of semantically rich biological data using typed graph models	Tool use demonstration; no formal biological validation	> 1 year
BioGrakn	graknlabs/biograkn	10.1007/978-3-319-61566-0_28	BioGrakn is based on GRAKN.AI, which is a deductive database in the form of a knowledge graph, allowing complex data modelling, verification, scaling, querying and analysis	Illustrative queries spanning precision medicine, text mining, and disease	Within the last month
Clinical Knowledge Graph (CKG)	MannLabs/CKG	10.1101/2020.05.09.084897	Clinical Knowledge Graph is a platform with twofold objectives: 1) build a graph database with experimental data and data imported from diverse biomedical databases and 2) automate knowledge discovery making use of all the information contained in the graph	Biomarker studies to demonstrate CKG use for clinical decision-making	Within the last month
COVID-19-Community	covid-19-net/covid-19-community	NA	The COVID-19-Community is a community effort to build a Neo4j knowledge graph that links heterogenous data about COVID-19	Tool use demonstration	Within the last week
Dipper	monarch-initiative/dipper	NA	Dipper is a Python package to generate RDF triples from common scientific resources	Tool use demonstration	Within the last week
Hetionet	hetio/hetionet	10.7554/eLife.26726	Hetionet is a hetnet — network with multiple node and edge (relationship) types — which encodes biology. Hetnet was designed for Project Rephetio	Predicted the probability of treatment for 209,168 compound–disease pairs	Within the last year
iASiS Open Data Graph	tasosnent/Biomedical-Knowledge-Integration	arXiv:1912.08633	iASiS is a framework to automatically retrieve and integrate disease-specific knowledge into an up-to-date semantic graph	Examined use with lung cancer, dementia, and Duchenne Muscular Dystrophy	Within the last 6 months

Method	GitHub Repository[a]	Publication DOI	Primary Goal or Objective (from GitHub)[b]	Method Validation	Most Recent Repository Interaction[c]
KG-COVID-19	Knowledge-Graph-Hub/kg-covid-19	NA	KG-COVID-19 is a flexible framework to ingest, integrate, and remix biomedical data to produce KGs for COVID-19 response. The framework can be applied to other problems in which siloed biomedical data must be quickly integrated for different biomedical research applications, including for future pandemics	Tool use demonstration	Within the last week
Knowledge Base Of Biomedicine (KaBOB)	UCDenver-ccp/kabob	10.1186/s12859-015-0559-3	KaBOB is a knowledge base of semantically integrated data. The system introduces five processes for semantic data integration including making explicit the differences between biomedical concepts and database records, aggregating sets of identifiers denoting the same biomedical concepts across data sources, and using declaratively represented forward-chaining rules to take information that is variably represented in source databases and integrating it into a consistent biomedical representation	Constructed a multi-species KG	Within the last year
Knowledge Graph Exchange (KGX)	NCATS-Tangerine/kgx	NA	KGX is a library and set of command line utilities for exchanging Knowledge Graphs that conform to or are aligned to the Biolink Model	Tool use demonstration	Within the last month
Knowledge Graph Toolkit (KGTK)	usc-isi-i2/kgtk/	arXiv:2006.00088	KGTK is a data science-centric toolkit to represent, create, transform, enhance and analyze KGs. KGTK represents graphs in tables and leverages popular libraries developed for data science applications, enabling a wide audience of developers to easily construct KG pipelines for their applications	Demonstrated functionality using Wikidata, DBpedia, and ConceptNet	Within the last week
ProNet	cran/ProNet	NA	ProNet provides functions for biological network construction, visualization and analyses, including topological statistics, functional module clustering, and GO-profiling	Examined H1N1 IAV-human protein-protein interactions	> 1 year
SEmantic Modeling machIne (SeMi)	giuseppefutia/semi	10.1016/j.softx.2020.100516	SeMi (SEmantic Modeling machIne) is a tool to semi-automatically build large-scale Knowledge Graphs from structured sources such as CSV, JSON, and XML files	Validated using advertising data	Within the last 6 months
PheKnowLator	PheKnowLator	10.1101/2020.04.30.071407	PheKnowLator (Phenotype Knowledge Translator) is a novel framework and fully automated Python 3 library explicitly designed for optimized construction of semantically-rich, large-scale biomedical KGs	Built and compared 12 benchmark KGs including construction performance	Within the last week

[a]All GitHub URLs begin with the following prefix: https://github.com/.
[b]Whenever possible, descriptions of methods and tools were copied verbatim from the associated GitHub site, documentation, and/or manuscript. Only minor edits or modifications were applied.
[c]The most recent repository interaction was documented at the time of completing the survey, which was May 2020 (updated in June 2021).
Acronyms: KG (Knowledge Graph).

Supplementary Table 7. Open-Source Knowledge Graph Construction Survey - Functionality.

Method	Download Functionality	Edge list Functionality	Construction Functionality	Multiple KG Types	Other KG Construction Functionality	Process Ontology Data	Process Linked Open Data	Process Experimental Data	Process Clinical Data	Data Processing Limits
Bio2BEL	Yes	Yes	Yes	Yes	The entire PyBEL ecosystem tools are all available for all graphs generated by Bio2BEL	Yes	Yes	Yes	Yes	No
Bio2RDF	Yes	Yes	Yes	Yes	Talend RESTful API; community ontology mappings; SPARQL query repository	Yes	Yes	Yes	Yes	No
Bio4J	Yes	Yes	Yes	Yes	Titan, Angulillos API	Yes	Yes	No	No	No
BioGrakn	No	No	Yes	Yes	Provides different types of API clients (Java, Python, Node.js) and a Grakn Workbase	No	Yes	Yes	Yes	No
Clinical Knowledge Graph (CKG)	Yes	No	Yes	No	Data preparation (filtering, imputation, formattin); data analysis (dimensionality reduction, visualization, hypothesis testing)	Yes	Yes	Yes	No	No
COVID-19-Community	Yes	Yes	Yes	No	Neo4J Browser	No	Yes	No	Yes	No
Dipper	Yes	Yes	Yes	Yes	SciGraph RESTful API<br>Build KGs with evidence and provenance	Yes	Yes	Yes	No	No
Hetionet	Yes	No	Yes	No	Neo4J Browser<br>Creates permuted KGs	Yes	Yes	Yes	Yes	No
iASiS Open Data Graph	Yes	Yes	Yes	No	Biomedical Harvesters; MedKnow	Yes	Yes	No	Yes	No
KG-COVID-19	Yes	Yes	Yes	No	Leverages BioLink	Yes	Yes	No	No	No
Knowledge Base Of Biomedicine (KaBOB)	Yes	Yes	Yes	Yes	Blazegraph	Yes	Yes	No	No	No
Knowledge Graph Exchange (KGX)	Yes	Yes	Yes	Yes	KG verified to confirm to the Biolink model, summary statistics	Yes	Yes	Yes	No	No
Knowledge Graph Toolkit (KGTK)	Yes	Yes	Yes	Yes	Data cleaning module, processes other KGs, KG querying modules, summary statistics, node embeddings	No	Yes	No	No	No
ProNet	No	Yes	Yes	No	KG visualization; enables topological analyses	No	Yes	Yes	No	No
SEmantic Modeling machIne (SeMi)	No	Yes	Yes	Yes	Semantic type detector; weighted graph generator; semantic model builder and refiner; link predictor	Yes	Yes	No	No	No
PheKnowLator	Yes	Yes	Yes	Yes	Data download and preprocessing tools; ontology quality control tools; export node metadata; property graphs; SPARQL Endpoint	Yes	Yes	Yes	No	No

Note. For scoring, 1 point was awarded for an answer of "Yes" and for the presence of other KG construction functionality.

Acronyms: KG (Knowledge Graph).

Supplementary Table 8. Open-Source Knowledge Graph Construction Survey - Availability.

Method	Open Source	License	Operating Systems	Coding Languages	External Dependencies
Bio2BEL	Yes	MIT	Linux, Windows, Mac OSX, Cloud-based systems and/or architectures	Python, SQL	Bioregistry, PyOBO, Bioversions, various other standard Python packages
Bio2RDF	Yes	MIT<br>Apache 2.0<br>CC0-1.0	Linux, Windows, Mac OSX	Java, JavaScript, Shell, OWL	Virtuoso, GIT
Bio4J	No	AGPL-3.0	Linux, Windows, Mac OSX, Cloud-based systems and/or architectures	Java, Scala	Angulillos, AWS EC2/S3, Titan
BioGrakn	No	None	Linux, Windows, Mac OSX, Cloud-based architectures	Python, Java, Node.js	GraknLabs, Maven
Clinical Knowledge Graph (CKG)	Yes	MIT	Linux, Windows, Mac OSX	Python	Java SE Runtime, Neo4j, R, Python 3.6
COVID-19-Community	No	MIT	Linux, Windows, Mac OSX	Python, Shell	Neo4J, Anaconda
Dipper	No	BSD-3	Linux, Windows, Mac OSX	Python, TSQL	
Hetionet	Yes	CC0	Linux, Windows, Mac OSX	Python, Shell	Docker, Neo4J
iASiS Open Data Graph	No	Apache 2.0	Linux, Windows, Mac OSX	Python, Java	MongoDB, UMLS, ReVerb, MetaMap, SemRep, YAJL, Neo4J
KG-COVID-19	Yes	BSD-3	Linux, Windows, Mac OSX	Python	KGX, BioLink
Knowledge Base Of Biomedicine (KaBOB)	Yes	GPL	Linux, Windows, Mac OSX	Groovy, Clojure, Shell	Docker, Maven
Knowledge Graph Exchange (KGX)	Yes	BSD-3	Linux, Windows, Mac OSX	Python	Docker, BioLink
Knowledge Graph Toolkit (KGTK)	Yes	MIT	Linux, Windows, Mac OSX	Python	Anaconda, mlr
ProNet	Yes	GPL (>=2)	Linux, Windows, Mac OSX	R	BioGrid, GO
SEmantic Modeling machIne (SeMi)	Yes	GPL	Linux, Windows, Mac OSX	Python, JavaScript, Shell	Anaconda, Node.js (11.15.0), Java, Maven, Elasticsearch
PheKnowLator	Yes	Apache 2.0	Linux, Windows, Mac OSX, Cloud-based systems and/or architectures	Python, Java, Shell	OWL Tools

Note. For scoring, 1 point was awarded for an answer of “Yes” and for the presence of a license.

Supplementary Table 9. Open-Source Knowledge Graph Construction Survey - Usability.

Method	README	Wiki, Docs, or GitPage	Example Use	Tutorials	Install Tools	Method Use Resources	Sample Data	Handles Different Sized Data	Output Types	Adoption Indicators
Bio2BEL	Yes	Yes	Yes	No	PyPI<br>Maven	None	Yes	Yes	NetworkX, Cytoscape, text files, n-triples, Biological Expression Language, several IO formats	Yes
Bio2RDF	Yes	Yes	Yes	No	None	None	Yes	Yes	Virtuoso dump, OWL, nq	Yes
Bio4J	Yes	Yes	Yes	Yes	AWS S3	Angulillos API<br>Titan	Yes	Yes	Titan	Yes
BioGrakn	Yes	Yes	Yes	Yes	None	Grakn Clients	Yes	Yes	Grakn KG output types	Yes
Clinical Knowledge Graph (CKG)	Yes	Yes	Yes	Yes	Docker	Jupyter Notebook<br>Docker	Yes	Yes	Neo4j	Yes
COVID-19-Community	Yes	No	Yes	Yes	Jupyter Notebook	Jupyter Notebooks	Yes	Yes	Neo4J, CSV	Yes
Dipper	Yes	Yes	Yes	Yes	PYPI	Jupyter Notebooks	Yes	Yes	TTL, Neo4J, TSV	Yes
Hetionet	Yes	Yes	Yes	Yes	Jupyter Notebook	Jupyter Notebook<br>Docker	Yes	Yes	JSON, Neo4J, TSV, and Matrix	Yes
iASiS Open Data Graph	Yes	Yes	Yes	No	None	None	No	Yes	JSON, CSV, Neo4J, MongoDB	Yes
KG-COVID-19	Yes	Yes	Yes	Yes	None	None	Yes	Yes	RDF, TSV	Yes
Knowledge Base Of Biomedicine (KaBOB)	Yes	Yes	Yes	Yes	Docker	Docker	Yes	Yes	RDF/XML	Yes
Knowledge Graph Exchange (KGX)	Yes	Yes	Yes	Yes	PyPI<br>Docker	None	Yes	Yes	OWL or RDF/XML, NetworkX, text files, n-triples files, tar, csv, graphML, TTL, JSON, RQ, RSA	Yes
Knowledge Graph Toolkit (KGTK)	Yes	Yes	Yes	Yes	Jupyter Notebook<br>Docker	Jupyter Notebook<br>Docker	Yes	Yes	n-triples files, JSON, Neo4J, GML	Yes
ProNet	No	Yes	Yes	Yes	CRAN	R Markdown	Yes	Yes	R data frame object (rda)	No
SEmantic Modeling machIne (SeMi)	Yes	Yes	Yes	No	PyPI	None	Yes	Yes	OWL or RDF/XML files, graph, json, TTL	No
PheKnowLator	Yes	Yes	Yes	Yes	PyPI<br>Jupyter Notebook<br>Docker	Jupyter Notebook<br>Docker	Yes	Yes	RDF/XML, NetworkX, a text files, n-triples, JSON	Yes

Note. For scoring, 1 point was awarded for an answer of "Yes" and for the presence of tools to run and install the method.

Supplementary Table 10. Open-Source Knowledge Graph Construction Survey - Maturity.

Method	Multiple Releases	Release Count	Method Published	Collaboration Encouraged	Collaboration Procedures
Bio2BEL	Yes	1	Yes	Yes	Yes
Bio2RDF	Yes	2	Yes	Yes	No
Bio4J	Yes	100	Yes	No	Yes
BioGrakn	Yes	1	Yes	No	No
Clinical Knowledge Graph (CKG)	No	0	Yes	Yes	Yes
COVID-19-Community	No	0	No	Yes	Yes
Dipper	Yes	4	No	No	No
Hetionet	No	1	Yes	Yes	No
iASiS Open Data Graph	No	0	Yes	No	No
KG-COVID-19	No	0	No	Yes	Yes
Knowledge Base Of Biomedicine (KaBOB)	No	1	Yes	No	No
Knowledge Graph Exchange (KGX)	No	0	No	No	No
Knowledge Graph Toolkit (KGTK)	Yes	3	Yes	Yes	Yes
ProNet	Yes	1	Unclear	No	No
SEmantic Modeling machIne (SeMi)	No	0	Yes	No	No
PheKnowLator	Yes	1	Yes	Yes	Yes

Note. For scoring, 1 point was awarded for an answer of “Yes” and for the presence of at least one release.

Supplementary Table 11. Open-Source Knowledge Graph Construction Survey - Reproducibility.

Method	Reproducibility Tools	Install Services	Deployment Services	Maintainability Measures	Well-Documented Codebase	Actively Used Issue Tracker
Bio2BEL	CLI Tool	Yes	No	Yes	Yes	Yes
Bio2RDF	None	No	No	No	Yes	Yes
Bio4J	AWS S3 Titan distribution	No	Yes	No	Yes	Yes
BioGrakn	Grakn Tools	Yes	Yes	No	Yes	Yes
Clinical Knowledge Graph (CKG)	Jupyter Notebook Docker	No	No	No	Yes	Yes
COVID-19-Community	Jupyter Notebooks	No	No	No	Yes	Yes
Dipper	Jupyter Notebook	Yes	Yes	No	Yes	Yes
Hetionet	Jupyter Notebook Docker	No	No	No	Yes	Yes
iASiS Open Data Graph	None	Partial	No	No	Yes	Yes
KG-COVID-19	None	Yes	Yes	Yes	Yes	Yes
Knowledge Base Of Biomedicine (KaBOB)	Docker	Yes	Yes	No	Yes	Yes
Knowledge Graph Exchange (KGX)	Jupyter Notebook Docker	Yes	Yes	No	Yes	Yes
Knowledge Graph Toolkit (KGTK)	Jupyter Notebook Docker	Yes	Yes	Yes	Yes	Yes
ProNet	R Markdown	No	No	No	Yes	No
SEmantic Modeling machIne (SeMi)	None	Yes	Yes	No	Yes	Yes
PheKnowLator	Docker Jupyter Notebook	Yes	Yes	Yes	Yes	Yes

Note. For scoring, 1 point was awarded for an answer of “Yes” and for the presence of at least one reproducibility tool.

Supplementary Table 12. PKT Human Disease Knowledge Graph Resources - Ontologies.

Provider	Filename	URLs and Citations	License	Node or Edge Type and Usage
ONTOLOGY RESOURCES				
Chemical Entities of Biological Interest (ChEBI)	http://purl.obolibrary.org/obo/chebi.owl	**URL:** https://www.ebi.ac.uk/chebi/<br>**Citation:** PMID:26467479	CC BY 4.0	Utilized to connect chemicals to complexes, diseases, genes, GO biological processes, GO cellular components, GO molecular functions, pathways, phenotypes, reactions, and transcripts.
Cell Ontology (CL)[a]	http://purl.obolibrary.org/obo/uberon/ext.owl	**URL:** https://github.com/obophenotype/cell-ontology<br>**Citation:** PMID:27377652	CC BY 4.0	Utilized to connect transcripts and proteins to cells. Additionally, this ontology imports the following ontologies: ChEBI, GO, PATO, PRO, RO, Uberon.
Cell Line Ontology (CLO)	http://purl.obolibrary.org/obo/clo.owl	**URL:** https://obofoundry.org/ontology/clo.html<br>**Citation:** PMID:25852852	CC BY 3.0	Utilized to map cell lines to transcripts and proteins. Additionally, this ontology imports the following ontologies: CL, DOID, NCBITaxon, Uberon.
Gene Ontology (GO)	http://purl.obolibrary.org/obo/go.owl	**URL:** http://geneontology.org/<br>**Citations:** PMID:10802651; PMID:36866529	CC BY 4.0	Utilized to connect biological processes, cellular components, and molecular functions to chemicals, pathways, and proteins. Additionally, this ontology imports the following ontologies: CL, NCBITaxon, RO, and Uberon.
Human Phenotype Ontology (HPO)	http://purl.obolibrary.org/obo/hp.owl	**URL:** https://ontology.jax.org/api/hp/docs/<br>**Citation:** PMID:33264411	MIT License	Utilized to connect phenotypes to chemicals, diseases, genes, and variants. Additionally, this ontology imports the following ontologies: CL, ChEBI, GO, and Uberon.
Mondo Disease Ontology (Mondo)	http://purl.obolibrary.org/obo/mondo.owl	**URL:** https://mondo.monarchinitiative.org/<br>**Citation:** DOI:10.1101/2022.04.13.22273750	CC BY 4.0	Utilized to connect diseases to chemicals, phenotypes, genes, and variants. Additionally, this ontology imports the following ontologies: CL, NCBITaxon, GO, HPO, and Uberon.
Pathway Ontology (PW)	http://purl.obolibrary.org/obo/pw.owl	**URL:** https://rgd.mcw.edu/wg/home/pathway2/<br>**Citation:** PMID:24499703	CC BY 4.0	Utilized to connect pathways to GO biological processes, GO cellular components, GO molecular functions,and Reactome pathways.
Protein Ontology (PRO)	http://purl.obolibrary.org/obo/pr.owl	**URL:** https://proconsortium.org/<br>**Citation:** PMID:20935045	CC BY 4.0	Utilized to connect proteins to chemicals, genes, anatomy, catalysts, cell lines, cofactors, complexes, GO biological processes, GO cellular components, GO molecular functions, pathways, proteins, reactions, and transcripts. Additionally, this ontology imports the following ontologies: ChEBI, DOID, and GO. The ontology was subset to only include homo sapien proteins.
Relations Ontology (RO)	http://purl.obolibrary.org/obo/ro.owl	**URL:** https://github.com/oborel/obo-relations/<br>**Citation:** PMID:15892874	CC 1.0 Universal	Utilized this to connect all data sources added to the core set of merged ontologies.
Sequence Ontology (SO)	http://purl.obolibrary.org/obo/so.owl	**URL:** https://github.com/The-Sequence-Ontology/SO-Ontologies<br>**Citation:** PMID:15892872	CC BY 4.0	Utilized to connect transcripts and other genomic material like genes and variants.
Uber-Anatomy Ontology (Uberon)	http://purl.obolibrary.org/obo/uberon/ext.owl	**URL:** http://obophenotype.github.io/uberon/<br>**Citation:** PMID:22293552; PMID: 25009735	CC BY 3.0	Utilized to connect tissues, fluids, and cells to proteins and transcripts. Additionally, this ontology imports the following ontologies: ChEBI, CL, GO, PRO.
Vaccine Ontology (VO)	http://purl.obolibrary.org/obo/vo.owl	**URL:** https://github.com/vaccineontology/VO	CC BY 3.0	Utilized the edges between this ontology and those it

Provider	Filename	URLs and Citations	License	Node or Edge Type and Usage
		**Citation:** PMID:23256535; PMID:21624163		imports: ChEBI, DOID, GO, PRO, and Uberon.
EDGE SET RESOURCES				
ClinVar	variant_summary.txt	**URL:** https://ftp.ncbi.nlm.nih.gov/pub/clinvar<br>**Citation:** PMID:29165669	MIT License	variant-gene; variant-disease; variant-phenotype
Comparative Toxicogenomics Database (CTD)	CTD_chemicals_diseases.tsv<br>CTD_chem_gene_ixns.tsv<br>CTD_chem_go_enriched.tsv<br>CTD_genes_pathways.tsv	**URL:** http://ctdbase.org/<br>**Citation:** PMID:36169237	CC BY 4.0	chemical-disease; chemical-phenotype<br>chemical-gene; chemical-protein<br>chemical-biological process; chemical-cellular component; chemical-molecular function<br>gene-pathway
DisGeNET	Curated_gene_disease_associations.tsv	**URL:** https://www.disgenet.org/<br>**Citation:** PMID:31680165	CC BY-NC-SA 4.0	gene-disease; gene-phenotype
Ensembl	Homo_sapiens.GRCh38.102.gtf<br>Homo_sapiens.GRCh38.102.uniprot.tsv.gz<br>Homo_sapiens.GRCh38.102.entrez.tsv.gz	**URL:** https://useast.ensembl.org/<br>**Citation:** PMID:36318249	Apache 2.0	gene-protein; gene-transcript; transcript-protein
Gene MANIA	COMBINED.DEFAULT_NETWORKS.BP_COMBINING.txt	**URL:** https://genemania.org/<br>**Citation:** PMID:20576703	CC BY 4.0	gene-gene
Gene Ontology	goa_human.gaf	**URL:** http://geneontology.org/<br>**Citation:** PMID:10802651; PMID:36866529	CC BY 4.0	protein-biological process; protein-cellular component; protein-molecular function
The Genotype-Tissue Expression (GTEx) Project	https://storage.googleapis.com/gtex_analysis_v8/rna_seq_data/GTEx_Analysis_2017-06-05_v8_RNASeQCv1.1.9_gene_median_tpm.gct.gz	**URL:** https://gtexportal.org/home/<br>**Citation:** PMID:23715323	CC BY 4.0	protein-anatomy; protein-cell; transcript-anatomy; transcript-cell
HUGO Gene Nomenclature Committee (HGNC)	hgnc_complete_set.txt	**URL:** https://www.genenames.org/<br>**Citation:** PMID:36243972	CC0	gene-protein; gene-transcript; transcript-protein
The Human Phenotype Ontology (HPO)	phenotype.hpoa	**URL:** https://hpo.jax.org/<br>**Citation:** PMID:33264411	MIT License	disease-phenotype
The Human Protein Atlas	API Query	**URL:** https://www.proteinatlas.org/<br>**Citation:** PubMed:25613900	CC BY 3.0	protein-anatomy; protein-cell; transcript-anatomy; transcript-cell
The National Center for Biotechnology Information (NCBI)	Homo_sapiens.gene_info.gz	**URL:** https://www.ncbi.nlm.nih.gov/gene/<br>**Citation:** PMID:21115458	Terms and Conditions	gene-protein; gene-transcript; transcript-protein
Reactome Pathway Database	ChEBI2Reactome_All_Levels.txt<br>gene_association.reactome<br>UniProt2Reactome_All_Levels.txt	**URL:** https://reactome.org/<br>**Citation:** PMID:34788843	CC0	chemical-pathway<br>biological processes-pathway; pathway-cellular component; pathway-molecular function<br>protein-pathway
The Search Tool for Recurring Instances of	9606.protein.links.v11.0.txt	**URL:** https://string-db.org/<br>**Citation:** PMID:36370105	CC BY 4.0	protein-protein

Provider	Filename	URLs and Citations	License	Node or Edge Type and Usage
Neighbouring Genes (STRING) Database				
Universal Protein Resource (UniProt)	API Query	**URL:** https://www.uniprot.org/ **Citation:** PMID:36408920	CC BY 4.0	gene-protein; gene-transcript; transcript-protein protein-catalyst; protein-cofactor
MAPPING AND FILTERING RESOURCES				
Chemical Entities of Biological Interest (ChEBI)	names.tsv	**URL:** https://www.ebi.ac.uk/chebi/ **Citation:** PMID:26467479	CC BY 4.0	Used in combination with MeSH to to obtain mappings between MeSH identifiers and ChEBI identifiers for chemicals-diseases, chemicals-genes, chemical-GO biological processes, chemicals-GO cellular components, chemicals-GO molecular functions, chemicals-phenotypes, chemicals-proteins, and chemicals-transcripts.
Compath	curated_mappings.txt kegg_reactome.csv	**URLs:**<br>- https://compath.scai.fraunhofer.de/<br>- https://github.com/ComPath/compath-resources/tree/master/mappings<br>**Citation:** PMID:30564458	Imprint MIT License	To align Reactome pathway concept identifiers to PW.
DisGeNET	disease_mappings.tsv	**URL:** https://www.disgenet.org/ **Citation:** PMID:31680165	CC BY-NC-SA 4.0	Obtain mappings between different disease terminologies and vocabularies including: DOID, OMIM, Orphanet, ICD9, ICD10, the UMLS and MeSH to Mondo and the HPO.
The Medical Subject Headings (MeSH)	mesh2021.nt	**URL:** https://www.nlm.nih.gov/mesh/meshhome.html **Citation:** PMID:13982385	Terms and Conditions	Used in combination with ChEBI to obtain mappings between MeSH identifiers and ChEBI identifiers for chemicals-diseases, chemicals-genes, chemical-GO biological processes, chemicals-GO cellular components, chemicals-GO molecular functions, chemicals-phenotypes, chemicals-proteins, and chemicals-transcripts.
Protein Ontology (PRO)	pro_mapping.txt	**URL:** https://proconsortium.org/ **Citation:** PMID:20935045	CC BY 4.0	To obtain mappings between PRO ontology concepts to other protein, gene, and transcript identifiers form UniProt, Entrez Gene, HGNC,
Universal Protein Resource (UniProt)	API Query	**URL:** https://www.uniprot.org/ **Citation:** PMID:36408920	CC BY 4.0	To obtain mappings between PRO ontology concepts to other protein, gene, and transcript identifiers from the PRO, Entrez Gene, HGNC,

Note. Sources are reported for the v.2.1.0 knowledge graphs (built May 2021). The full URLs are provided here: https://github.com/callahantiff/PheKnowLator/blob/549e6e1e882e9ea579508ae24a90e64d962deb8c/builds/data_to_download.txt.
[a]The Cell Ontology is included with the extended version of Uberon.

Acronyms: CL (Cell ontology); CLO (Cell Line Ontology); ChEBI (Chemical Entities of Biological Interest); CTD (Comparative Toxicogenomics Database); DOID (Human Disease Ontology); GOOGLE_CLOUD_STORAGE (Google Cloud Storage); GO (Gene Ontology); HGNC (Human Gene Nomenclature Committee); HPO (Human Phenotype Ontology); HPA (Human Protein Atlas); ICD (International Classification of Diseases); MeSH (Medical Subject Headings); Mondo (Mondo Disease Ontology); OMIM (Online Mendelian Inheritance in Man); PRO (Protein Ontology); PRO (Protein Ontology); PW (Pathway Ontology); SO (Sequence Ontology); VO (Vaccine Ontology); Uberon (Uber-Anatomy Ontology); UMLS (Unified Medical Language System).

Supplementary Table 13. Application of Data Quality Checks to OBO Foundry Ontologies.

Statistics[a]	CLO	ChEBI	GO	HPO	Mondo	PRO[b]	PW	RO	SO	Uberon	VO	Merged[c]
Pre-Processed Statistics												
Edges	1,387,096	5,264,571	1,425,434	884,999	2,313,343	2,079,356	35,291	7,970	44,655	752,291	86,454	13,746,883
Classes	111,712	156,098	62,237	38,843	55,478	148,243	2,642	116	2,910	28,738	7,089	548,947
Individuals	41	0	0	0	18	0	0	5	0	0	165	195
Object Properties	116	10	9	231	331	12	1	604	50	242	232	847
Annotation Properties	192	37	53	257	119	11	19	106	41	284	97	656
Connected Components	7	1	2	1	1	3	1	3	1	2	5	8
Data Quality Check Errors												
Value Errors	1	0	0	0	0	0	0	0	0	0	0	0
Identifier Errors	0	0	0	0	0	0	0	0	0	0	2	2
Deprecated Entities	2	18,506	6,430	304	2,305	0	42	11	341	1,570	0	0
Obsolete Entities	13	0	0	0	0	0	0	1	0	0	0	0
Punning	16	0	0	0	0	0	0	0	0	0	0	8
Consistency[d]	Yes	Yes	Yes	Yes	Yes	Yes	Yes	Yes	Yes	Yes	Yes	---
Semantic Heterogeneity	---	---	---	---	---	---	---	---	---	---	---	7
Identifier Alignment	---	---	---	---	---	---	---	---	---	---	---	23,624
Post-Processed Statistics												
Edges	1,422,153	5,190,485	1,343,218	885,379	2,277,425	2,079,356	34,901	7,873	41,980	734,768	89,764	13,748,009
Classes	111,696	137,592	55,807	38,530	52,937	148,243	2,600	115	2,569	27,170	7,085	545,259
Individuals	33	0	0	0	17	0	0	5	0	0	165	188
Object Properties	112	10	9	231	330	12	1	594	50	238	232	846
Annotation Properties	187	37	53	257	119	11	19	106	41	284	97	656
Connected Components	7	1	2	1	1	3	1	3	1	2	5	8

Note. The OBO Foundry ontologies reported above apply to the PKT Human Disease KG v2.1.0. The extended version of Uberon used in this graph imports the full version of the Cell Ontology.

[a]The numbers for the ontologies are calculated using the versions of the ontologies which include all imported ontologies referenced by the primary ontology. This means that the counts of classes include all Web Ontology classes used for logical definitions, not only those that are explicitly part of the primary ontology's namespace.

[b]The PRO version references the human (NCBITaxon_9606) subset created for the PheKnowLator ecosystem.

[c]Merged represents all of the OBO Foundry ontologies merged into a single ontology.

[d]Consistency was evaluated using the ELK reasoner. The reasoner was only applied to individual OBO Foundry ontologies.

Acronyms: OBO (Open Biological and Biomedical Ontologies); CLO (Cell Line Ontology); ChEBI (Chemical Entities of Biological Interest); GO (Gene Ontology); HPO (Human Phenotype Ontology); Mondo (Mondo Disease Ontology); PRO (Protein Ontology); PW (Pathway Ontology); RO (Relation Ontology); SO (Sequence Ontology); Uberon (Uber-Anatomy Ontology); VO (Vaccine Ontology).

Supplementary Table 14. PheKnowLator Knowledge Modeling Approaches.

Example: Add <<EDNRB, Causes, ABCD syndrome>> to an ontologically-grounded knowledge graph.

Challenge: EDNRB is not currently represented in an ontology. ABCD syndrome is a class in the Human Phenotype Ontology, and is included in the knowledge graph.

Solution: Gene is a class in the Sequence Ontology and can be used to add EDNRB to the knowledge graph using two different strategies.

Instance-based Knowledge Model (ABox)	Class-based Knowledge Model (TBox)
EDNRB, rdfs:subClassOf, Gene<br>EDNRB, rdf:type, owl:Class<br><br>UUID1, rdf:type, EDNRB<br>UUID1, rdf:type, owl:NamedIndividual<br><br>UUID2, rdf:type,ABCD syndrome<br>UUID2, rdf:type, owl:NamedIndividual<br><br>UUID1, Causes, UUID2	EDNRB, rdfs:subClassOf, Gene<br>EDNRB, rdf:type, owl:Class<br><br>UUID1, rdfs:subClassOf, EDNRB<br>UUID1, rdfs:subClassOf, UUID2<br>UUID2, rdf:type, owl:Restriction<br>UUID2, owl:someValuesFrom, ABCD syndrome<br>UUID2, owl:onProperty, Causes

Note. UUID1 and UUID2 are blank nodes or existential variables.[162] Pink highlighting is used for the EDNRB gene instance, yellow highlighting is used for the gene class, and green is used for the ABCD syndrome class.

Acronyms: EDNRB (endothelin receptor type B); OWL (Web Ontology Language); RDF (Resource Description Framework); RDFS (Resource Description Framework Syntax).

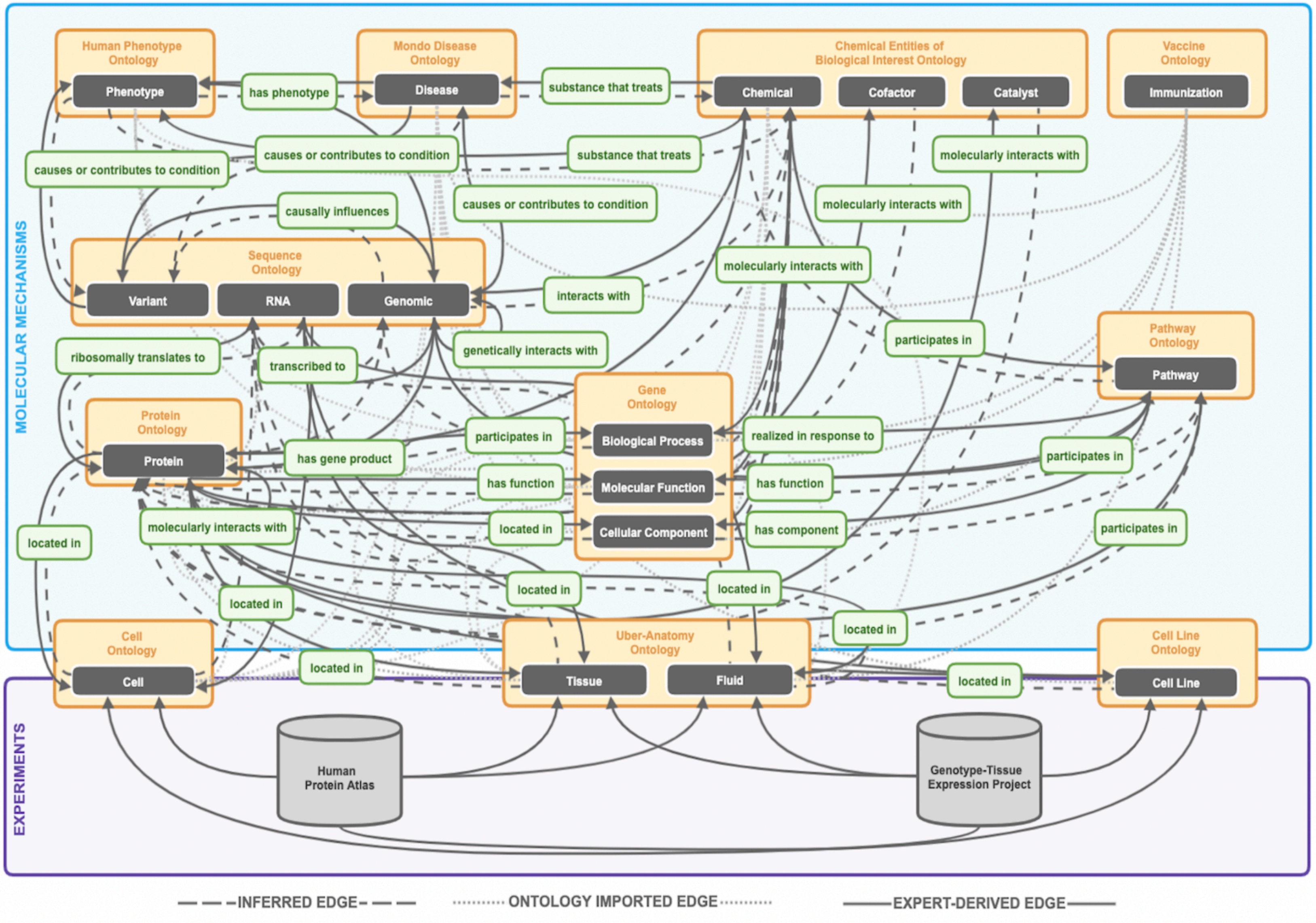

Supplementary Figure 1. Human Disease Mechanism Graph Knowledge Representation.

This figure illustrates the knowledge representation used to construct the human disease mechanisms knowledge graphs. The purple box represents experimental data and the blue box contains the molecular mechanisms created by integrating Open Biological and Biomedical (OBO) Foundry ontologies (gold and green). Edges between the ontologies are created by integrating other data sources that are not part of an OBO Foundry ontology (solid black lines). Dashed lines represent relationships that are inferred from the Relation Ontology and dotted lines represent relationships that exist between imported ontologies.

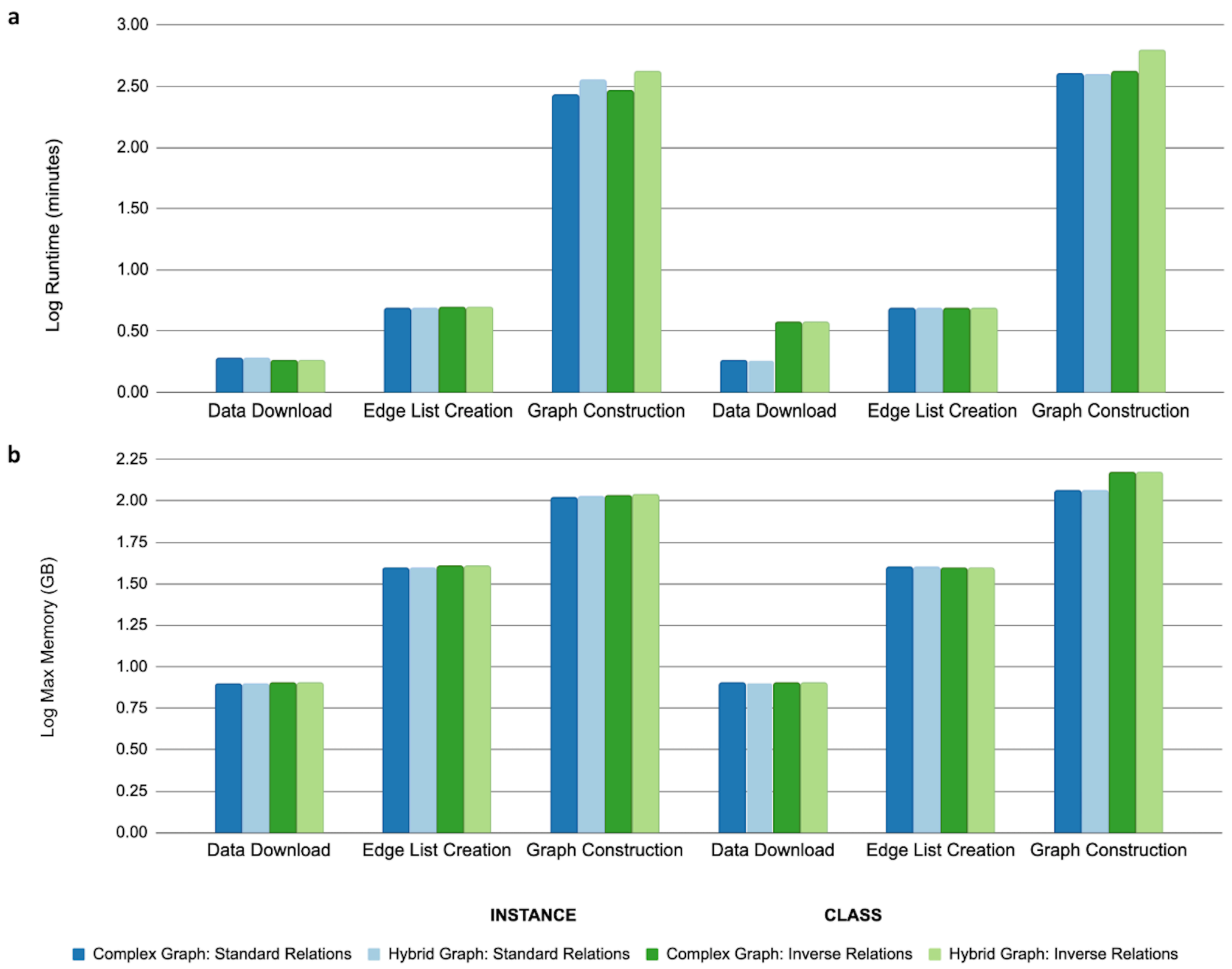

Supplementary Figure 2. PKT Human Disease Knowledge Graph Construction - Computational Performance.

This figure illustrates the (**A**) log runtime and (**B**) log max memory use (GB) performance for each build step with respect to the different build parameterizations or benchmarks provided by the PheKnowLator ecosystem. The ecosystem enables users to fully customize KGs generated by the Graph Construction build step through the following parameters: knowledge model (i.e., complex graphs constructed using class- or instance-based knowledge models), relation strategy (i.e., standard directed relations or inverse bidirectional relations), and semantic abstraction (i.e., transformation of complex graphs into hybrid graphs). The Data Download and Edge List Creation steps are the same regardless of how the Graph Construction step is parameterized. Computational performance was determined using an unreleased build (April 11, 2021) while testing the v.2.1 release

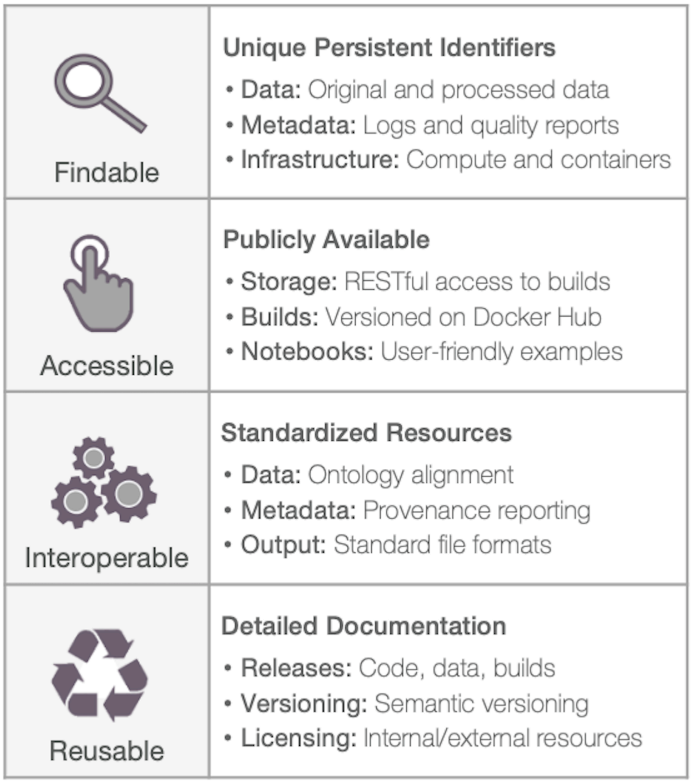

Supplementary Figure 3. The PheKnowLator Ecosystem on FAIR Principles.

The PheKnowLator Ecosystem is built on the FAIR principles of Findability, Accessibility, Interoperability, and Reusability. *Findability.* Use of unique persistent identifiers for all downloaded and processed data, Docker containers, and compute instances and generation of metadata, reports, and logs. *Accessibility.* All resources are accessible via RESTful API access to a dedicated Google Cloud Storage Bucket (pre-2024) or Zenodo Archive (2024), all builds are versioned, and Jupyter Notebooks are used to improve the usability of the Ecosystem resources. ***Interoperability.*** Built on Semantic Web standards, grounded in Open Biological and Biomedical Foundry ontologies, and adoption of standard identifiers for all resources. *Reusability.* Builds are automated, containerized, and deployed through GitHub Actions workflows, resources, scripts, and workflows are versioned using Semantic Versioning, the Ecosystem is licensed, and licensing constraints are enforced for all ingested data.

Supplementary Documents

Knowledge Graph Build Output Metadata

This section provides examples of the metadata files that are output by the PheKnowLator ecosystem for each constructed knowledge graph. All of the example files were pulled from the most recent PKT Human Disease KG build, generated on November 1, 2021. For additional details on this build, please see the associated GitHub wiki: https://github.com/callahantiff/PheKnowLator/wiki/November-01%2C-2021. The logs included in this section are for the class-based + standard relations + OWL-NETS knowledge graph type from this build.

Table of Contents

Supplementary Document 1. downloaded_build_metadata.txt. 24
Supplementary Document 2. preprocessed_build_metadata.txt 29
Supplementary Document 3. edge_source_metadata.txt. 33
Supplementary Document 4. ontology_source_metadata.txt. 40
Supplementary Document 5. ontology_cleaning_report.txt. 43
Supplementary Document 6. pkt_builder_phases12_log.log (Data Download and Preparation). 47
Supplementary Document 7. pkt_build_log.log (Knowledge Graph Construction). 51

Supplementary Document 1. downloaded_build_metadata.txt.

```
==================================
Mon Nov 01 01:45:15 UTC 2021
==================================

DATA INFO
  - DOWNLOAD_URL = http://purl.obolibrary.org/obo/hp.owl
  - DOWNLOAD_DATE = 11/01/2021
  - FILE_SIZE_IN_BYTES = 84212277
GOOGLE_CLOUD_STORAGE_URL= https://storage.googleapis.com/pheknowlator/archived_builds/release_v3.0.2/build_01NOV2021/data/original_data/hp_with_imports.owl

DATA INFO
  - DOWNLOAD_URL = http://purl.obolibrary.org/obo/go.owl
  - DOWNLOAD_DATE = 11/01/2021
  - FILE_SIZE_IN_BYTES = 132081899
  - GOOGLE_CLOUD_STORAGE_URL = https://storage.googleapis.com/pheknowlator/archived_builds/release_v3.0.2/build_01NOV2021/data/original_data/go_with_imports.owl

DATA INFO
  - DOWNLOAD_URL = http://purl.obolibrary.org/obo/mondo.owl
  - DOWNLOAD_DATE = 11/01/2021
  - FILE_SIZE_IN_BYTES = 235314739
  - GOOGLE_CLOUD_STORAGE_URL = https://storage.googleapis.com/pheknowlator/archived_builds/release_v3.0.2/build_01NOV2021/data/original_data/mondo_with_imports.owl

DATA INFO
  - DOWNLOAD_URL = http://purl.obolibrary.org/obo/vo.owl
  - DOWNLOAD_DATE = 11/01/2021
  - FILE_SIZE_IN_BYTES = 8110267
  - GOOGLE_CLOUD_STORAGE_URL = https://storage.googleapis.com/pheknowlator/archived_builds/release_v3.0.2/build_01NOV2021/data/original_data/vo_with_imports.owl

DATA INFO
  - DOWNLOAD_URL = http://purl.obolibrary.org/obo/chebi.owl
  - DOWNLOAD_DATE = 11/01/2021
  - FILE_SIZE_IN_BYTES = 651338784
  - GOOGLE_CLOUD_STORAGE_URL = https://storage.googleapis.com/pheknowlator/archived_builds/release_v3.0.2/build_01NOV2021/data/original_data/chebi_with_imports.owl

DATA INFO
  - DOWNLOAD_URL = http://purl.obolibrary.org/obo/uberon/ext.owl
  - DOWNLOAD_DATE = 11/01/2021
  - FILE_SIZE_IN_BYTES = 65910831
  - GOOGLE_CLOUD_STORAGE_URL = https://storage.googleapis.com/pheknowlator/archived_builds/release_v3.0.2/build_01NOV2021/data/original_data/ext_with_imports.owl

DATA INFO
  - DOWNLOAD_URL = http://purl.obolibrary.org/obo/clo.owl
  - DOWNLOAD_DATE = 11/01/2021
  - FILE_SIZE_IN_BYTES = 119273027
  - GOOGLE_CLOUD_STORAGE_URL = https://storage.googleapis.com/pheknowlator/archived_builds/release_v3.0.2/build_01NOV2021/data/original_data/clo_with_imports.owl

DATA INFO
  - DOWNLOAD_URL = http://purl.obolibrary.org/obo/pr.owl
  - DOWNLOAD_DATE = 11/01/2021
  - FILE_SIZE_IN_BYTES = 1223936557
  - GOOGLE_CLOUD_STORAGE_URL = https://storage.googleapis.com/pheknowlator/archived_builds/release_v3.0.2/build_01NOV2021/data/original_data/pr_with_imports.owl

DATA INFO
  - DOWNLOAD_URL = http://purl.obolibrary.org/obo/so.owl
  - DOWNLOAD_DATE = 11/01/2021
  - FILE_SIZE_IN_BYTES = 5225970
  - GOOGLE_CLOUD_STORAGE_URL = https://storage.googleapis.com/pheknowlator/archived_builds/release_v3.0.2/build_01NOV2021/data/original_data/so_with_imports.owl

DATA INFO
  - DOWNLOAD_URL = http://purl.obolibrary.org/obo/pw.owl
  - DOWNLOAD_DATE = 11/01/2021
  - FILE_SIZE_IN_BYTES = 4965358
```

- GOOGLE_CLOUD_STORAGE_URL = https://storage.googleapis.com/pheknowlator/archived_builds/release_v3.0.2/build_01NOV2021/data/original_data/pw_with_imports.owl

DATA INFO
- DOWNLOAD_URL = http://purl.obolibrary.org/obo/ro.owl
- DOWNLOAD_DATE = 11/01/2021
- FILE_SIZE_IN_BYTES = 867789
- GOOGLE_CLOUD_STORAGE_URL = https://storage.googleapis.com/pheknowlator/archived_builds/release_v3.0.2/build_01NOV2021/data/original_data/ro_with_imports.owl

DATA INFO
- DOWNLOAD_URL = http://ftp.ebi.ac.uk/pub/databases/genenames/hgnc/tsv/hgnc_complete_set.txt
- DOWNLOAD_DATE = 11/01/2021
- FILE_SIZE_IN_BYTES = 15972451
- GOOGLE_CLOUD_STORAGE_URL = https://storage.googleapis.com/pheknowlator/archived_builds/release_v3.0.2/build_01NOV2021/data/original_data/hgnc_complete_set.txt

DATA INFO
- DOWNLOAD_URL = ftp://ftp.ensembl.org/pub/release-102/gtf/homo_sapiens/Homo_sapiens.GRCh38.102.gtf.gz
- DOWNLOAD_DATE = 11/01/2021
- FILE_SIZE_IN_BYTES = 1280942256
- GOOGLE_CLOUD_STORAGE_URL = https://storage.googleapis.com/pheknowlator/archived_builds/release_v3.0.2/build_01NOV2021/data/original_data/Homo_sapiens.GRCh38.102.gtf

DATA INFO
- DOWNLOAD_URL = ftp://ftp.ensembl.org/pub/release-102/tsv/homo_sapiens/Homo_sapiens.GRCh38.102.uniprot.tsv.gz
- DOWNLOAD_DATE = 11/01/2021
- FILE_SIZE_IN_BYTES = 13485382

GOOGLE_CLOUD_STORAGE_URL= https://storage.googleapis.com/pheknowlator/archived_builds/release_v3.0.2/build_01NOV2021/data/original_data/Homo_sapiens.GRCh38.102.uniprot.tsv

DATA INFO
- DOWNLOAD_URL = ftp://ftp.ensembl.org/pub/release-102/tsv/homo_sapiens/Homo_sapiens.GRCh38.102.entrez.tsv.gz
- DOWNLOAD_DATE = 11/01/2021
- FILE_SIZE_IN_BYTES = 17055479

GOOGLE_CLOUD_STORAGE_URL= https://storage.googleapis.com/pheknowlator/archived_builds/release_v3.0.2/build_01NOV2021/data/original_data/Homo_sapiens.GRCh38.102.entrez.tsv

DATA INFO
DOWNLOAD_URL=
https://www.uniprot.org/uniprot/?query=&fil=organism%3A%22Homo%20sapiens%20(Human)%20%5B9606%5D%22&columns=id%2Creviewed%2Cdatabase(GeneID)%2Cdatabase(Ensembl)%2Cdatabase(HGNC)%2Cgenes(ALTERNATIVE)%2Cgenes(PREFERRED)&format=tab
- DOWNLOAD_DATE = 11/01/2021
- FILE_SIZE_IN_BYTES = 8759737
- GOOGLE_CLOUD_STORAGE_URL = https://storage.googleapis.com/pheknowlator/archived_builds/release_v3.0.2/build_01NOV2021/data/original_data/uniprot_identifier_mapping.tab

DATA INFO
- DOWNLOAD_URL = ftp://ftp.ncbi.nih.gov/gene/DATA/GENE_INFO/Mammalia/Homo_sapiens.gene_info.gz
- DOWNLOAD_DATE = 11/01/2021
- FILE_SIZE_IN_BYTES = 13909469
- GOOGLE_CLOUD_STORAGE_URL = https://storage.googleapis.com/pheknowlator/archived_builds/release_v3.0.2/build_01NOV2021/data/original_data/Homo_sapiens.gene_info

DATA INFO
- DOWNLOAD_URL = https://proconsortium.org/download/current/promapping.txt
- DOWNLOAD_DATE = 11/01/2021
- FILE_SIZE_IN_BYTES = 15271039
- GOOGLE_CLOUD_STORAGE_URL = https://storage.googleapis.com/pheknowlator/archived_builds/release_v3.0.2/build_01NOV2021/data/original_data/promapping.txt

DATA INFO
- DOWNLOAD_URL = ftp://nlmpubs.nlm.nih.gov/online/mesh/rdf/2021/mesh2021.nt
- DOWNLOAD_DATE = 11/01/2021
- FILE_SIZE_IN_BYTES = 1948657299
- GOOGLE_CLOUD_STORAGE_URL = https://storage.googleapis.com/pheknowlator/archived_builds/release_v3.0.2/build_01NOV2021/data/original_data/mesh2021.nt

DATA INFO
- DOWNLOAD_URL = ftp://ftp.ebi.ac.uk/pub/databases/chebi/Flat_file_tab_delimited/names.tsv.gz
- DOWNLOAD_DATE = 11/01/2021
- FILE_SIZE_IN_BYTES = 30365252
- GOOGLE_CLOUD_STORAGE_URL = https://storage.googleapis.com/pheknowlator/archived_builds/release_v3.0.2/build_01NOV2021/data/original_data/names.tsv

DATA INFO
- DOWNLOAD_URL = https://www.disgenet.org/static/disgenet_ap1/files/downloads/disease_mappings.tsv.gz
- DOWNLOAD_DATE = 11/01/2021

- FILE_SIZE_IN_BYTES = 18367848
- GOOGLE_CLOUD_STORAGE_URL = https://storage.googleapis.com/pheknowlator/archived_builds/release_v3.0.2/build_01NOV2021/data/original_data/disease_mappings.tsv

DATA INFO
DOWNLOAD_URL=
https://www.proteinatlas.org/api/search_download.php?search=&columns=g,eg,up,pe,rnatsm,rnaclsm,rnacasm,rnabrsm,rnabcsm,rnablsm,scl,t_RNA_adipose_tissue,t_RNA_adrenal_gland,t_RNA_amygdala,t_RNA_appendix,t_RNA_basal_ganglia,t_RNA_
bone_marrow,t_RNA_breast,t_RNA_cerebellum,t_RNA_cerebral_cortex,t_RNA_cervix,_uterine,t_RNA_colon,t_RNA_corpus_callosum,t_RNA_ductus_deferens,t_RNA_duodenum,t_RNA_endometrium_1,t_RNA_epididymis,t_RNA_esophagus,t_RNA_fallo
pian_tube,t_RNA_gallbladder,t_RNA_heart_muscle,t_RNA_hippocampal_formation,t_RNA_hypothalamus,t_RNA_kidney,t_RNA_liver,t_RNA_lung,t_RNA_lymph_node,t_RNA_midbrain,t_RNA_olfactory_region,t_RNA_ovary,t_RNA_pancreas,t_RNA_parath
yroid_gland,t_RNA_pituitary_gland,t_RNA_placenta,t_RNA_pons_and_medulla,t_RNA_prostate,t_RNA_rectum,t_RNA_retina,t_RNA_salivary_gland,t_RNA_seminal_vesicle,t_RNA_skeletal_muscle,t_RNA_skin_1,t_RNA_small_intestine,t_RNA_smooth_m
uscle,t_RNA_spinal_cord,t_RNA_spleen,t_RNA_stomach_1,t_RNA_testis,t_RNA_thalamus,t_RNA_thymus,t_RNA_thyroid_gland,t_RNA_tongue,t_RNA_tonsil,t_RNA_urinary_bladder,t_RNA_vagina,t_RNA_B-cells,t_RNA_dendritic_cells,t_RNA_granulocytes
,t_RNA_monocytes,t_RNA_NK-cells,t_RNA_T-cells,t_RNA_total_PBMC,cell_RNA_A-431,cell_RNA_A549,cell_RNA_AF22,cell_RNA_AN3-CA,cell_RNA_ASC_diff,cell_RNA_ASC_TERT1,cell_RNA_BEWO,cell_RNA_BJ,cell_RNA_BJ_hTERT+,cell_RNA_BJ_hTERT+_
SV40_Large_T+,cell_RNA_BJ_hTERT+_SV40_Large_T+_RasG12V,cell_RNA_CACO-2,cell_RNA_CAPAN-2,cell_RNA_Daudi,cell_RNA_EFO-21,cell_RNA_fHDF/TERT166,cell_RNA_HaCaT,cell_RNA_HAP1,cell_RNA_HBEC3-KT,cell_RNA_HBF_TERT88,cell_RNA_HD
LM-2,cell_RNA_HEK_293,cell_RNA_HEL,cell_RNA_HeLa,cell_RNA_Hep_G2,cell_RNA_HHSteC,cell_RNA_HL-60,cell_RNA_HMC-1,cell_RNA_HSkMC,cell_RNA_hTCEpi,cell_RNA_hTEC/SVTERT24-B,cell_RNA_hTERT-HME1,cell_RNA_HUVEC_TERT2,cell_RNA_K-
562,cell_RNA_Karpas-707,cell_RNA_LHCN-M2,cell_RNA_MCF7,cell_RNA_MOLT-4,cell_RNA_NB-4,cell_RNA_NTERA-2,cell_RNA_PC-3,cell_RNA_REH,cell_RNA_RH-30,cell_RNA_RPMI-8226,cell_RNA_RPTEC_TERT1,cell_RNA_RT4,cell_RNA_SCLC-21H,cell_RN
A_SH-SY5Y,cell_RNA_SiHa,cell_RNA_SK-BR-3,cell_RNA_SK-MEL-30,cell_RNA_T-47d,cell_RNA_THP-1,cell_RNA_TIME,cell_RNA_U-138_MG,cell_RNA_U-2_OS,cell_RNA_U-2197,cell_RNA_U-251_MG,cell_RNA_U-266/70,cell_RNA_U-266/84,cell_RNA_U-698,
cell_RNA_U-87_MG,cell_RNA_U-937,cell_RNA_WM-115,blood_RNA_basophil,blood_RNA_classical_monocyte,blood_RNA_eosinophil,blood_RNA_gdT-cell,blood_RNA_intermediate_monocyte,blood_RNA_MAIT_T-cell,blood_RNA_memory_B-cell,blood_
RNA_memory_CD4_T-cell,blood_RNA_memory_CD8_T-cell,blood_RNA_myeloid_DC,blood_RNA_naive_B-cell,blood_RNA_naive_CD4_T-cell,blood_RNA_naive_CD8_T-cell,blood_RNA_neutrophil,blood_RNA_NK-cell,blood_RNA_non-classical_monocyte,bl
ood_RNA_plasmacytoid_DC,blood_RNA_T-reg,blood_RNA_total_PBMC,brain_RNA_amygdala,brain_RNA_basal_ganglia,brain_RNA_cerebellum,brain_RNA_cerebral_cortex,brain_RNA_hippocampal_formation,brain_RNA_hypothalamus,brain_RNA_midbr
ain,brain_RNA_olfactory_region,brain_RNA_pons_and_medulla,brain_RNA_thalamus&format=tsv
- DOWNLOAD_DATE = 11/01/2021
- FILE_SIZE_IN_BYTES = 15328159
- GOOGLE_CLOUD_STORAGE_URL = https://storage.googleapis.com/pheknowlator/archived_builds/release_v3.0.2/build_01NOV2021/data/original_data/proteinatlas_search.tsv

DATA INFO
- DOWNLOAD_URL = https://storage.googleapis.com/gtex_analysis_v8/rna_seq_data/GTEx_Analysis_2017-06-05_v8_RNASeQCv1.1.9_gene_median_tpm.gct.gz
- DOWNLOAD_DATE = 11/01/2021
- FILE_SIZE_IN_BYTES = 17780477

GOOGLE_CLOUD_STORAGE_URL= https://storage.googleapis.com/pheknowlator/archived_builds/release_v3.0.2/build_01NOV2021/data/original_data/GTEx_Analysis_2017-06-05_v8_RNASeQCv1.1.9_gene_median_tpm.gct

DATA INFO
- DOWNLOAD_URL = https://reactome.org/download/current/ReactomePathways.txt
- DOWNLOAD_DATE = 11/01/2021
- FILE_SIZE_IN_BYTES = 1423494
- GOOGLE_CLOUD_STORAGE_URL = https://storage.googleapis.com/pheknowlator/archived_builds/release_v3.0.2/build_01NOV2021/data/original_data/ReactomePathways.txt

DATA INFO
- DOWNLOAD_URL = https://reactome.org/download/current/gene_association.reactome.gz
- DOWNLOAD_DATE = 11/01/2021
- FILE_SIZE_IN_BYTES = 11955743
- GOOGLE_CLOUD_STORAGE_URL = https://storage.googleapis.com/pheknowlator/archived_builds/release_v3.0.2/build_01NOV2021/data/original_data/gene_association.reactome

DATA INFO
- DOWNLOAD_URL = https://reactome.org/download/current/ChEBI2Reactome_All_Levels.txt
- DOWNLOAD_DATE = 11/01/2021
- FILE_SIZE_IN_BYTES = 30485396
- GOOGLE_CLOUD_STORAGE_URL = https://storage.googleapis.com/pheknowlator/archived_builds/release_v3.0.2/build_01NOV2021/data/original_data/ChEBI2Reactome_All_Levels.txt

DATA INFO
- DOWNLOAD_URL = http://compath.scai.fraunhofer.de/export_mappings
- DOWNLOAD_DATE = 11/01/2021
- FILE_SIZE_IN_BYTES = 196388

GOOGLE_CLOUD_STORAGE_URL= https://storage.googleapis.com/pheknowlator/archived_builds/release_v3.0.2/build_01NOV2021/data/original_data/compath_canonical_pathway_mappings.txt

DATA INFO
- DOWNLOAD_URL = https://raw.githubusercontent.com/ComPath/resources/master/mappings/kegg_reactome.csv
- DOWNLOAD_DATE = 11/01/2021
- FILE_SIZE_IN_BYTES = 92309
- GOOGLE_CLOUD_STORAGE_URL = https://storage.googleapis.com/pheknowlator/archived_builds/release_v3.0.2/build_01NOV2021/data/original_data/kegg_reactome.csv

DATA INFO
- DOWNLOAD_URL = https://storage.googleapis.com/pheknowlator/curated_data/genomic_sequence_ontology_mappings.xlsx
- DOWNLOAD_DATE = 11/01/2021
- FILE_SIZE_IN_BYTES = 20641

GOOGLE_CLOUD_STORAGE_URL= https://storage.googleapis.com/pheknowlator/archived_builds/release_v3.0.2/build_01NOV2021/data/original_data/genomic_sequence_ontology_mappings.xlsx

DATA INFO

DOWNLOAD_URL=
https://sparql.proconsortium.org/virtuoso/sparql?query=PREFIX+obo%3A+%3Chttp%3A%2F%2Fpurl.obolibrary.org%2Fobo%2F%3E%0D%0A%0D%0ASELECT+%3FPRO_term%0D%0AFROM+%3Chttp%3A%2F%2Fpurl.obolibrary.org%2Fobo%2Fpr%3E%0D%
0AWHERE+%7B%0D%0A+++++++%3FPRO_term+rdf%3Atype+owl%3AClass+.%0D%0A+++++++%3FPRO_term+rdfs%3AsubClassOf+%3Frestriction+.%0D%0A+++++++%3Frestriction+owl%3AonProperty+obo%3ARO_0002160+.%0D%0A+++++++%3Frestricti
on+owl%3AsomeValuesFrom+obo%3ANCBITaxon_9606+.%0D%0A%0D%0A+++++++%23+use+this+to+filter-out+things+like+hgnc+ids%0D%0A+++++++FILTER+%28regex%28%3FPRO_term%2C%22http%3A%2F%2Fpurl.obolibrary.org%2Fobo%2F*%22%29
%29+.%0D%0A%7D&format=text%2Fhtml&debug=
- DOWNLOAD_DATE = 11/01/2021
- FILE_SIZE_IN_BYTES = 1301002
- GOOGLE_CLOUD_STORAGE_URL = https://storage.googleapis.com/pheknowlator/archived_builds/release_v3.0.2/build_01NOV2021/data/original_data/human_pro_classes.html

DATA INFO
- DOWNLOAD_URL = ftp://ftp.ncbi.nlm.nih.gov/pub/clinvar/tab_delimited/variant_summary.txt.gz
- DOWNLOAD_DATE = 11/01/2021
- FILE_SIZE_IN_BYTES = 954216609
- GOOGLE_CLOUD_STORAGE_URL = https://storage.googleapis.com/pheknowlator/archived_builds/release_v3.0.2/build_01NOV2021/data/original_data/variant_summary.txt

DATA INFO
DOWNLOAD_URL=
https://www.uniprot.org/uniprot/?query=&fil=organism%3A%22Homo%20sapiens%20(Human)%20%5B9606%5D%22&columns=id%2Creviewed%2Centry%20name%2Cdatabase(PRO)%2Cchebi(Cofactor)%2Cchebi(Catalytic%20activity)&format=tab
- DOWNLOAD_DATE = 11/01/2021
- FILE_SIZE_IN_BYTES = 10491909
- GOOGLE_CLOUD_STORAGE_URL = https://storage.googleapis.com/pheknowlator/archived_builds/release_v3.0.2/build_01NOV2021/data/original_data/uniprot-cofactor-catalyst.tab

DATA INFO
- DOWNLOAD_URL = http://ctdbase.org/reports/CTD_chem_gene_ixns.tsv.gz
- DOWNLOAD_DATE = 11/01/2021
- FILE_SIZE_IN_BYTES = 441097103
- GOOGLE_CLOUD_STORAGE_URL = https://storage.googleapis.com/pheknowlator/archived_builds/release_v3.0.2/build_01NOV2021/data/original_data/CTD_chem_gene_ixns.tsv

DATA INFO
- DOWNLOAD_URL = http://ctdbase.org/reports/CTD_chem_go_enriched.tsv.gz
- DOWNLOAD_DATE = 11/01/2021
- FILE_SIZE_IN_BYTES = 817733569
- GOOGLE_CLOUD_STORAGE_URL = https://storage.googleapis.com/pheknowlator/archived_builds/release_v3.0.2/build_01NOV2021/data/original_data/CTD_chem_go_enriched.tsv

DATA INFO
- DOWNLOAD_URL = https://reactome.org/download/current/ChEBI2Reactome_All_Levels.txt
- DOWNLOAD_DATE = 11/01/2021
- FILE_SIZE_IN_BYTES = 30485396
- GOOGLE_CLOUD_STORAGE_URL = https://storage.googleapis.com/pheknowlator/archived_builds/release_v3.0.2/build_01NOV2021/data/original_data/ChEBI2Reactome_All_Levels.txt

DATA INFO
- DOWNLOAD_URL = http://ctdbase.org/reports/CTD_chemicals_diseases.tsv.gz
- DOWNLOAD_DATE = 11/01/2021
- FILE_SIZE_IN_BYTES = 701201197
- GOOGLE_CLOUD_STORAGE_URL = https://storage.googleapis.com/pheknowlator/archived_builds/release_v3.0.2/build_01NOV2021/data/original_data/CTD_chemicals_diseases.tsv

DATA INFO
- DOWNLOAD_URL = http://purl.obolibrary.org/obo/hp/hpoa/phenotype.hpoa
- DOWNLOAD_DATE = 11/01/2021
- FILE_SIZE_IN_BYTES = 27315334
- GOOGLE_CLOUD_STORAGE_URL = https://storage.googleapis.com/pheknowlator/archived_builds/release_v3.0.2/build_01NOV2021/data/original_data/phenotype.hpoa

DATA INFO
- DOWNLOAD_URL = https://www.disgenet.org/static/disgenet_ap1/files/downloads/curated_gene_disease_associations.tsv.gz
- DOWNLOAD_DATE = 11/01/2021
- FILE_SIZE_IN_BYTES = 11542996

GOOGLE_CLOUD_STORAGE_URL= https://storage.googleapis.com/pheknowlator/archived_builds/release_v3.0.2/build_01NOV2021/data/original_data/curated_gene_disease_associations.tsv

DATA INFO
- DOWNLOAD_URL = http://genemania.org/data/current/Homo_sapiens.COMBINED/COMBINED.DEFAULT_NETWORKS.BP_COMBINING.txt
- DOWNLOAD_DATE = 11/01/2021
- FILE_SIZE_IN_BYTES = 246190113

GOOGLE_CLOUD_STORAGE_URL= https://storage.googleapis.com/pheknowlator/archived_builds/release_v3.0.2/build_01NOV2021/data/original_data/COMBINED.DEFAULT_NETWORKS.BP_COMBINING.txt

DATA INFO
- DOWNLOAD_URL = http://ctdbase.org/reports/CTD_genes_pathways.tsv.gz

- DOWNLOAD_DATE = 11/01/2021
- FILE_SIZE_IN_BYTES = 8192661
- GOOGLE_CLOUD_STORAGE_URL = https://storage.googleapis.com/pheknowlator/archived_builds/release_v3.0.2/build_01NOV2021/data/original_data/CTD_genes_pathways.tsv

DATA INFO
- DOWNLOAD_URL = https://reactome.org/download/current/gene_association.reactome.gz
- DOWNLOAD_DATE = 11/01/2021
- FILE_SIZE_IN_BYTES = 11955743
- GOOGLE_CLOUD_STORAGE_URL = https://storage.googleapis.com/pheknowlator/archived_builds/release_v3.0.2/build_01NOV2021/data/original_data/gene_association.reactome

DATA INFO
- DOWNLOAD_URL = http://current.geneontology.org/annotations/goa_human.gaf.gz
- DOWNLOAD_DATE = 11/01/2021
- FILE_SIZE_IN_BYTES = 109325311
- GOOGLE_CLOUD_STORAGE_URL = https://storage.googleapis.com/pheknowlator/archived_builds/release_v3.0.2/build_01NOV2021/data/original_data/goa_human.gaf

DATA INFO
- DOWNLOAD_URL = https://reactome.org/download/current/UniProt2Reactome_All_Levels.txt
- DOWNLOAD_DATE = 11/01/2021
- FILE_SIZE_IN_BYTES = 102191227
- GOOGLE_CLOUD_STORAGE_URL = https://storage.googleapis.com/pheknowlator/archived_builds/release_v3.0.2/build_01NOV2021/data/original_data/UniProt2Reactome_All_Levels.txt

DATA INFO
- DOWNLOAD_URL = https://stringdb-static.org/download/protein.links.v11.0/9606.protein.links.v11.0.txt.gz
- DOWNLOAD_DATE = 11/01/2021
- FILE_SIZE_IN_BYTES = 540934917
- GOOGLE_CLOUD_STORAGE_URL = https://storage.googleapis.com/pheknowlator/archived_builds/release_v3.0.2/build_01NOV2021/data/original_data/9606.protein.links.v11.0.txt

DATA INFO
- DOWNLOAD_URL = https://storage.googleapis.com/pheknowlator/curated_data/genomic_typing_dict.pkl
- DOWNLOAD_DATE = 11/01/2021
- FILE_SIZE_IN_BYTES = 2268
- GOOGLE_CLOUD_STORAGE_URL = https://storage.googleapis.com/pheknowlator/archived_builds/release_v3.0.2/build_01NOV2021/data/original_data/genomic_typing_dict.pkl

DATA INFO
- DOWNLOAD_URL = https://storage.googleapis.com/pheknowlator/curated_data/zooma_tissue_cell_mapping_04JAN2020.xlsx
- DOWNLOAD_DATE = 11/01/2021
- FILE_SIZE_IN_BYTES = 18225305

GOOGLE_CLOUD_STORAGE_URL= https://storage.googleapis.com/pheknowlator/archived_builds/release_v3.0.2/build_01NOV2021/data/original_data/zooma_tissue_cell_mapping_04JAN2020.xlsx

Supplementary Document 2. preprocessed_build_metadata.txt

```
==================================
Mon Nov 01 10:00:03 UTC 2021
==================================

DATA INFO
DOWNLOAD_URL= https://storage.googleapis.com/pheknowlator/archived_builds/release_v3.0.2/build_01NOV2021/data/original_data/CLINVAR_VARIANT_GENE_DISEASE_PHENOTYPE_EDGES.txt
  - DOWNLOAD_DATE = 11/01/2021
  - FILE_SIZE_IN_BYTES = 3662353455
GOOGLE_CLOUD_STORAGE_URL = https://storage.googleapis.com/pheknowlator/archived_builds/release_v3.0.2/build_01NOV2021/data/processed_data/CLINVAR_VARIANT_GENE_DISEASE_PHENOTYPE_EDGES.txt

DATA INFO
  - DOWNLOAD_URL = https://storage.googleapis.com/pheknowlator/archived_builds/release_v3.0.2/build_01NOV2021/data/original_data/DISEASE_MONDO_MAP.txt
  - DOWNLOAD_DATE = 11/01/2021
  - FILE_SIZE_IN_BYTES = 3871799
  - GOOGLE_CLOUD_STORAGE_URL = https://storage.googleapis.com/pheknowlator/archived_builds/release_v3.0.2/build_01NOV2021/data/processed_data/DISEASE_MONDO_MAP.txt

DATA INFO
  - DOWNLOAD_URL = https://storage.googleapis.com/pheknowlator/archived_builds/release_v3.0.2/build_01NOV2021/data/original_data/ENSEMBL_GENE_ENTREZ_GENE_MAP.txt
  - DOWNLOAD_DATE = 11/01/2021
  - FILE_SIZE_IN_BYTES = 3564948
  - GOOGLE_CLOUD_STORAGE_URL = https://storage.googleapis.com/pheknowlator/archived_builds/release_v3.0.2/build_01NOV2021/data/processed_data/ENSEMBL_GENE_ENTREZ_GENE_MAP.txt

DATA INFO
  - DOWNLOAD_URL = https://storage.googleapis.com/pheknowlator/archived_builds/release_v3.0.2/build_01NOV2021/data/original_data/ENSEMBL_TRANSCRIPT_PROTEIN_ONTOLOGY_MAP.txt
  - DOWNLOAD_DATE = 11/01/2021
  - FILE_SIZE_IN_BYTES = 2931638
  - GOOGLE_CLOUD_STORAGE_URL = https://storage.googleapis.com/pheknowlator/archived_builds/release_v3.0.2/build_01NOV2021/data/processed_data/ENSEMBL_TRANSCRIPT_PROTEIN_ONTOLOGY_MAP.txt

DATA INFO
  - DOWNLOAD_URL = https://storage.googleapis.com/pheknowlator/archived_builds/release_v3.0.2/build_01NOV2021/data/original_data/ENTREZ_GENE_ENSEMBL_TRANSCRIPT_MAP.txt
  - DOWNLOAD_DATE = 11/01/2021
  - FILE_SIZE_IN_BYTES = 15146432
  - GOOGLE_CLOUD_STORAGE_URL = https://storage.googleapis.com/pheknowlator/archived_builds/release_v3.0.2/build_01NOV2021/data/processed_data/ENTREZ_GENE_ENSEMBL_TRANSCRIPT_MAP.txt

DATA INFO
  - DOWNLOAD_URL = https://storage.googleapis.com/pheknowlator/archived_builds/release_v3.0.2/build_01NOV2021/data/original_data/ENTREZ_GENE_PRO_ONTOLOGY_MAP.txt
  - DOWNLOAD_DATE = 11/01/2021
  - FILE_SIZE_IN_BYTES = 1124944
  - GOOGLE_CLOUD_STORAGE_URL = https://storage.googleapis.com/pheknowlator/archived_builds/release_v3.0.2/build_01NOV2021/data/processed_data/ENTREZ_GENE_PRO_ONTOLOGY_MAP.txt

DATA INFO
  - DOWNLOAD_URL = https://storage.googleapis.com/pheknowlator/archived_builds/release_v3.0.2/build_01NOV2021/data/original_data/GENE_SYMBOL_ENSEMBL_TRANSCRIPT_MAP.txt
  - DOWNLOAD_DATE = 11/01/2021
  - FILE_SIZE_IN_BYTES = 19365937
  - GOOGLE_CLOUD_STORAGE_URL = https://storage.googleapis.com/pheknowlator/archived_builds/release_v3.0.2/build_01NOV2021/data/processed_data/GENE_SYMBOL_ENSEMBL_TRANSCRIPT_MAP.txt

DATA INFO
  - DOWNLOAD_URL = https://storage.googleapis.com/pheknowlator/archived_builds/release_v3.0.2/build_01NOV2021/data/original_data/HPA_GTEX_RNA_GENE_PROTEIN_EDGES.txt
  - DOWNLOAD_DATE = 11/01/2021
  - FILE_SIZE_IN_BYTES = 20611429
  - GOOGLE_CLOUD_STORAGE_URL = https://storage.googleapis.com/pheknowlator/archived_builds/release_v3.0.2/build_01NOV2021/data/processed_data/HPA_GTEX_RNA_GENE_PROTEIN_EDGES.txt

DATA INFO
  - DOWNLOAD_URL = https://storage.googleapis.com/pheknowlator/archived_builds/release_v3.0.2/build_01NOV2021/data/original_data/HPA_GTEx_TISSUE_CELL_MAP.txt
  - DOWNLOAD_DATE = 11/01/2021
  - FILE_SIZE_IN_BYTES = 8222
  - GOOGLE_CLOUD_STORAGE_URL = https://storage.googleapis.com/pheknowlator/archived_builds/release_v3.0.2/build_01NOV2021/data/processed_data/HPA_GTEx_TISSUE_CELL_MAP.txt

DATA INFO
  - DOWNLOAD_URL = https://storage.googleapis.com/pheknowlator/archived_builds/release_v3.0.2/build_01NOV2021/data/original_data/HPA_tissues.txt
  - DOWNLOAD_DATE = 11/01/2021
  - FILE_SIZE_IN_BYTES = 1530
  - GOOGLE_CLOUD_STORAGE_URL = https://storage.googleapis.com/pheknowlator/archived_builds/release_v3.0.2/build_01NOV2021/data/processed_data/HPA_tissues.txt
```

DATA INFO
- DOWNLOAD_URL = https://storage.googleapis.com/pheknowlator/archived_builds/release_v3.0.2/build_01NOV2021/data/original_data/INVERSE_RELATIONS.txt
- DOWNLOAD_DATE = 11/01/2021
- FILE_SIZE_IN_BYTES = 4602
- GOOGLE_CLOUD_STORAGE_URL = https://storage.googleapis.com/pheknowlator/archived_builds/release_v3.0.2/build_01NOV2021/data/processed_data/INVERSE_RELATIONS.txt

DATA INFO
- DOWNLOAD_URL = https://storage.googleapis.com/pheknowlator/archived_builds/release_v3.0.2/build_01NOV2021/data/original_data/MESH_CHEBI_MAP.txt
- DOWNLOAD_DATE = 11/01/2021
- FILE_SIZE_IN_BYTES = 358052
- GOOGLE_CLOUD_STORAGE_URL = https://storage.googleapis.com/pheknowlator/archived_builds/release_v3.0.2/build_01NOV2021/data/processed_data/MESH_CHEBI_MAP.txt

DATA INFO
- DOWNLOAD_URL = https://storage.googleapis.com/pheknowlator/archived_builds/release_v3.0.2/build_01NOV2021/data/original_data/Merged_gene_rna_protein_identifiers.pkl
- DOWNLOAD_DATE = 11/01/2021
- FILE_SIZE_IN_BYTES = 539048900
- GOOGLE_CLOUD_STORAGE_URL = https://storage.googleapis.com/pheknowlator/archived_builds/release_v3.0.2/build_01NOV2021/data/processed_data/Merged_gene_rna_protein_identifiers.pkl

DATA INFO
- DOWNLOAD_URL = https://storage.googleapis.com/pheknowlator/archived_builds/release_v3.0.2/build_01NOV2021/data/original_data/PHENOTYPE_HPO_MAP.txt
- DOWNLOAD_DATE = 11/01/2021
- FILE_SIZE_IN_BYTES = 719692
- GOOGLE_CLOUD_STORAGE_URL = https://storage.googleapis.com/pheknowlator/archived_builds/release_v3.0.2/build_01NOV2021/data/processed_data/PHENOTYPE_HPO_MAP.txt

DATA INFO
- DOWNLOAD_URL = https://storage.googleapis.com/pheknowlator/archived_builds/release_v3.0.2/build_01NOV2021/data/original_data/PheKnowLator_MergedOntologies.owl
- DOWNLOAD_DATE = 11/01/2021
- FILE_SIZE_IN_BYTES = 1450356910
- GOOGLE_CLOUD_STORAGE_URL = https://storage.googleapis.com/pheknowlator/archived_builds/release_v3.0.2/build_01NOV2021/data/processed_data/PheKnowLator_MergedOntologies.owl

DATA INFO
- DOWNLOAD_URL = https://storage.googleapis.com/pheknowlator/archived_builds/release_v3.0.2/build_01NOV2021/data/original_data/REACTOME_PW_GO_MAPPINGS.txt
- DOWNLOAD_DATE = 11/01/2021
- FILE_SIZE_IN_BYTES = 398373
- GOOGLE_CLOUD_STORAGE_URL = https://storage.googleapis.com/pheknowlator/archived_builds/release_v3.0.2/build_01NOV2021/data/processed_data/REACTOME_PW_GO_MAPPINGS.txt

DATA INFO
- DOWNLOAD_URL = https://storage.googleapis.com/pheknowlator/archived_builds/release_v3.0.2/build_01NOV2021/data/original_data/RELATIONS_LABELS.txt
- DOWNLOAD_DATE = 11/01/2021
- FILE_SIZE_IN_BYTES = 45000
- GOOGLE_CLOUD_STORAGE_URL = https://storage.googleapis.com/pheknowlator/archived_builds/release_v3.0.2/build_01NOV2021/data/processed_data/RELATIONS_LABELS.txt

DATA INFO
- DOWNLOAD_URL = https://storage.googleapis.com/pheknowlator/archived_builds/release_v3.0.2/build_01NOV2021/data/original_data/SO_GENE_TRANSCRIPT_VARIANT_TYPE_MAPPING.txt
- DOWNLOAD_DATE = 11/01/2021
- FILE_SIZE_IN_BYTES = 28069897
- GOOGLE_CLOUD_STORAGE_URL = https://storage.googleapis.com/pheknowlator/archived_builds/release_v3.0.2/build_01NOV2021/data/processed_data/SO_GENE_TRANSCRIPT_VARIANT_TYPE_MAPPING.txt

DATA INFO
- DOWNLOAD_URL = https://storage.googleapis.com/pheknowlator/archived_builds/release_v3.0.2/build_01NOV2021/data/original_data/STRING_PRO_ONTOLOGY_MAP.txt
- DOWNLOAD_DATE = 11/01/2021
- FILE_SIZE_IN_BYTES = 1326141
- GOOGLE_CLOUD_STORAGE_URL = https://storage.googleapis.com/pheknowlator/archived_builds/release_v3.0.2/build_01NOV2021/data/processed_data/STRING_PRO_ONTOLOGY_MAP.txt

DATA INFO
- DOWNLOAD_URL = https://storage.googleapis.com/pheknowlator/archived_builds/release_v3.0.2/build_01NOV2021/data/original_data/UNIPROT_ACCESSION_PRO_ONTOLOGY_MAP.txt
- DOWNLOAD_DATE = 11/01/2021
- FILE_SIZE_IN_BYTES = 3736130
- GOOGLE_CLOUD_STORAGE_URL = https://storage.googleapis.com/pheknowlator/archived_builds/release_v3.0.2/build_01NOV2021/data/processed_data/UNIPROT_ACCESSION_PRO_ONTOLOGY_MAP.txt

DATA INFO
- DOWNLOAD_URL = https://storage.googleapis.com/pheknowlator/archived_builds/release_v3.0.2/build_01NOV2021/data/original_data/UNIPROT_PROTEIN_CATALYST.txt
- DOWNLOAD_DATE = 11/01/2021
- FILE_SIZE_IN_BYTES = 1639479
- GOOGLE_CLOUD_STORAGE_URL = https://storage.googleapis.com/pheknowlator/archived_builds/release_v3.0.2/build_01NOV2021/data/processed_data/UNIPROT_PROTEIN_CATALYST.txt

DATA INFO

- DOWNLOAD_URL = https://storage.googleapis.com/pheknowlator/archived_builds/release_v3.0.2/build_01NOV2021/data/original_data/UNIPROT_PROTEIN_COFACTOR.txt
- DOWNLOAD_DATE = 11/01/2021
- FILE_SIZE_IN_BYTES = 179314
- GOOGLE_CLOUD_STORAGE_URL = https://storage.googleapis.com/pheknowlator/archived_builds/release_v3.0.2/build_01NOV2021/data/processed_data/UNIPROT_PROTEIN_COFACTOR.txt

DATA INFO
- DOWNLOAD_URL = https://storage.googleapis.com/pheknowlator/archived_builds/release_v3.0.2/build_01NOV2021/data/original_data/chebi_with_imports.owl
- DOWNLOAD_DATE = 11/01/2021
- FILE_SIZE_IN_BYTES = 643473711
- GOOGLE_CLOUD_STORAGE_URL = https://storage.googleapis.com/pheknowlator/archived_builds/release_v3.0.2/build_01NOV2021/data/processed_data/chebi_with_imports.owl

DATA INFO
- DOWNLOAD_URL = https://storage.googleapis.com/pheknowlator/archived_builds/release_v3.0.2/build_01NOV2021/data/original_data/clo_with_imports.owl
- DOWNLOAD_DATE = 11/01/2021
- FILE_SIZE_IN_BYTES = 122349569
- GOOGLE_CLOUD_STORAGE_URL = https://storage.googleapis.com/pheknowlator/archived_builds/release_v3.0.2/build_01NOV2021/data/processed_data/clo_with_imports.owl

DATA INFO
- DOWNLOAD_URL = https://storage.googleapis.com/pheknowlator/archived_builds/release_v3.0.2/build_01NOV2021/data/original_data/ensembl_identifier_data_cleaned.txt
- DOWNLOAD_DATE = 11/01/2021
- FILE_SIZE_IN_BYTES = 31849231
- GOOGLE_CLOUD_STORAGE_URL = https://storage.googleapis.com/pheknowlator/archived_builds/release_v3.0.2/build_01NOV2021/data/processed_data/ensembl_identifier_data_cleaned.txt

DATA INFO
- DOWNLOAD_URL = https://storage.googleapis.com/pheknowlator/archived_builds/release_v3.0.2/build_01NOV2021/data/original_data/ext_with_imports.owl
- DOWNLOAD_DATE = 11/01/2021
- FILE_SIZE_IN_BYTES = 64227360
- GOOGLE_CLOUD_STORAGE_URL = https://storage.googleapis.com/pheknowlator/archived_builds/release_v3.0.2/build_01NOV2021/data/processed_data/ext_with_imports.owl

DATA INFO
- DOWNLOAD_URL = https://storage.googleapis.com/pheknowlator/archived_builds/release_v3.0.2/build_01NOV2021/data/original_data/go_with_imports.owl
- DOWNLOAD_DATE = 11/01/2021
- FILE_SIZE_IN_BYTES = 122488692
- GOOGLE_CLOUD_STORAGE_URL = https://storage.googleapis.com/pheknowlator/archived_builds/release_v3.0.2/build_01NOV2021/data/processed_data/go_with_imports.owl

DATA INFO
- DOWNLOAD_URL = https://storage.googleapis.com/pheknowlator/archived_builds/release_v3.0.2/build_01NOV2021/data/original_data/hp_with_imports.owl
- DOWNLOAD_DATE = 11/01/2021
- FILE_SIZE_IN_BYTES = 84205700
- GOOGLE_CLOUD_STORAGE_URL = https://storage.googleapis.com/pheknowlator/archived_builds/release_v3.0.2/build_01NOV2021/data/processed_data/hp_with_imports.owl

DATA INFO
- DOWNLOAD_URL = https://storage.googleapis.com/pheknowlator/archived_builds/release_v3.0.2/build_01NOV2021/data/original_data/human_pro.owl
- DOWNLOAD_DATE = 11/01/2021
- FILE_SIZE_IN_BYTES = 217854250
- GOOGLE_CLOUD_STORAGE_URL = https://storage.googleapis.com/pheknowlator/archived_builds/release_v3.0.2/build_01NOV2021/data/processed_data/human_pro.owl

DATA INFO
- DOWNLOAD_URL = https://storage.googleapis.com/pheknowlator/archived_builds/release_v3.0.2/build_01NOV2021/data/original_data/mondo_with_imports.owl
- DOWNLOAD_DATE = 11/01/2021
- FILE_SIZE_IN_BYTES = 231020989
- GOOGLE_CLOUD_STORAGE_URL = https://storage.googleapis.com/pheknowlator/archived_builds/release_v3.0.2/build_01NOV2021/data/processed_data/mondo_with_imports.owl

DATA INFO
- DOWNLOAD_URL = https://storage.googleapis.com/pheknowlator/archived_builds/release_v3.0.2/build_01NOV2021/data/original_data/node_metadata_dict.pkl
- DOWNLOAD_DATE = 11/01/2021
- FILE_SIZE_IN_BYTES = 325174359
- GOOGLE_CLOUD_STORAGE_URL = https://storage.googleapis.com/pheknowlator/archived_builds/release_v3.0.2/build_01NOV2021/data/processed_data/node_metadata_dict.pkl

DATA INFO
- DOWNLOAD_URL = https://storage.googleapis.com/pheknowlator/archived_builds/release_v3.0.2/build_01NOV2021/data/original_data/ontology_cleaning_report.txt
- DOWNLOAD_DATE = 11/01/2021
- FILE_SIZE_IN_BYTES = 1436530
- GOOGLE_CLOUD_STORAGE_URL = https://storage.googleapis.com/pheknowlator/archived_builds/release_v3.0.2/build_01NOV2021/data/processed_data/ontology_cleaning_report.txt

DATA INFO
- DOWNLOAD_URL = https://storage.googleapis.com/pheknowlator/archived_builds/release_v3.0.2/build_01NOV2021/data/original_data/pr_with_imports.owl

- DOWNLOAD_DATE = 11/01/2021
- FILE_SIZE_IN_BYTES = 217870791
- GOOGLE_CLOUD_STORAGE_URL = https://storage.googleapis.com/pheknowlator/archived_builds/release_v3.0.2/build_01NOV2021/data/processed_data/pr_with_imports.owl

DATA INFO
- DOWNLOAD_URL = https://storage.googleapis.com/pheknowlator/archived_builds/release_v3.0.2/build_01NOV2021/data/original_data/pw_with_imports.owl
- DOWNLOAD_DATE = 11/01/2021
- FILE_SIZE_IN_BYTES = 4915785
- GOOGLE_CLOUD_STORAGE_URL = https://storage.googleapis.com/pheknowlator/archived_builds/release_v3.0.2/build_01NOV2021/data/processed_data/pw_with_imports.owl

DATA INFO
- DOWNLOAD_URL = https://storage.googleapis.com/pheknowlator/archived_builds/release_v3.0.2/build_01NOV2021/data/original_data/ro_with_imports.owl
- DOWNLOAD_DATE = 11/01/2021
- FILE_SIZE_IN_BYTES = 857709
- GOOGLE_CLOUD_STORAGE_URL = https://storage.googleapis.com/pheknowlator/archived_builds/release_v3.0.2/build_01NOV2021/data/processed_data/ro_with_imports.owl

DATA INFO
- DOWNLOAD_URL = https://storage.googleapis.com/pheknowlator/archived_builds/release_v3.0.2/build_01NOV2021/data/original_data/so_with_imports.owl
- DOWNLOAD_DATE = 11/01/2021
- FILE_SIZE_IN_BYTES = 4890370
- GOOGLE_CLOUD_STORAGE_URL = https://storage.googleapis.com/pheknowlator/archived_builds/release_v3.0.2/build_01NOV2021/data/processed_data/so_with_imports.owl

DATA INFO
- DOWNLOAD_URL = https://storage.googleapis.com/pheknowlator/archived_builds/release_v3.0.2/build_01NOV2021/data/original_data/subclass_construction_map.pkl
- DOWNLOAD_DATE = 11/01/2021
- FILE_SIZE_IN_BYTES = 21922553
- GOOGLE_CLOUD_STORAGE_URL = https://storage.googleapis.com/pheknowlator/archived_builds/release_v3.0.2/build_01NOV2021/data/processed_data/subclass_construction_map.pkl

DATA INFO
- DOWNLOAD_URL = https://storage.googleapis.com/pheknowlator/archived_builds/release_v3.0.2/build_01NOV2021/data/original_data/vo_with_imports.owl
- DOWNLOAD_DATE = 11/01/2021
- FILE_SIZE_IN_BYTES = 8388461
- GOOGLE_CLOUD_STORAGE_URL = https://storage.googleapis.com/pheknowlator/archived_builds/release_v3.0.2/build_01NOV2021/data/processed_data/vo_with_imports.owl

Supplementary Document 3. edge_source_metadata.txt.

```
===================================
#Tue Nov 02 01:06:42 UTC 2021
===================================
EDGE: chemical-disease
DATA PROCESSING INFO
  - IDENTIFIER MAPPING = chemical (./resources/processed_data/MESH_CHEBI_MAP.txt) | disease (./resources/processed_data/DISEASE_MONDO_MAP.txt)
  - FILTERING CRITERIA = None
  - EVIDENCE CRITERIA = data[5]!=''
DATA INFO
  - DOWNLOAD_URL = https://storage.googleapis.com/pheknowlator/archived_builds/release_v3.0.2/build_01NOV2021/data/original_data/CTD_chemicals_diseases.tsv
  - DOWNLOAD_DATE = 11/02/2021
  - FILE_SIZE_IN_BYTES = 701201197
  - DOWNLOADED_FILE_LOCATION = resources/edge_data/chemical-disease_CTD_chemicals_diseases.tsv

EDGE: chemical-gene
DATA PROCESSING INFO
  - IDENTIFIER MAPPING = chemical (./resources/processed_data/MESH_CHEBI_MAP.txt)
  - FILTERING CRITERIA = data[6]==Homo sapiens | data[5].startswith('gene')
  - EVIDENCE CRITERIA = data[9]affectsnot in x
DATA INFO
  - DOWNLOAD_URL = https://storage.googleapis.com/pheknowlator/archived_builds/release_v3.0.2/build_01NOV2021/data/original_data/CTD_chem_gene_ixns.tsv
  - DOWNLOAD_DATE = 11/02/2021
  - FILE_SIZE_IN_BYTES = 441097103
  - DOWNLOADED_FILE_LOCATION = resources/edge_data/chemical-gene_CTD_chem_gene_ixns.tsv

EDGE: chemical-gobp
DATA PROCESSING INFO
  - IDENTIFIER MAPPING = chemical (./resources/processed_data/MESH_CHEBI_MAP.txt)
  - FILTERING CRITERIA = data[3]==Biological Process
  - EVIDENCE CRITERIA = data[8]<=1.04e-47
DATA INFO
  - DOWNLOAD_URL = https://storage.googleapis.com/pheknowlator/archived_builds/release_v3.0.2/build_01NOV2021/data/original_data/CTD_chem_go_enriched.tsv
  - DOWNLOAD_DATE = 11/02/2021
  - FILE_SIZE_IN_BYTES = 817733569
  - DOWNLOADED_FILE_LOCATION = resources/edge_data/chemical-gobp_CTD_chem_go_enriched.tsv

EDGE: chemical-gocc
DATA PROCESSING INFO
  - IDENTIFIER MAPPING = chemical (./resources/processed_data/MESH_CHEBI_MAP.txt)
  - FILTERING CRITERIA = data[3]==Cellular Component
  - EVIDENCE CRITERIA = data[8]<=1.04e-47
DATA INFO
  - DOWNLOAD_URL = https://storage.googleapis.com/pheknowlator/archived_builds/release_v3.0.2/build_01NOV2021/data/original_data/CTD_chem_go_enriched.tsv
  - DOWNLOAD_DATE = 11/02/2021
  - FILE_SIZE_IN_BYTES = 817733569
  - DOWNLOADED_FILE_LOCATION = resources/edge_data/chemical-gocc_CTD_chem_go_enriched.tsv

EDGE: chemical-gomf
DATA PROCESSING INFO
  - IDENTIFIER MAPPING = chemical (./resources/processed_data/MESH_CHEBI_MAP.txt)
  - FILTERING CRITERIA = data[3]==Molecular Function
```

- EVIDENCE CRITERIA = data[8]<=1.04e-47

DATA INFO
- DOWNLOAD_URL = https://storage.googleapis.com/pheknowlator/archived_builds/release_v3.0.2/build_01NOV2021/data/original_data/CTD_chem_go_enriched.tsv
- DOWNLOAD_DATE = 11/02/2021
- FILE_SIZE_IN_BYTES = 817733569
- DOWNLOADED_FILE_LOCATION = resources/edge_data/chemical-gomf_CTD_chem_go_enriched.tsv

EDGE: chemical-pathway

DATA PROCESSING INFO
- IDENTIFIER MAPPING = None
- FILTERING CRITERIA = data[5]==Homo sapiens
- EVIDENCE CRITERIA = None

DATA INFO
- DOWNLOAD_URL = https://storage.googleapis.com/pheknowlator/archived_builds/release_v3.0.2/build_01NOV2021/data/original_data/ChEBI2Reactome_All_Levels.txt
- DOWNLOAD_DATE = 11/02/2021
- FILE_SIZE_IN_BYTES = 30485396
- DOWNLOADED_FILE_LOCATION = resources/edge_data/chemical-pathway_ChEBI2Reactome_All_Levels.txt

EDGE: chemical-phenotype

DATA PROCESSING INFO
- IDENTIFIER MAPPING = chemical (./resources/processed_data/MESH_CHEBI_MAP.txt) | phenotype (./resources/processed_data/PHENOTYPE_HPO_MAP.txt)
- FILTERING CRITERIA = None
- EVIDENCE CRITERIA = data[5]!=''

DATA INFO
- DOWNLOAD_URL = https://storage.googleapis.com/pheknowlator/archived_builds/release_v3.0.2/build_01NOV2021/data/original_data/CTD_chemicals_diseases.tsv
- DOWNLOAD_DATE = 11/02/2021
- FILE_SIZE_IN_BYTES = 701201197
- DOWNLOADED_FILE_LOCATION = resources/edge_data/chemical-phenotype_CTD_chemicals_diseases.tsv

EDGE: chemical-protein

DATA PROCESSING INFO
- IDENTIFIER MAPPING = chemical (./resources/processed_data/MESH_CHEBI_MAP.txt) | protein (./resources/processed_data/ENTREZ_GENE_PRO_ONTOLOGY_MAP.txt)
- FILTERING CRITERIA = data[6]==Homo sapiens | data[5] .startswith('protein')
- EVIDENCE CRITERIA = data[9]affectsnot in x

DATA INFO
- DOWNLOAD_URL = https://storage.googleapis.com/pheknowlator/archived_builds/release_v3.0.2/build_01NOV2021/data/original_data/CTD_chem_gene_ixns.tsv
- DOWNLOAD_DATE = 11/02/2021
- FILE_SIZE_IN_BYTES = 441097103
- DOWNLOADED_FILE_LOCATION = resources/edge_data/chemical-protein_CTD_chem_gene_ixns.tsv

EDGE: disease-phenotype

DATA PROCESSING INFO
- IDENTIFIER MAPPING = None
- FILTERING CRITERIA = None
- EVIDENCE CRITERIA = None

DATA INFO
- DOWNLOAD_URL = https://storage.googleapis.com/pheknowlator/archived_builds/release_v3.0.2/build_01NOV2021/data/original_data/phenotype.hpoa
- DOWNLOAD_DATE = 11/02/2021
- FILE_SIZE_IN_BYTES = 27315334
- DOWNLOADED_FILE_LOCATION = resources/edge_data/disease-phenotype_phenotype.hpoa

EDGE: gene-disease

DATA PROCESSING INFO

- IDENTIFIER MAPPING = disease (./resources/processed_data/DISEASE_MONDO_MAP.txt)
- FILTERING CRITERIA = None
- EVIDENCE CRITERIA = data[10]>=1.0

DATA INFO
- DOWNLOAD_URL = https://storage.googleapis.com/pheknowlator/archived_builds/release_v3.0.2/build_01NOV2021/data/original_data/curated_gene_disease_associations.tsv
- DOWNLOAD_DATE = 11/02/2021
- FILE_SIZE_IN_BYTES = 11542996
- DOWNLOADED_FILE_LOCATION = resources/edge_data/gene-disease_curated_gene_disease_associations.tsv

EDGE: gene-gene
DATA PROCESSING INFO
- IDENTIFIER MAPPING = gene (./resources/processed_data/ENSEMBL_GENE_ENTREZ_GENE_MAP.txt) | gene (./resources/processed_data/ENSEMBL_GENE_ENTREZ_GENE_MAP.txt)
- FILTERING CRITERIA = None
- EVIDENCE CRITERIA = None

DATA INFO
- DOWNLOAD_URL = https://storage.googleapis.com/pheknowlator/archived_builds/release_v3.0.2/build_01NOV2021/data/original_data/COMBINED.DEFAULT_NETWORKS.BP_COMBINING.txt
- DOWNLOAD_DATE = 11/02/2021
- FILE_SIZE_IN_BYTES = 246190113
- DOWNLOADED_FILE_LOCATION = resources/edge_data/gene-gene_COMBINED.DEFAULT_NETWORKS.BP_COMBINING.txt

EDGE: gene-pathway
DATA PROCESSING INFO
- IDENTIFIER MAPPING = None
- FILTERING CRITERIA = data[3].startswith('REACT:R-HSA-')
- EVIDENCE CRITERIA = None

DATA INFO
- DOWNLOAD_URL = https://storage.googleapis.com/pheknowlator/archived_builds/release_v3.0.2/build_01NOV2021/data/original_data/CTD_genes_pathways.tsv
- DOWNLOAD_DATE = 11/02/2021
- FILE_SIZE_IN_BYTES = 8192661
- DOWNLOADED_FILE_LOCATION = resources/edge_data/gene-pathway_CTD_genes_pathways.tsv

EDGE: gene-phenotype
DATA PROCESSING INFO
- IDENTIFIER MAPPING = phenotype (./resources/processed_data/PHENOTYPE_HPO_MAP.txt)
- FILTERING CRITERIA = None
- EVIDENCE CRITERIA = data[10]>=1.0

DATA INFO
- DOWNLOAD_URL = https://storage.googleapis.com/pheknowlator/archived_builds/release_v3.0.2/build_01NOV2021/data/original_data/curated_gene_disease_associations.tsv
- DOWNLOAD_DATE = 11/02/2021
- FILE_SIZE_IN_BYTES = 11542996
- DOWNLOADED_FILE_LOCATION = resources/edge_data/gene-phenotype_curated_gene_disease_associations.tsv

EDGE: gene-protein
DATA PROCESSING INFO
- IDENTIFIER MAPPING = None
- FILTERING CRITERIA = data[4]==protein-coding
- EVIDENCE CRITERIA = None

DATA INFO
- DOWNLOAD_URL = https://storage.googleapis.com/pheknowlator/archived_builds/release_v3.0.2/build_01NOV2021/data/processed_data/ENTREZ_GENE_PRO_ONTOLOGY_MAP.txt
- DOWNLOAD_DATE = 11/02/2021
- FILE_SIZE_IN_BYTES = 1124944
- DOWNLOADED_FILE_LOCATION = resources/edge_data/gene-protein_ENTREZ_GENE_PRO_ONTOLOGY_MAP.txt

EDGE: gene-rna
DATA PROCESSING INFO
- IDENTIFIER MAPPING = None
- FILTERING CRITERIA = None
- EVIDENCE CRITERIA = None
DATA INFO
- DOWNLOAD_URL = https://storage.googleapis.com/pheknowlator/archived_builds/release_v3.0.2/build_01NOV2021/data/processed_data/ENTREZ_GENE_ENSEMBL_TRANSCRIPT_MAP.txt
- DOWNLOAD_DATE = 11/02/2021
- FILE_SIZE_IN_BYTES = 15146432
- DOWNLOADED_FILE_LOCATION = resources/edge_data/gene-rna_ENTREZ_GENE_ENSEMBL_TRANSCRIPT_MAP.txt

EDGE: gobp-pathway
DATA PROCESSING INFO
- IDENTIFIER MAPPING = None
- FILTERING CRITERIA = data[8]==P | data[12]==taxon:9606 | data[5].startswith('REACTOME')
- EVIDENCE CRITERIA = None
DATA INFO
- DOWNLOAD_URL = https://storage.googleapis.com/pheknowlator/archived_builds/release_v3.0.2/build_01NOV2021/data/original_data/gene_association.reactome
- DOWNLOAD_DATE = 11/02/2021
- FILE_SIZE_IN_BYTES = 11955743
- DOWNLOADED_FILE_LOCATION = resources/edge_data/gobp-pathway_gene_association.reactome

EDGE: pathway-gocc
DATA PROCESSING INFO
- IDENTIFIER MAPPING = None
- FILTERING CRITERIA = data[8]==C | data[12]==taxon:9606 | data[5].startswith('REACTOME')
- EVIDENCE CRITERIA = None
DATA INFO
- DOWNLOAD_URL = https://storage.googleapis.com/pheknowlator/archived_builds/release_v3.0.2/build_01NOV2021/data/original_data/gene_association.reactome
- DOWNLOAD_DATE = 11/02/2021
- FILE_SIZE_IN_BYTES = 11955743
- DOWNLOADED_FILE_LOCATION = resources/edge_data/pathway-gocc_gene_association.reactome

EDGE: pathway-gomf
DATA PROCESSING INFO
- IDENTIFIER MAPPING = None
- FILTERING CRITERIA = data[8]==F | data[12]==taxon:9606 | data[5].startswith('REACTOME')
- EVIDENCE CRITERIA = None
DATA INFO
- DOWNLOAD_URL = https://storage.googleapis.com/pheknowlator/archived_builds/release_v3.0.2/build_01NOV2021/data/original_data/gene_association.reactome
- DOWNLOAD_DATE = 11/02/2021
- FILE_SIZE_IN_BYTES = 11955743
- DOWNLOADED_FILE_LOCATION = resources/edge_data/pathway-gomf_gene_association.reactome

EDGE: protein-anatomy
DATA PROCESSING INFO
- IDENTIFIER MAPPING = protein (./resources/processed_data/UNIPROT_ACCESSION_PRO_ONTOLOGY_MAP.txt) | anatomy (./resources/processed_data/HPA_GTEx_TISSUE_CELL_MAP.txt)
- FILTERING CRITERIA = data[3]==Evidence at protein level | data[4]==anatomy
- EVIDENCE CRITERIA = None
DATA INFO
- DOWNLOAD_URL = https://storage.googleapis.com/pheknowlator/archived_builds/release_v3.0.2/build_01NOV2021/data/processed_data/HPA_GTEX_RNA_GENE_PROTEIN_EDGES.txt
- DOWNLOAD_DATE = 11/02/2021
- FILE_SIZE_IN_BYTES = 20611429
- DOWNLOADED_FILE_LOCATION = resources/edge_data/protein-anatomy_HPA_GTEX_RNA_GENE_PROTEIN_EDGES.txt

EDGE: protein-catalyst
DATA PROCESSING INFO
- IDENTIFIER MAPPING = None
- FILTERING CRITERIA = None
- EVIDENCE CRITERIA = None
DATA INFO
- DOWNLOAD_URL = https://storage.googleapis.com/pheknowlator/archived_builds/release_v3.0.2/build_01NOV2021/data/processed_data/UNIPROT_PROTEIN_CATALYST.txt
- DOWNLOAD_DATE = 11/02/2021
- FILE_SIZE_IN_BYTES = 1639479
- DOWNLOADED_FILE_LOCATION = resources/edge_data/protein-catalyst_UNIPROT_PROTEIN_CATALYST.txt

EDGE: protein-cell
DATA PROCESSING INFO
- IDENTIFIER MAPPING = protein (./resources/processed_data/UNIPROT_ACCESSION_PRO_ONTOLOGY_MAP.txt) | cell (./resources/processed_data/HPA_GTEx_TISSUE_CELL_MAP.txt)
- FILTERING CRITERIA = data[3]==Evidence at protein level | data[4]==cell line
- EVIDENCE CRITERIA = None
DATA INFO
- DOWNLOAD_URL = https://storage.googleapis.com/pheknowlator/archived_builds/release_v3.0.2/build_01NOV2021/data/processed_data/HPA_GTEX_RNA_GENE_PROTEIN_EDGES.txt
- DOWNLOAD_DATE = 11/02/2021
- FILE_SIZE_IN_BYTES = 20611429
- DOWNLOADED_FILE_LOCATION = resources/edge_data/protein-cell_HPA_GTEX_RNA_GENE_PROTEIN_EDGES.txt

EDGE: protein-cofactor
DATA PROCESSING INFO
- IDENTIFIER MAPPING = None
- FILTERING CRITERIA = None
- EVIDENCE CRITERIA = None
DATA INFO
- DOWNLOAD_URL = https://storage.googleapis.com/pheknowlator/archived_builds/release_v3.0.2/build_01NOV2021/data/processed_data/UNIPROT_PROTEIN_COFACTOR.txt
- DOWNLOAD_DATE = 11/02/2021
- FILE_SIZE_IN_BYTES = 179314
- DOWNLOADED_FILE_LOCATION = resources/edge_data/protein-cofactor_UNIPROT_PROTEIN_COFACTOR.txt

EDGE: protein-gobp
DATA PROCESSING INFO
- IDENTIFIER MAPPING = protein (./resources/processed_data/UNIPROT_ACCESSION_PRO_ONTOLOGY_MAP.txt)
- FILTERING CRITERIA = data[8]==P | data[12]==taxon:9606
- EVIDENCE CRITERIA = None
DATA INFO
- DOWNLOAD_URL = https://storage.googleapis.com/pheknowlator/archived_builds/release_v3.0.2/build_01NOV2021/data/original_data/goa_human.gaf
- DOWNLOAD_DATE = 11/02/2021
- FILE_SIZE_IN_BYTES = 109325311
- DOWNLOADED_FILE_LOCATION = resources/edge_data/protein-gobp_goa_human.gaf

EDGE: protein-gocc
DATA PROCESSING INFO
- IDENTIFIER MAPPING = protein (./resources/processed_data/UNIPROT_ACCESSION_PRO_ONTOLOGY_MAP.txt)
- FILTERING CRITERIA = data[8]==C | data[12]==taxon:9606
- EVIDENCE CRITERIA = None
DATA INFO
- DOWNLOAD_URL = https://storage.googleapis.com/pheknowlator/archived_builds/release_v3.0.2/build_01NOV2021/data/original_data/goa_human.gaf
- DOWNLOAD_DATE = 11/02/2021

- FILE_SIZE_IN_BYTES = 109325311
- DOWNLOADED_FILE_LOCATION = resources/edge_data/protein-gocc_goa_human.gaf

EDGE: protein-gomf
DATA PROCESSING INFO
- IDENTIFIER MAPPING = protein (./resources/processed_data/UNIPROT_ACCESSION_PRO_ONTOLOGY_MAP.txt)
- FILTERING CRITERIA = data[8]==F | data[12]==taxon:9606
- EVIDENCE CRITERIA = None
DATA INFO
- DOWNLOAD_URL = https://storage.googleapis.com/pheknowlator/archived_builds/release_v3.0.2/build_01NOV2021/data/original_data/goa_human.gaf
- DOWNLOAD_DATE = 11/02/2021
- FILE_SIZE_IN_BYTES = 109325311
- DOWNLOADED_FILE_LOCATION = resources/edge_data/protein-gomf_goa_human.gaf

EDGE: protein-pathway
DATA PROCESSING INFO
- IDENTIFIER MAPPING = protein (./resources/processed_data/UNIPROT_ACCESSION_PRO_ONTOLOGY_MAP.txt)
- FILTERING CRITERIA = data[5]==Homo sapiens
- EVIDENCE CRITERIA = None
DATA INFO
- DOWNLOAD_URL = https://storage.googleapis.com/pheknowlator/archived_builds/release_v3.0.2/build_01NOV2021/data/original_data/UniProt2Reactome_All_Levels.txt
- DOWNLOAD_DATE = 11/02/2021
- FILE_SIZE_IN_BYTES = 102191227
- DOWNLOADED_FILE_LOCATION = resources/edge_data/protein-pathway_UniProt2Reactome_All_Levels.txt

EDGE: protein-protein
DATA PROCESSING INFO
- IDENTIFIER MAPPING = protein (./resources/processed_data/STRING_PRO_ONTOLOGY_MAP.txt) | protein (./resources/processed_data/STRING_PRO_ONTOLOGY_MAP.txt)
- FILTERING CRITERIA = None
- EVIDENCE CRITERIA = data[2]>=700
DATA INFO
- DOWNLOAD_URL = https://storage.googleapis.com/pheknowlator/archived_builds/release_v3.0.2/build_01NOV2021/data/original_data/9606.protein.links.v11.0.txt
- DOWNLOAD_DATE = 11/02/2021
- FILE_SIZE_IN_BYTES = 540934917
- DOWNLOADED_FILE_LOCATION = resources/edge_data/protein-protein_9606.protein.links.v11.0.txt

EDGE: rna-anatomy
DATA PROCESSING INFO
- IDENTIFIER MAPPING = rna (./resources/processed_data/GENE_SYMBOL_ENSEMBL_TRANSCRIPT_MAP.txt) | anatomy (./resources/processed_data/HPA_GTEx_TISSUE_CELL_MAP.txt)
- FILTERING CRITERIA = data[3]==Evidence at transcript level | data[4]==anatomy
- EVIDENCE CRITERIA = None
DATA INFO
- DOWNLOAD_URL = https://storage.googleapis.com/pheknowlator/archived_builds/release_v3.0.2/build_01NOV2021/data/processed_data/HPA_GTEX_RNA_GENE_PROTEIN_EDGES.txt
- DOWNLOAD_DATE = 11/02/2021
- FILE_SIZE_IN_BYTES = 20611429
- DOWNLOADED_FILE_LOCATION = resources/edge_data/rna-anatomy_HPA_GTEX_RNA_GENE_PROTEIN_EDGES.txt

EDGE: rna-cell
DATA PROCESSING INFO
- IDENTIFIER MAPPING = rna (./resources/processed_data/GENE_SYMBOL_ENSEMBL_TRANSCRIPT_MAP.txt) | cell (./resources/processed_data/HPA_GTEx_TISSUE_CELL_MAP.txt)
- FILTERING CRITERIA = data[3]==Evidence at transcript level | data[4]==cell line
- EVIDENCE CRITERIA = None
DATA INFO

- DOWNLOAD_URL = https://storage.googleapis.com/pheknowlator/archived_builds/release_v3.0.2/build_01NOV2021/data/processed_data/HPA_GTEX_RNA_GENE_PROTEIN_EDGES.txt
- DOWNLOAD_DATE = 11/02/2021
- FILE_SIZE_IN_BYTES = 20611429
- DOWNLOADED_FILE_LOCATION = resources/edge_data/rna-cell_HPA_GTEX_RNA_GENE_PROTEIN_EDGES.txt

EDGE: rna-protein
DATA PROCESSING INFO
- IDENTIFIER MAPPING = None
- FILTERING CRITERIA = data[4]==protein-coding
- EVIDENCE CRITERIA = None

DATA INFO
- DOWNLOAD_URL = https://storage.googleapis.com/pheknowlator/archived_builds/release_v3.0.2/build_01NOV2021/data/processed_data/ENSEMBL_TRANSCRIPT_PROTEIN_ONTOLOGY_MAP.txt
- DOWNLOAD_DATE = 11/02/2021
- FILE_SIZE_IN_BYTES = 2931638
- DOWNLOADED_FILE_LOCATION = resources/edge_data/rna-protein_ENSEMBL_TRANSCRIPT_PROTEIN_ONTOLOGY_MAP.txt

EDGE: variant-disease
DATA PROCESSING INFO
- IDENTIFIER MAPPING = disease (./resources/processed_data/DISEASE_MONDO_MAP.txt)
- FILTERING CRITERIA = data[9]!=-1 | data[16]==GRCh38 | data[8-9]dedupdesc
- EVIDENCE CRITERIA = data[24] in ["criteria provided, multiple submitters, no conflicts", "reviewed by expert panel", "practice guideline"] | data[7]==1

DATA INFO
- DOWNLOAD_URL = https://storage.googleapis.com/pheknowlator/archived_builds/release_v3.0.2/build_01NOV2021/data/processed_data/CLINVAR_VARIANT_GENE_DISEASE_PHENOTYPE_EDGES.txt
- DOWNLOAD_DATE = 11/02/2021
- FILE_SIZE_IN_BYTES = 3662353455
- DOWNLOADED_FILE_LOCATION = resources/edge_data/variant-disease_CLINVAR_VARIANT_GENE_DISEASE_PHENOTYPE_EDGES.txt

EDGE: variant-gene
DATA PROCESSING INFO
- IDENTIFIER MAPPING = None
- FILTERING CRITERIA = data[9]!=-1 | data[3]!=-1 | data[16]==GRCh38 | data[8-9]dedupdesc
- EVIDENCE CRITERIA = data[24] in ["criteria provided, multiple submitters, no conflicts", "reviewed by expert panel", "practice guideline"]

DATA INFO
- DOWNLOAD_URL = https://storage.googleapis.com/pheknowlator/archived_builds/release_v3.0.2/build_01NOV2021/data/processed_data/CLINVAR_VARIANT_GENE_DISEASE_PHENOTYPE_EDGES.txt
- DOWNLOAD_DATE = 11/02/2021
- FILE_SIZE_IN_BYTES = 3662353455
- DOWNLOADED_FILE_LOCATION = resources/edge_data/variant-gene_CLINVAR_VARIANT_GENE_DISEASE_PHENOTYPE_EDGES.txt

EDGE: variant-phenotype
DATA PROCESSING INFO
- IDENTIFIER MAPPING = phenotype (./resources/processed_data/PHENOTYPE_HPO_MAP.txt)
- FILTERING CRITERIA = data[9]!=-1 | data[16]==GRCh38 | data[8-9]dedupdesc
- EVIDENCE CRITERIA = data[24] in ["criteria provided, multiple submitters, no conflicts", "reviewed by expert panel", "practice guideline"] | data[7]==1

DATA INFO
- DOWNLOAD_URL = https://storage.googleapis.com/pheknowlator/archived_builds/release_v3.0.2/build_01NOV2021/data/processed_data/CLINVAR_VARIANT_GENE_DISEASE_PHENOTYPE_EDGES.txt
- DOWNLOAD_DATE = 11/02/2021
- FILE_SIZE_IN_BYTES = 3662353455
- DOWNLOADED_FILE_LOCATION = resources/edge_data/variant-phenotype_CLINVAR_VARIANT_GENE_DISEASE_PHENOTYPE_EDGES.txt

Supplementary Document 4. ontology_source_metadata.txt.

```
===================================
#Tue Nov 02 01:05:41 UTC 2021
===================================

EDGE: phenotype
DATA PROCESSING INFO
  - IDENTIFIER MAPPING = None
  - FILTERING CRITERIA = None
  - EVIDENCE CRITERIA = None
DATA INFO
  - DOWNLOAD_URL = https://storage.googleapis.com/pheknowlator/archived_builds/release_v3.0.2/build_01NOV2021/data/processed_data/hp_with_imports.owl
  - DOWNLOAD_DATE = 11/02/2021
  - FILE_SIZE_IN_BYTES = 84205700
  - DOWNLOADED_FILE_LOCATION = resources/ontologies/hp_with_imports_with_imports.owl

EDGE: go
DATA PROCESSING INFO
  - IDENTIFIER MAPPING = None
  - FILTERING CRITERIA = None
  - EVIDENCE CRITERIA = None
DATA INFO
  - DOWNLOAD_URL = https://storage.googleapis.com/pheknowlator/archived_builds/release_v3.0.2/build_01NOV2021/data/processed_data/go_with_imports.owl
  - DOWNLOAD_DATE = 11/02/2021
  - FILE_SIZE_IN_BYTES = 122488692
  - DOWNLOADED_FILE_LOCATION = resources/ontologies/go_with_imports_with_imports.owl

EDGE: disease
DATA PROCESSING INFO
  - IDENTIFIER MAPPING = None
  - FILTERING CRITERIA = None
  - EVIDENCE CRITERIA = None
DATA INFO
  - DOWNLOAD_URL = https://storage.googleapis.com/pheknowlator/archived_builds/release_v3.0.2/build_01NOV2021/data/processed_data/mondo_with_imports.owl
  - DOWNLOAD_DATE = 11/02/2021
  - FILE_SIZE_IN_BYTES = 231020989
  - DOWNLOADED_FILE_LOCATION = resources/ontologies/mondo_with_imports_with_imports.owl

EDGE: vaccine
DATA PROCESSING INFO
  - IDENTIFIER MAPPING = None
  - FILTERING CRITERIA = None
  - EVIDENCE CRITERIA = None
DATA INFO
  - DOWNLOAD_URL = https://storage.googleapis.com/pheknowlator/archived_builds/release_v3.0.2/build_01NOV2021/data/processed_data/vo_with_imports.owl
  - DOWNLOAD_DATE = 11/02/2021
  - FILE_SIZE_IN_BYTES = 8388461
  - DOWNLOADED_FILE_LOCATION = resources/ontologies/vo_with_imports_with_imports.owl

EDGE: chemical
DATA PROCESSING INFO
  - IDENTIFIER MAPPING = None
  - FILTERING CRITERIA = None
```

- EVIDENCE CRITERIA = None
DATA INFO
- DOWNLOAD_URL = https://storage.googleapis.com/pheknowlator/archived_builds/release_v3.0.2/build_01NOV2021/data/processed_data/chebi_with_imports.owl
- DOWNLOAD_DATE = 11/02/2021
- FILE_SIZE_IN_BYTES = 643473711
- DOWNLOADED_FILE_LOCATION = resources/ontologies/chebi_with_imports_with_imports.owl

EDGE: anatomy
DATA PROCESSING INFO
- IDENTIFIER MAPPING = None
- FILTERING CRITERIA = None
- EVIDENCE CRITERIA = None
DATA INFO
- DOWNLOAD_URL = https://storage.googleapis.com/pheknowlator/archived_builds/release_v3.0.2/build_01NOV2021/data/processed_data/ext_with_imports.owl
- DOWNLOAD_DATE = 11/02/2021
- FILE_SIZE_IN_BYTES = 64227360
- DOWNLOADED_FILE_LOCATION = resources/ontologies/ext_with_imports_with_imports.owl

EDGE: cell
DATA PROCESSING INFO
- IDENTIFIER MAPPING = None
- FILTERING CRITERIA = None
- EVIDENCE CRITERIA = None
DATA INFO
- DOWNLOAD_URL = https://storage.googleapis.com/pheknowlator/archived_builds/release_v3.0.2/build_01NOV2021/data/processed_data/clo_with_imports.owl
- DOWNLOAD_DATE = 11/02/2021
- FILE_SIZE_IN_BYTES = 122349569
- DOWNLOADED_FILE_LOCATION = resources/ontologies/clo_with_imports_with_imports.owl

EDGE: protein
DATA PROCESSING INFO
- IDENTIFIER MAPPING = None
- FILTERING CRITERIA = None
- EVIDENCE CRITERIA = None
DATA INFO
- DOWNLOAD_URL = https://storage.googleapis.com/pheknowlator/archived_builds/release_v3.0.2/build_01NOV2021/data/processed_data/pr_with_imports.owl
- DOWNLOAD_DATE = 11/02/2021
- FILE_SIZE_IN_BYTES = 217870791
- DOWNLOADED_FILE_LOCATION = resources/ontologies/pr_with_imports_with_imports.owl

EDGE: genomic
DATA PROCESSING INFO
- IDENTIFIER MAPPING = None
- FILTERING CRITERIA = None
- EVIDENCE CRITERIA = None
DATA INFO
- DOWNLOAD_URL = https://storage.googleapis.com/pheknowlator/archived_builds/release_v3.0.2/build_01NOV2021/data/processed_data/so_with_imports.owl
- DOWNLOAD_DATE = 11/02/2021
- FILE_SIZE_IN_BYTES = 4890370
- DOWNLOADED_FILE_LOCATION = resources/ontologies/so_with_imports_with_imports.owl

EDGE: pathway
DATA PROCESSING INFO

- IDENTIFIER MAPPING = None
- FILTERING CRITERIA = None
- EVIDENCE CRITERIA = None

DATA INFO
- DOWNLOAD_URL = https://storage.googleapis.com/pheknowlator/archived_builds/release_v3.0.2/build_01NOV2021/data/processed_data/pw_with_imports.owl
- DOWNLOAD_DATE = 11/02/2021
- FILE_SIZE_IN_BYTES = 4915785
- DOWNLOADED_FILE_LOCATION = resources/ontologies/pw_with_imports_with_imports.owl

EDGE: relation

DATA PROCESSING INFO
- IDENTIFIER MAPPING = None
- FILTERING CRITERIA = None
- EVIDENCE CRITERIA = None

DATA INFO
- DOWNLOAD_URL = https://storage.googleapis.com/pheknowlator/archived_builds/release_v3.0.2/build_01NOV2021/data/processed_data/ro_with_imports.owl
- DOWNLOAD_DATE = 11/02/2021
- FILE_SIZE_IN_BYTES = 857709
- DOWNLOADED_FILE_LOCATION = resources/ontologies/ro_with_imports_with_imports.owl

Supplementary Document 5. ontology_cleaning_report.txt.

===
ONTOLOGY CLEANING REPORT
Mon Nov 01 09:57:17 UTC 2021
===

ONTOLOGY: chebi_with_imports.owl
****

- Original GCS URL: https://storage.googleapis.com/pheknowlator/archived_builds/release_v3.0.2/build_01NOV2021/data/original_data/chebi_with_imports.owl
- Processed GCS URL: https://storage.googleapis.com/pheknowlator/archived_builds/release_v3.0.2/build_01NOV2021/data/processed_data/chebi_with_imports.owl
- Statistics Before Cleaning: 5644239 Triples; 168627 Classes; 0 Individuals; 10 Object Properties; 37 Annotation Properties; 1 Connected Components
- Statistics After Cleaning: 5569987 Triples; 150080 Classes; 0 Individuals; 10 Object Properties; 37 Annotation Properties; 1 Connected Components
- Value Errors: 0
- Identifier Errors: 0
- Deprecated Classes: 18547
- Obsolete Classes: 0
- Punning Errors: 0
    - Object Properties: 0

ONTOLOGY: clo_with_imports.owl
****

- Original GCS URL: https://storage.googleapis.com/pheknowlator/archived_builds/release_v3.0.2/build_01NOV2021/data/original_data/clo_with_imports.owl
- Processed GCS URL: https://storage.googleapis.com/pheknowlator/archived_builds/release_v3.0.2/build_01NOV2021/data/processed_data/clo_with_imports.owl
- Statistics Before Cleaning: 1387096 Triples; 111712 Classes; 41 Individuals; 116 Object Properties; 192 Annotation Properties; 7 Connected Components
- Statistics After Cleaning: 1422153 Triples; 111696 Classes; 33 Individuals; 112 Object Properties; 187 Annotation Properties; 7 Connected Components
- Value Errors (n=1):
    - RDF/XML parsing error in file builds/temp/clo_with_imports.owl, line 10971, column 99.
- Identifier Errors: 0
- Deprecated Classes: 2
- Obsolete Classes: 13
- Punning Errors:
    - Classes (n=10):
        - http://purl.obolibrary.org/obo/UBERON_0000062
        - http://purl.obolibrary.org/obo/UBERON_0001017
        - http://purl.obolibrary.org/obo/UBERON_0001004
        - http://purl.obolibrary.org/obo/UBERON_0000926
        - http://purl.obolibrary.org/obo/UBERON_0000029
        - http://purl.obolibrary.org/obo/UBERON_0001009
        - http://purl.obolibrary.org/obo/CLO_0054407
        - http://purl.obolibrary.org/obo/UBERON_0000383
        - http://purl.obolibrary.org/obo/CLO_0054409
        - http://purl.obolibrary.org/obo/UBERON_0001555
    - Object Properties (n=6):
        - http://purl.obolibrary.org/obo/RO_0002222
        - http://purl.obolibrary.org/obo/BFO_0000062
        - http://purl.obolibrary.org/obo/BFO_0000063
        - http://purl.obolibrary.org/obo/RO_0002161
        - http://purl.obolibrary.org/obo/RO_0002091
        - http://purl.obolibrary.org/obo/RO_0000087

ONTOLOGY: ext_with_imports.owl
****

- Original GCS URL: https://storage.googleapis.com/pheknowlator/archived_builds/release_v3.0.2/build_01NOV2021/data/original_data/ext_with_imports.owl

- Processed GCS URL: https://storage.googleapis.com/pheknowlator/archived_builds/release_v3.0.2/build_01NOV2021/data/processed_data/ext_with_imports.owl
- Statistics Before Cleaning: 769010 Triples; 28166 Classes; 0 Individuals; 239 Object Properties; 282 Annotation Properties; 19 Connected Components
- Statistics After Cleaning: 750999 Triples; 26577 Classes; 0 Individuals; 239 Object Properties; 282 Annotation Properties; 19 Connected Components
- Value Errors: 0
- Identifier Errors: 0
- Deprecated Classes: 1589
- Obsolete Classes: 0
- Punning Errors: 0

ONTOLOGY: go_with_imports.owl
****
- Original GCS URL: https://storage.googleapis.com/pheknowlator/archived_builds/release_v3.0.2/build_01NOV2021/data/original_data/go_with_imports.owl
- Processed GCS URL: https://storage.googleapis.com/pheknowlator/archived_builds/release_v3.0.2/build_01NOV2021/data/processed_data/go_with_imports.owl
- Statistics Before Cleaning: 1425011 Triples; 62437 Classes; 0 Individuals; 9 Object Properties; 55 Annotation Properties; 2 Connected Components
- Statistics After Cleaning: 1334338 Triples; 55556 Classes; 0 Individuals; 9 Object Properties; 55 Annotation Properties; 2 Connected Components
- Value Errors: 0
- Identifier Errors: 0
- Deprecated Classes: 6881
- Obsolete Classes: 0
- Punning Errors: 0

ONTOLOGY: hp_with_imports.owl
****
- Original GCS URL: https://storage.googleapis.com/pheknowlator/archived_builds/release_v3.0.2/build_01NOV2021/data/original_data/hp_with_imports.owl
- Processed GCS URL: https://storage.googleapis.com/pheknowlator/archived_builds/release_v3.0.2/build_01NOV2021/data/processed_data/hp_with_imports.owl
- Statistics Before Cleaning: 934712 Triples; 41442 Classes; 0 Individuals; 256 Object Properties; 226 Annotation Properties; 1 Connected Components
- Statistics After Cleaning: 934877 Triples; 41125 Classes; 0 Individuals; 256 Object Properties; 226 Annotation Properties; 1 Connected Components
- Value Errors: 0
- Identifier Errors: 0
- Deprecated Classes: 313
- Obsolete Classes: 0
- Punning Errors: 0

ONTOLOGY: mondo_with_imports.owl
****
- Original GCS URL: https://storage.googleapis.com/pheknowlator/archived_builds/release_v3.0.2/build_01NOV2021/data/original_data/mondo_with_imports.owl
- Processed GCS URL: https://storage.googleapis.com/pheknowlator/archived_builds/release_v3.0.2/build_01NOV2021/data/processed_data/mondo_with_imports.owl
- Statistics Before Cleaning: 2375692 Triples; 58553 Classes; 18 Individuals; 339 Object Properties; 153 Annotation Properties; 1 Connected Components
- Statistics After Cleaning: 2336440 Triples; 55929 Classes; 17 Individuals; 338 Object Properties; 153 Annotation Properties; 1 Connected Components
- Value Errors: 0
- Identifier Errors: 0
- Deprecated Classes: 2622
- Obsolete Classes: 0
- Punning Errors: 0

ONTOLOGY: pr_with_imports.owl
****
- Original GCS URL: https://storage.googleapis.com/pheknowlator/archived_builds/release_v3.0.2/build_01NOV2021/data/original_data/pr_with_imports.owl
- Processed GCS URL: https://storage.googleapis.com/pheknowlator/archived_builds/release_v3.0.2/build_01NOV2021/data/processed_data/pr_with_imports.owl
- Statistics Before Cleaning: 2078128 Triples; 148164 Classes; 0 Individuals; 12 Object Properties; 11 Annotation Properties; 3 Connected Components
- Statistics After Cleaning: 2078128 Triples; 148164 Classes; 0 Individuals; 12 Object Properties; 11 Annotation Properties; 3 Connected Components
- Value Errors: 0
- Identifier Errors: 0

- Deprecated Classes: 0
- Obsolete Classes: 0
- Punning Errors: 0

ONTOLOGY: pw_with_imports.owl
****
- Original GCS URL: https://storage.googleapis.com/pheknowlator/archived_builds/release_v3.0.2/build_01NOV2021/data/original_data/pw_with_imports.owl
- Processed GCS URL: https://storage.googleapis.com/pheknowlator/archived_builds/release_v3.0.2/build_01NOV2021/data/processed_data/pw_with_imports.owl
- Statistics Before Cleaning: 35291 Triples; 2642 Classes; 0 Individuals; 1 Object Properties; 19 Annotation Properties; 1 Connected Components
- Statistics After Cleaning: 34901 Triples; 2600 Classes; 0 Individuals; 1 Object Properties; 19 Annotation Properties; 1 Connected Components
- Value Errors: 0
- Identifier Errors: 0
- Deprecated Classes: 42
- Obsolete Classes: 0
- Punning Errors: 0

ONTOLOGY: ro_with_imports.owl
****
- Original GCS URL: https://storage.googleapis.com/pheknowlator/archived_builds/release_v3.0.2/build_01NOV2021/data/original_data/ro_with_imports.owl
- Processed GCS URL: https://storage.googleapis.com/pheknowlator/archived_builds/release_v3.0.2/build_01NOV2021/data/processed_data/ro_with_imports.owl
- Statistics Before Cleaning: 8069 Triples; 99 Classes; 5 Individuals; 611 Object Properties; 113 Annotation Properties; 3 Connected Components
- Statistics After Cleaning: 7971 Triples; 99 Classes; 5 Individuals; 599 Object Properties; 113 Annotation Properties; 3 Connected Components
- Value Errors: 0
- Identifier Errors: 0
- Deprecated Classes: 12
- Obsolete Classes: 0
- Punning Errors: 0

ONTOLOGY: so_with_imports.owl
****
- Original GCS URL: https://storage.googleapis.com/pheknowlator/archived_builds/release_v3.0.2/build_01NOV2021/data/original_data/so_with_imports.owl
- Processed GCS URL: https://storage.googleapis.com/pheknowlator/archived_builds/release_v3.0.2/build_01NOV2021/data/processed_data/so_with_imports.owl
- Statistics Before Cleaning: 44890 Triples; 2925 Classes; 0 Individuals; 50 Object Properties; 41 Annotation Properties; 1 Connected Components
- Statistics After Cleaning: 42198 Triples; 2583 Classes; 0 Individuals; 50 Object Properties; 41 Annotation Properties; 1 Connected Components
- Value Errors: 0
- Identifier Errors: 0
- Deprecated Classes: 342
- Obsolete Classes: 0
- Punning Errors: 0

ONTOLOGY: vo_with_imports.owl
****
- Original GCS URL: https://storage.googleapis.com/pheknowlator/archived_builds/release_v3.0.2/build_01NOV2021/data/original_data/vo_with_imports.owl
- Processed GCS URL: https://storage.googleapis.com/pheknowlator/archived_builds/release_v3.0.2/build_01NOV2021/data/processed_data/vo_with_imports.owl
- Statistics Before Cleaning: 88373 Triples; 7198 Classes; 167 Individuals; 232 Object Properties; 99 Annotation Properties; 5 Connected Components
- Statistics After Cleaning: 91683 Triples; 7194 Classes; 167 Individuals; 232 Object Properties; 99 Annotation Properties; 5 Connected Components
- Value Errors: 0
- Identifier Errors (n=2):
    - http://purl.obolibrary.org/obo/PRO_000000001
    - http://purl.obolibrary.org/obo/PRO_000015399
- Deprecated Classes: 0
- Obsolete Classes: 0
- Punning Errors: 0

ONTOLOGY: PheKnowLator_MergedOntologies.owl
****

- Original GCS URL: https://storage.googleapis.com/pheknowlator/archived_builds/release_v3.0.2/build_01NOV2021/data/original_data/PheKnowLator_MergedOntologies.owl
- Processed GCS URL: https://storage.googleapis.com/pheknowlator/archived_builds/release_v3.0.2/build_01NOV2021/data/processed_data/PheKnowLator_MergedOntologies.owl
- Statistics Before Cleaning: 13934582 Triples; 562412 Classes; 197 Individuals; 853 Object Properties; 635 Annotation Properties; 8 Connected Components
- Statistics After Cleaning: 13935691 Triples; 558715 Classes; 190 Individuals; 852 Object Properties; 635 Annotation Properties; 8 Connected Components
- Value Errors: 0
- Identifier Errors (n=2):
    - http://purl.obolibrary.org/obo/PRO_000000001
    - http://purl.obolibrary.org/obo/PRO_000015399
- Punning Errors:
    - Classes (n=8):
        - http://purl.obolibrary.org/obo/NCBITaxon_147099
        - http://purl.obolibrary.org/obo/NCBITaxon_6040
        - http://purl.obolibrary.org/obo/CLO_0054409
        - http://purl.obolibrary.org/obo/NCBITaxon_6157
        - http://purl.obolibrary.org/obo/NCBITaxon_6073
        - http://purl.obolibrary.org/obo/NCBITaxon_41324
        - http://purl.obolibrary.org/obo/NCBITaxon_8570
        - http://purl.obolibrary.org/obo/NCBITaxon_110815
    - Object Properties: 0
- Normalization:
    - Normalized Entities (n=7):
        - OGG_0000000002 rdfs:subClassOf SO_0000704
        - PR_000000001 rdfs:subClassOf SO_0000104
        - CHEBI_36080 rdfs:subClassOf SO_0000104
        - OGMS_0000045 rdfs:subClassOf MONDO_0000001
        - OBI_1110034 rdfs:subClassOf CHEBI_59132
        - VO_0003030 rdfs:subClassOf CHEBI_5291
        - FMA_12278 rdfs:subClassOf CHEBI_24621
    - Normalized HGNC IDs: 23631
    - Other Classes that May Need Normalizing: 404495
    - Deprecated Ontology HGNC Identifiers Needing Alignment: 0

Supplementary Document 6. pkt_builder_phases12_log.log (Data Download and Preparation).

{"asctime": "2021-11-01 01:14:27,998", "levelname": "INFO", "name": "__main__", "module": "phases1_2_entrypoint", "funcName": "main", "lineno": 40, "message": "********** STARTING PHEKNOWLATOR KNOWLEDGE GRAPH BUILD **********"}
{"asctime": "2021-11-01 01:14:28,087", "levelname": "INFO", "name": "__main__", "module": "phases1_2_entrypoint", "funcName": "main", "lineno": 45, "message": "#####BUILD PHASE 1: DOWNLOADING BUILD DATA#####"}
{"asctime": "2021-11-01 01:14:28,148", "levelname": "INFO", "name": "builds.build_phase_1", "module": "build_phase_1", "funcName": "creates_build_directory_structure", "lineno": 46, "message": "Creating Build Directory Structure"}
{"asctime": "2021-11-01 01:14:29,081", "levelname": "INFO", "name": "builds.build_phase_1", "module": "build_phase_1", "funcName": "downloads_build_data", "lineno": 136, "message": "Downloading Build Data"}
{"asctime": "2021-11-01 01:14:29,111", "levelname": "INFO", "name": "builds.build_phase_1", "module": "build_phase_1", "funcName": "downloads_build_data", "lineno": 143, "message": "Downloading http://purl.obolibrary.org/obo/hp.owl"}
{"asctime": "2021-11-01 01:14:49,795", "levelname": "INFO", "name": "builds.build_phase_1", "module": "build_phase_1", "funcName": "downloads_build_data", "lineno": 143, "message": "Downloading http://purl.obolibrary.org/obo/go.owl"}
{"asctime": "2021-11-01 01:15:14,376", "levelname": "INFO", "name": "builds.build_phase_1", "module": "build_phase_1", "funcName": "downloads_build_data", "lineno": 143, "message": "Downloading http://purl.obolibrary.org/obo/mondo.owl"}
{"asctime": "2021-11-01 01:15:54,602", "levelname": "INFO", "name": "builds.build_phase_1", "module": "build_phase_1", "funcName": "downloads_build_data", "lineno": 143, "message": "Downloading http://purl.obolibrary.org/obo/vo.owl"}
{"asctime": "2021-11-01 01:15:59,539", "levelname": "INFO", "name": "builds.build_phase_1", "module": "build_phase_1", "funcName": "downloads_build_data", "lineno": 143, "message": "Downloading http://purl.obolibrary.org/obo/chebi.owl"}
{"asctime": "2021-11-01 01:35:06,926", "levelname": "INFO", "name": "builds.build_phase_1", "module": "build_phase_1", "funcName": "downloads_build_data", "lineno": 143, "message": "Downloading http://purl.obolibrary.org/obo/uberon/ext.owl"}
{"asctime": "2021-11-01 01:35:26,945", "levelname": "INFO", "name": "builds.build_phase_1", "module": "build_phase_1", "funcName": "downloads_build_data", "lineno": 143, "message": "Downloading http://purl.obolibrary.org/obo/clo.owl"}
{"asctime": "2021-11-01 01:35:56,608", "levelname": "INFO", "name": "builds.build_phase_1", "module": "build_phase_1", "funcName": "downloads_build_data", "lineno": 143, "message": "Downloading http://purl.obolibrary.org/obo/pr.owl"}
{"asctime": "2021-11-01 01:39:19,579", "levelname": "INFO", "name": "builds.build_phase_1", "module": "build_phase_1", "funcName": "downloads_build_data", "lineno": 143, "message": "Downloading http://purl.obolibrary.org/obo/so.owl"}
{"asctime": "2021-11-01 01:39:23,524", "levelname": "INFO", "name": "builds.build_phase_1", "module": "build_phase_1", "funcName": "downloads_build_data", "lineno": 143, "message": "Downloading http://purl.obolibrary.org/obo/pw.owl"}
{"asctime": "2021-11-01 01:39:26,893", "levelname": "INFO", "name": "builds.build_phase_1", "module": "build_phase_1", "funcName": "downloads_build_data", "lineno": 143, "message": "Downloading http://purl.obolibrary.org/obo/ro.owl"}
{"asctime": "2021-11-01 01:39:32,386", "levelname": "INFO", "name": "builds.build_phase_1", "module": "build_phase_1", "funcName": "downloads_build_data", "lineno": 143, "message": "Downloading hgnc_complete_set.txt,
http://ftp.ebi.ac.uk/pub/databases/genenames/hgnc/tsv/hgnc_complete_set.txt"}
{"asctime": "2021-11-01 01:39:39,226", "levelname": "INFO", "name": "builds.build_phase_1", "module": "build_phase_1", "funcName": "downloads_build_data", "lineno": 143, "message": "Downloading Homo_sapiens.GRCh38.102.gtf,
ftp://ftp.ensembl.org/pub/release-102/gtf/homo_sapiens/Homo_sapiens.GRCh38.102.gtf.gz"}
{"asctime": "2021-11-01 01:39:57,391", "levelname": "INFO", "name": "builds.build_phase_1", "module": "build_phase_1", "funcName": "downloads_build_data", "lineno": 143, "message": "Downloading Homo_sapiens.GRCh38.102.uniprot.tsv,
ftp://ftp.ensembl.org/pub/release-102/tsv/homo_sapiens/Homo_sapiens.GRCh38.102.uniprot.tsv.gz"}
{"asctime": "2021-11-01 01:39:59,843", "levelname": "INFO", "name": "builds.build_phase_1", "module": "build_phase_1", "funcName": "downloads_build_data", "lineno": 143, "message": "Downloading Homo_sapiens.GRCh38.102.entrez.tsv,
ftp://ftp.ensembl.org/pub/release-102/tsv/homo_sapiens/Homo_sapiens.GRCh38.102.entrez.tsv.gz"}
{"asctime": "2021-11-01 01:40:01,973", "levelname": "INFO", "name": "builds.build_phase_1", "module": "build_phase_1", "funcName": "downloads_build_data", "lineno": 143, "message": "Downloading uniprot_identifier_mapping.tab,
https://www.uniprot.org/uniprot/?query=&fil=organism%3A%22Homo%20sapiens%20(Human)%20%5B9606%5D%22&columns=id%2Creviewed%2Cdatabase(GeneID)%2Cdatabase(Ensembl)%2Cdatabase(HGNC)%2Cgenes(ALTERNATIVE)%2Cgenes(PREFERRED)&format=tab"}
{"asctime": "2021-11-01 01:41:20,156", "levelname": "INFO", "name": "builds.build_phase_1", "module": "build_phase_1", "funcName": "downloads_build_data", "lineno": 143, "message": "Downloading Homo_sapiens.gene_info,
ftp://ftp.ncbi.nih.gov/gene/DATA/GENE_INFO/Mammalia/Homo_sapiens.gene_info.gz"}
{"asctime": "2021-11-01 01:41:21,503", "levelname": "INFO", "name": "builds.build_phase_1", "module": "build_phase_1", "funcName": "downloads_build_data", "lineno": 143, "message": "Downloading promapping.txt, https://proconsortium.org/download/current/promapping.txt"}
{"asctime": "2021-11-01 01:41:22,649", "levelname": "INFO", "name": "builds.build_phase_1", "module": "build_phase_1", "funcName": "downloads_build_data", "lineno": 143, "message": "Downloading mesh2021.nt, ftp://nlmpubs.nlm.nih.gov/online/mesh/rdf/2021/mesh2021.nt"}
{"asctime": "2021-11-01 01:41:54,362", "levelname": "INFO", "name": "builds.build_phase_1", "module": "build_phase_1", "funcName": "downloads_build_data", "lineno": 143, "message": "Downloading names.tsv, ftp://ftp.ebi.ac.uk/pub/databases/chebi/Flat_file_tab_delimited/names.tsv.gz"}
{"asctime": "2021-11-01 01:41:57,241", "levelname": "INFO", "name": "builds.build_phase_1", "module": "build_phase_1", "funcName": "downloads_build_data", "lineno": 143, "message": "Downloading disease_mappings.tsv,
https://www.disgenet.org/static/disgenet_ap1/files/downloads/disease_mappings.tsv.gz"}
{"asctime": "2021-11-01 01:41:59,514", "levelname": "INFO", "name": "builds.build_phase_1", "module": "build_phase_1", "funcName": "downloads_build_data", "lineno": 143, "message": "Downloading proteinatlas_search.tsv.gz,
https://www.proteinatlas.org/api/search_download.php?search=&columns=g,eg,up,pe,rnatsm,rnaclsm,rnacasm,rnabrsm,rnabcsm,rnablsm,scl,t_RNA_adipose_tissue,t_RNA_adrenal_gland,t_RNA_amygdala,t_RNA_appendix,t_RNA_basal_ganglia,t_RNA_bone_marrow,t_RNA_breast,t_RNA_cereb
ellum,t_RNA_cerebral_cortex,t_RNA_cervix,_uterine,t_RNA_colon,t_RNA_corpus_callosum,t_RNA_ductus_deferens,t_RNA_duodenum,t_RNA_endometrium_1,t_RNA_epididymis,t_RNA_esophagus,t_RNA_fallopian_tube,t_RNA_gallbladder,t_RNA_heart_muscle,t_RNA_hippocampal_formation,t
_RNA_hypothalamus,t_RNA_kidney,t_RNA_liver,t_RNA_lung,t_RNA_lymph_node,t_RNA_midbrain,t_RNA_olfactory_region,t_RNA_ovary,t_RNA_pancreas,t_RNA_parathyroid_gland,t_RNA_pituitary_gland,t_RNA_placenta,t_RNA_pons_and_medulla,t_RNA_prostate,t_RNA_rectum,t_RNA_retina,t
_RNA_salivary_gland,t_RNA_seminal_vesicle,t_RNA_skeletal_muscle,t_RNA_skin_1,t_RNA_small_intestine,t_RNA_smooth_muscle,t_RNA_spinal_cord,t_RNA_spleen,t_RNA_stomach_1,t_RNA_testis,t_RNA_thalamus,t_RNA_thymus,t_RNA_thyroid_gland,t_RNA_tongue,t_RNA_tonsil,t_RNA_urin
ary_bladder,t_RNA_vagina,t_RNA_B-cells,t_RNA_dendritic_cells,t_RNA_granulocytes,t_RNA_monocytes,t_RNA_NK-cells,t_RNA_T-cells,t_RNA_total_PBMC,cell_RNA_A-431,cell_RNA_A549,cell_RNA_AF22,cell_RNA_AN3-CA,cell_RNA_ASC_diff,cell_RNA_ASC_TERT1,cell_RNA_BEWO,cell_RNA_BJ,c
ell_RNA_BJ_hTERT+,cell_RNA_BJ_hTERT+_SV40_Large_T+,cell_RNA_BJ_hTERT+_SV40_Large_T+_RasG12V,cell_RNA_CACO-2,cell_RNA_CAPAN-2,cell_RNA_Daudi,cell_RNA_EFO-21,cell_RNA_fHDF/TERT166,cell_RNA_HaCaT,cell_RNA_HAP1,cell_RNA_HBEC3-KT,cell_RNA_HBF_TERT88,cell_RNA_HD
LM-2,cell_RNA_HEK_293,cell_RNA_HEL,cell_RNA_HeLa,cell_RNA_Hep_G2,cell_RNA_HHSteC,cell_RNA_HL-60,cell_RNA_HMC-1,cell_RNA_HSkMC,cell_RNA_hTCEpi,cell_RNA_hTEC/SVTERT24-B,cell_RNA_hTERT-HME1,cell_RNA_HUVEC_TERT2,cell_RNA_K-562,cell_RNA_Karpas-707,cell_RNA_LHCN
-M2,cell_RNA_MCF7,cell_RNA_MOLT-4,cell_RNA_NB-4,cell_RNA_NTERA-2,cell_RNA_PC-3,cell_RNA_REH,cell_RNA_RH-30,cell_RNA_RPMI-8226,cell_RNA_RPTEC_TERT1,cell_RNA_RT4,cell_RNA_SCLC-21H,cell_RNA_SH-SY5Y,cell_RNA_SiHa,cell_RNA_SK-BR-3,cell_RNA_SK-MEL-30,cell_RNA_T-47d,c
ell_RNA_THP-1,cell_RNA_TIME,cell_RNA_U-138_MG,cell_RNA_U-2_OS,cell_RNA_U-2197,cell_RNA_U-251_MG,cell_RNA_U-266/70,cell_RNA_U-266/84,cell_RNA_U-698,cell_RNA_U-87_MG,cell_RNA_U-937,cell_RNA_WM-115,blood_RNA_basophil,blood_RNA_classical_monocyte,blood_RNA_e
osinophil,blood_RNA_gdT-cell,blood_RNA_intermediate_monocyte,blood_RNA_MAIT_T-cell,blood_RNA_memory_B-cell,blood_RNA_memory_CD4_T-cell,blood_RNA_memory_CD8_T-cell,blood_RNA_myeloid_DC,blood_RNA_naive_B-cell,blood_RNA_naive_CD4_T-cell,blood_RNA_naive_CD8_T-
cell,blood_RNA_neutrophil,blood_RNA_NK-cell,blood_RNA_non-classical_monocyte,blood_RNA_plasmacytoid_DC,blood_RNA_T-reg,blood_RNA_total_PBMC,brain_RNA_amygdala,brain_RNA_basal_ganglia,brain_RNA_cerebellum,brain_RNA_cerebral_cortex,brain_RNA_hippocampal_formation,
brain_RNA_hypothalamus,brain_RNA_midbrain,brain_RNA_olfactory_region,brain_RNA_pons_and_medulla,brain_RNA_thalamus&format=tsv"}
{"asctime": "2021-11-01 01:42:11,936", "levelname": "INFO", "name": "builds.build_phase_1", "module": "build_phase_1", "funcName": "downloads_build_data", "lineno": 143, "message": "Downloading GTEx_Analysis_2017-06-05_v8_RNASeQCv1.1.9_gene_median_tpm.gct,
https://storage.googleapis.com/gtex_analysis_v8/rna_seq_data/GTEx_Analysis_2017-06-05_v8_RNASeQCv1.1.9_gene_median_tpm.gct.gz"}
{"asctime": "2021-11-01 01:42:12,902", "levelname": "INFO", "name": "builds.build_phase_1", "module": "build_phase_1", "funcName": "downloads_build_data", "lineno": 143, "message": "Downloading ReactomePathways.txt, https://reactome.org/download/current/ReactomePathways.txt"}
{"asctime": "2021-11-01 01:42:13,467", "levelname": "INFO", "name": "builds.build_phase_1", "module": "build_phase_1", "funcName": "downloads_build_data", "lineno": 143, "message": "Downloading gene_association.reactome,
https://reactome.org/download/current/gene_association.reactome.gz"}
{"asctime": "2021-11-01 01:42:14,221", "levelname": "INFO", "name": "builds.build_phase_1", "module": "build_phase_1", "funcName": "downloads_build_data", "lineno": 143, "message": "Downloading ChEBI2Reactome_All_Levels.txt,
https://reactome.org/download/current/ChEBI2Reactome_All_Levels.txt"}
{"asctime": "2021-11-01 01:42:15,421", "levelname": "INFO", "name": "builds.build_phase_1", "module": "build_phase_1", "funcName": "downloads_build_data", "lineno": 143, "message": "Downloading compath_canonical_pathway_mappings.txt,
http://compath.scai.fraunhofer.de/export_mappings"}
{"asctime": "2021-11-01 01:42:17,215", "levelname": "INFO", "name": "builds.build_phase_1", "module": "build_phase_1", "funcName": "downloads_build_data", "lineno": 143, "message": "Downloading kegg_reactome.csv,
https://raw.githubusercontent.com/ComPath/resources/master/mappings/kegg_reactome.csv"}
{"asctime": "2021-11-01 01:42:17,653", "levelname": "INFO", "name": "builds.build_phase_1", "module": "build_phase_1", "funcName": "downloads_build_data", "lineno": 143, "message": "Downloading genomic_sequence_ontology_mappings.xlsx,
https://storage.googleapis.com/pheknowlator/curated_data/genomic_sequence_ontology_mappings.xlsx"}
{"asctime": "2021-11-01 01:42:18,028", "levelname": "INFO", "name": "builds.build_phase_1", "module": "build_phase_1", "funcName": "downloads_build_data", "lineno": 143, "message": "Downloading human_pro_classes.html,
https://sparql.proconsortium.org/virtuoso/sparql?query=PREFIX+obo%3A+%3Chttp%3A%2F%2Fpurl.obolibrary.org%2Fobo%2F%3E%0D%0A%0D%0ASELECT+%3FPRO_term%0D%0AFROM+%3Chttp%3A%2F%2Fpurl.obolibrary.org%2Fobo%2Fpr%3E%0D%0AWHERE+%7B%0D%0A+++++++%3FPRO
_term+rdf%3Atype+owl%3AClass+.%0D%0A+++++++%3FPRO_term+rdfs%3AsubClassOf+%3Frestriction+.%0D%0A+++++++%3Frestriction+owl%3AonProperty+obo%3ARO_0002160+.%0D%0A+++++++%3Frestriction+owl%3AsomeValuesFrom+obo%3ANCBITaxon_9606+.%0D%0A%0D%0A+++++++
%23+use+this+to+filter-out+things+like+hgnc+ids%0D%0A+++++++FILTER+%28regex%28%3FPRO_term%2C%22http%3A%2F%2Fpurl.obolibrary.org%2Fobo%2F*%22%29%29+.%0D%0A%7D&format=text%2Fhtml&debug="}
{"asctime": "2021-11-01 01:42:19,108", "levelname": "INFO", "name": "builds.build_phase_1", "module": "build_phase_1", "funcName": "downloads_build_data", "lineno": 143, "message": "Downloading variant_summary.txt,
ftp://ftp.ncbi.nlm.nih.gov/pub/clinvar/tab_delimited/variant_summary.txt.gz"}
{"asctime": "2021-11-01 01:42:34,238", "levelname": "INFO", "name": "builds.build_phase_1", "module": "build_phase_1", "funcName": "downloads_build_data", "lineno": 143, "message": "Downloading uniprot-cofactor-catalyst.tab,
https://www.uniprot.org/uniprot/?query=&fil=organism%3A%22Homo%20sapiens%20(Human)%20%5B9606%5D%22&columns=id%2Creviewed%2Centry%20name%2Cdatabase(PRO)%2Cchebi(Cofactor)%2Cchebi(Catalytic%20activity)&format=tab"}
{"asctime": "2021-11-01 01:43:24,015", "levelname": "INFO", "name": "builds.build_phase_1", "module": "build_phase_1", "funcName": "downloads_build_data", "lineno": 143, "message": "Downloading CTD_chem_gene_ixns.tsv, http://ctdbase.org/reports/CTD_chem_gene_ixns.tsv.gz"}
{"asctime": "2021-11-01 01:43:30,537", "levelname": "INFO", "name": "builds.build_phase_1", "module": "build_phase_1", "funcName": "downloads_build_data", "lineno": 143, "message": "Downloading CTD_chem_go_enriched.tsv, http://ctdbase.org/reports/CTD_chem_go_enriched.tsv.gz"}

{"asctime": "2021-11-01 01:43:55,405", "levelname": "INFO", "name": "builds.build_phase_1", "module": "build_phase_1", "funcName": "downloads_build_data", "lineno": 143, "message": "Downloading ChEBI2Reactome_All_Levels.txt, https://reactome.org/download/current/ChEBI2Reactome_All_Levels.txt"}
{"asctime": "2021-11-01 01:43:56,770", "levelname": "INFO", "name": "builds.build_phase_1", "module": "build_phase_1", "funcName": "downloads_build_data", "lineno": 143, "message": "Downloading CTD_chemicals_diseases.tsv, http://ctdbase.org/reports/CTD_chemicals_diseases.tsv.gz"}
{"asctime": "2021-11-01 01:44:18,538", "levelname": "INFO", "name": "builds.build_phase_1", "module": "build_phase_1", "funcName": "downloads_build_data", "lineno": 143, "message": "Downloading phenotype.hpoa, http://purl.obolibrary.org/obo/hp/hpoa/phenotype.hpoa"}
{"asctime": "2021-11-01 01:44:20,280", "levelname": "INFO", "name": "builds.build_phase_1", "module": "build_phase_1", "funcName": "downloads_build_data", "lineno": 143, "message": "Downloading curated_gene_disease_associations.tsv, https://www.disgenet.org/static/disgenet_ap1/files/downloads/curated_gene_disease_associations.tsv.gz"}
{"asctime": "2021-11-01 01:44:22,546", "levelname": "INFO", "name": "builds.build_phase_1", "module": "build_phase_1", "funcName": "downloads_build_data", "lineno": 143, "message": "Downloading COMBINED.DEFAULT_NETWORKS.BP_COMBINING.txt, http://genemania.org/data/current/Homo_sapiens.COMBINED/COMBINED.DEFAULT_NETWORKS.BP_COMBINING.txt"}
{"asctime": "2021-11-01 01:44:53,645", "levelname": "INFO", "name": "builds.build_phase_1", "module": "build_phase_1", "funcName": "downloads_build_data", "lineno": 143, "message": "Downloading CTD_genes_pathways.tsv, http://ctdbase.org/reports/CTD_genes_pathways.tsv.gz"}
{"asctime": "2021-11-01 01:44:54,405", "levelname": "INFO", "name": "builds.build_phase_1", "module": "build_phase_1", "funcName": "downloads_build_data", "lineno": 143, "message": "Downloading gene_association.reactome, https://reactome.org/download/current/gene_association.reactome.gz"}
{"asctime": "2021-11-01 01:44:55,295", "levelname": "INFO", "name": "builds.build_phase_1", "module": "build_phase_1", "funcName": "downloads_build_data", "lineno": 143, "message": "Downloading goa_human.gaf, http://current.geneontology.org/annotations/goa_human.gaf.gz"}
{"asctime": "2021-11-01 01:44:58,335", "levelname": "INFO", "name": "builds.build_phase_1", "module": "build_phase_1", "funcName": "downloads_build_data", "lineno": 143, "message": "Downloading UniProt2Reactome_All_Levels.txt, https://reactome.org/download/current/UniProt2Reactome_All_Levels.txt"}
{"asctime": "2021-11-01 01:45:01,370", "levelname": "INFO", "name": "builds.build_phase_1", "module": "build_phase_1", "funcName": "downloads_build_data", "lineno": 143, "message": "Downloading 9606.protein.links.v11.0.txt, https://stringdb-static.org/download/protein.links.v11.0/9606.protein.links.v11.0.txt.gz"}
{"asctime": "2021-11-01 01:45:14,415", "levelname": "INFO", "name": "builds.build_phase_1", "module": "build_phase_1", "funcName": "downloads_build_data", "lineno": 143, "message": "Downloading genomic_typing_dict.pkl, https://storage.googleapis.com/pheknowlator/curated_data/genomic_typing_dict.pkl"}
{"asctime": "2021-11-01 01:45:14,726", "levelname": "INFO", "name": "builds.build_phase_1", "module": "build_phase_1", "funcName": "downloads_build_data", "lineno": 143, "message": "Downloading zooma_tissue_cell_mapping_04JAN2020.xlsx, https://storage.googleapis.com/pheknowlator/curated_data/zooma_tissue_cell_mapping_04JAN2020.xlsx"}
{"asctime": "2021-11-01 01:45:15,900", "levelname": "INFO", "name": "__main__", "module": "phases1_2_entrypoint", "funcName": "main", "lineno": 52, "message": "#####BUILD PHASE 2: DATA PRE-PROCESSING#####"}
{"asctime": "2021-11-01 01:45:16,014", "levelname": "INFO", "name": "builds.data_preprocessing", "module": "data_preprocessing", "funcName": "preprocesses_build_data", "lineno": 1616, "message": "*** PROCESSING LINKED OPEN DATA SOURCES ***"}
{"asctime": "2021-11-01 01:45:16,015", "levelname": "INFO", "name": "builds.data_preprocessing", "module": "data_preprocessing", "funcName": "preprocesses_build_data", "lineno": 1619, "message": "STEP 1: HUMAN TRANSCRIPT, GENE, PROTEIN ID MAPPING"}
{"asctime": "2021-11-01 01:45:16,015", "levelname": "INFO", "name": "builds.data_preprocessing", "module": "data_preprocessing", "funcName": "generates_specific_genomic_identifier_maps", "lineno": 506, "message": "Generating Pairwise Genomic Cross-Map Sets"}
{"asctime": "2021-11-01 01:45:16,015", "levelname": "INFO", "name": "builds.data_preprocessing", "module": "data_preprocessing", "funcName": "_loads_genomic_typing_dictionary", "lineno": 114, "message": "Loading Genomic Typing Dictionary"}
{"asctime": "2021-11-01 01:45:16,075", "levelname": "INFO", "name": "builds.data_preprocessing", "module": "data_preprocessing", "funcName": "creates_master_genomic_identifier_map", "lineno": 469, "message": "Creating Genomic ID Cross-Map Dictionary"}
{"asctime": "2021-11-01 01:45:16,075", "levelname": "INFO", "name": "builds.data_preprocessing", "module": "data_preprocessing", "funcName": "_cross_maps_genomic_identifier_data", "lineno": 436, "message": "Cross-Mapping Genomic Identifier Data"}
{"asctime": "2021-11-01 01:45:16,076", "levelname": "INFO", "name": "builds.data_preprocessing", "module": "data_preprocessing", "funcName": "_fixes_genomic_symbols", "lineno": 406, "message": "Fixing genomic Symbols"}
{"asctime": "2021-11-01 01:45:16,076", "levelname": "INFO", "name": "builds.data_preprocessing", "module": "data_preprocessing", "funcName": "_merges_genomic_identifier_data", "lineno": 373, "message": "Merging Genomic ID Data"}
{"asctime": "2021-11-01 01:45:16,076", "levelname": "INFO", "name": "builds.data_preprocessing", "module": "data_preprocessing", "funcName": "_preprocess_hgnc_data", "lineno": 133, "message": "Preprocessing HGNC Data"}
{"asctime": "2021-11-01 01:45:40,677", "levelname": "INFO", "name": "builds.data_preprocessing", "module": "data_preprocessing", "funcName": "merges_ensembl_mapping_data", "lineno": 218, "message": "Merging Ensembl Annotation Data"}
{"asctime": "2021-11-01 01:45:43,385", "levelname": "INFO", "name": "builds.data_preprocessing", "module": "data_preprocessing", "funcName": "_preprocess_ensembl_data", "lineno": 176, "message": "Preprocessing Ensembl Data"}
{"asctime": "2021-11-01 01:47:36,552", "levelname": "INFO", "name": "builds.data_preprocessing", "module": "data_preprocessing", "funcName": "_preprocess_uniprot_data", "lineno": 268, "message": "Preprocessing UniProt Data"}
{"asctime": "2021-11-01 01:47:47,746", "levelname": "INFO", "name": "builds.data_preprocessing", "module": "data_preprocessing", "funcName": "_preprocess_ncbi_data", "lineno": 299, "message": "Preprocessing Entrez Data"}
{"asctime": "2021-11-01 01:48:45,188", "levelname": "INFO", "name": "builds.data_preprocessing", "module": "data_preprocessing", "funcName": "_preprocess_protein_ontology_mapping_data", "lineno": 352, "message": "Preprocessing Protein Ontology Data"}
{"asctime": "2021-11-01 02:29:55,647", "levelname": "INFO", "name": "builds.data_preprocessing", "module": "data_preprocessing", "funcName": "preprocesses_build_data", "lineno": 1624, "message": "STEP 2: MESH-CHEBI ID MAPPING"}
{"asctime": "2021-11-01 02:29:55,647", "levelname": "INFO", "name": "builds.data_preprocessing", "module": "data_preprocessing", "funcName": "creates_chebi_to_mesh_identifier_mappings", "lineno": 607, "message": "Creating MeSH-ChEBI ID Cross-Map Data"}
{"asctime": "2021-11-01 02:29:55,648", "levelname": "INFO", "name": "builds.data_preprocessing", "module": "data_preprocessing", "funcName": "_processes_mesh_data", "lineno": 543, "message": "Preprocessing MeSH Data"}
{"asctime": "2021-11-01 02:31:23,566", "levelname": "INFO", "name": "builds.data_preprocessing", "module": "data_preprocessing", "funcName": "_processes_chebi_data", "lineno": 585, "message": "Preprocessing ChEBI Data"}
{"asctime": "2021-11-01 02:31:30,646", "levelname": "INFO", "name": "builds.data_preprocessing", "module": "data_preprocessing", "funcName": "preprocesses_build_data", "lineno": 1629, "message": "STEP 3: DISEASE-PHENOTYPE ID MAPPING"}
{"asctime": "2021-11-01 02:31:30,646", "levelname": "INFO", "name": "builds.data_preprocessing", "module": "data_preprocessing", "funcName": "creates_disease_identifier_mappings", "lineno": 676, "message": "Creating Phenotype and Disease ID Cross-Map Data"}
{"asctime": "2021-11-01 02:31:30,646", "levelname": "INFO", "name": "builds.data_preprocessing", "module": "data_preprocessing", "funcName": "_preprocess_mondo_mapping_data", "lineno": 637, "message": "Loading MonDO Disease Ontology Data"}
{"asctime": "2021-11-01 02:38:35,522", "levelname": "INFO", "name": "builds.data_preprocessing", "module": "data_preprocessing", "funcName": "_preprocess_hpo_mapping_data", "lineno": 657, "message": "Loading Human Phenotype Ontology Data"}
{"asctime": "2021-11-01 02:42:04,165", "levelname": "INFO", "name": "builds.data_preprocessing", "module": "data_preprocessing", "funcName": "preprocesses_build_data", "lineno": 1634, "message": "STEP 4: CREATING HPA + GTEX ID EDGE DATA"}
{"asctime": "2021-11-01 02:42:04,165", "levelname": "INFO", "name": "builds.data_preprocessing", "module": "data_preprocessing", "funcName": "_hpa_gtex_ontology_alignment", "lineno": 730, "message": "Preprocessing HPA Data"}
{"asctime": "2021-11-01 02:42:06,905", "levelname": "INFO", "name": "builds.data_preprocessing", "module": "data_preprocessing", "funcName": "processes_hpa_gtex_data", "lineno": 781, "message": "Creating Human Protein Atlas and GTEx Cross-Map Data"}
{"asctime": "2021-11-01 02:42:06,906", "levelname": "INFO", "name": "builds.data_preprocessing", "module": "data_preprocessing", "funcName": "_extracts_hpa_tissue_information", "lineno": 754, "message": "Extracting HPA Tissue and Cell Information"}
{"asctime": "2021-11-01 02:43:51,974", "levelname": "INFO", "name": "builds.data_preprocessing", "module": "data_preprocessing", "funcName": "preprocesses_build_data", "lineno": 1640, "message": "STEP 5: SEQUENCE ONTOLOGY + PATHWAY ID MAP"}
{"asctime": "2021-11-01 02:43:51,974", "levelname": "INFO", "name": "builds.data_preprocessing", "module": "data_preprocessing", "funcName": "combines_pathway_and_sequence_ontology_dictionaries", "lineno": 1112, "message": "Creating Pathway and Sequence Ontology Mapping Dictionary"}
{"asctime": "2021-11-01 02:43:51,975", "levelname": "INFO", "name": "builds.data_preprocessing", "module": "data_preprocessing", "funcName": "_creates_sequence_identifier_mappings", "lineno": 1084, "message": "Creating Sequence Ontology ID Cross-Map Data"}
{"asctime": "2021-11-01 02:43:52,107", "levelname": "INFO", "name": "builds.data_preprocessing", "module": "data_preprocessing", "funcName": "_preprocesses_gene_types", "lineno": 993, "message": "Mapping Sequence Ontology Classes to Gene IDs"}
{"asctime": "2021-11-01 02:44:06,476", "levelname": "INFO", "name": "builds.data_preprocessing", "module": "data_preprocessing", "funcName": "_preprocesses_transcript_types", "lineno": 1028, "message": "Mapping Sequence Ontology Classes to Transcript IDs"}
{"asctime": "2021-11-01 02:44:58,316", "levelname": "INFO", "name": "builds.data_preprocessing", "module": "data_preprocessing", "funcName": "_preprocesses_variant_types", "lineno": 1059, "message": "Mapping Sequence Ontology Classes to Variant IDs"}
{"asctime": "2021-11-01 02:51:39,102", "levelname": "INFO", "name": "builds.data_preprocessing", "module": "data_preprocessing", "funcName": "_creates_pathway_identifier_mappings", "lineno": 964, "message": "Creating Pathway Ontology ID Cross-Map Data"}
{"asctime": "2021-11-01 02:51:39,102", "levelname": "INFO", "name": "builds.data_preprocessing", "module": "data_preprocessing", "funcName": "_preprocess_pathway_mapping_data", "lineno": 831, "message": "Loading Protein Ontology Data"}
{"asctime": "2021-11-01 02:51:46,164", "levelname": "INFO", "name": "builds.data_preprocessing", "module": "data_preprocessing", "funcName": "_processes_reactome_data", "lineno": 852, "message": "Loading Reactome Annotation Data"}
{"asctime": "2021-11-01 02:51:47,090", "levelname": "INFO", "name": "builds.data_preprocessing", "module": "data_preprocessing", "funcName": "_processes_compath_pathway_data", "lineno": 882, "message": "Loading ComPath Canonical Pathway Data"}
{"asctime": "2021-11-01 02:51:47,482", "levelname": "INFO", "name": "builds.data_preprocessing", "module": "data_preprocessing", "funcName": "_processes_kegg_pathway_data", "lineno": 911, "message": "Loading KEGG Data"}
{"asctime": "2021-11-01 02:51:47,671", "levelname": "INFO", "name": "builds.data_preprocessing", "module": "data_preprocessing", "funcName": "_queries_reactome_api", "lineno": 940, "message": "Querying Reactome API for Reactome-GO BP Mappings"}
{"asctime": "2021-11-01 02:56:50,953", "levelname": "INFO", "name": "builds.data_preprocessing", "module": "data_preprocessing", "funcName": "preprocesses_build_data", "lineno": 1645, "message": "STEP 6: CREATING A HUMAN PROTEIN ONTOLOGY"}
{"asctime": "2021-11-01 02:56:50,954", "levelname": "INFO", "name": "builds.data_preprocessing", "module": "data_preprocessing", "funcName": "constructs_human_protein_ontology", "lineno": 1202, "message": "Construct a Human PRotein Ontology"}
{"asctime": "2021-11-01 02:56:50,954", "levelname": "INFO", "name": "builds.data_preprocessing", "module": "data_preprocessing", "funcName": "_processes_protein_ontology_data", "lineno": 1134, "message": "Loading Protein Ontology Data"}
{"asctime": "2021-11-01 04:31:21,804", "levelname": "INFO", "name": "builds.data_preprocessing", "module": "data_preprocessing", "funcName": "_logically_verifies_human_protein_ontology", "lineno": 1172, "message": "Logically Verifying Human Protein Ontology Subset"}
{"asctime": "2021-11-01 04:34:16,112", "levelname": "INFO", "name": "builds.data_preprocessing", "module": "data_preprocessing", "funcName": "preprocesses_build_data", "lineno": 1650, "message": "STEP 7: EXTRACTING RELATION ONTOLOGY INFO"}
{"asctime": "2021-11-01 04:34:16,112", "levelname": "INFO", "name": "builds.data_preprocessing", "module": "data_preprocessing", "funcName": "processes_relation_ontology_data", "lineno": 1237, "message": "Creating Required Relations Ontology Data"}
{"asctime": "2021-11-01 04:34:17,955", "levelname": "INFO", "name": "builds.data_preprocessing", "module": "data_preprocessing", "funcName": "preprocesses_build_data", "lineno": 1655, "message": "STEP 8: CREATING CLINVAR VARIANT-DISEASE-PHENOTYPE DATA"}
{"asctime": "2021-11-01 04:34:17,955", "levelname": "INFO", "name": "builds.data_preprocessing", "module": "data_preprocessing", "funcName": "processes_clinvar_data", "lineno": 1270, "message": "Generating ClinVar Cross-Mapping Data"}
{"asctime": "2021-11-01 04:41:16,913", "levelname": "INFO", "name": "builds.data_preprocessing", "module": "data_preprocessing", "funcName": "preprocesses_build_data", "lineno": 1660, "message": "STEP 9: CREATING COFACTOR + CATALYST EDGE DATA"}
{"asctime": "2021-11-01 04:41:16,913", "levelname": "INFO", "name": "builds.data_preprocessing", "module": "data_preprocessing", "funcName": "processes_cofactor_catalyst_data", "lineno": 1294, "message": "Creating Protein-Cofactor and Protein-Catalyst Cross-Mappings"}
{"asctime": "2021-11-01 04:41:18,013", "levelname": "INFO", "name": "builds.data_preprocessing", "module": "data_preprocessing", "funcName": "preprocesses_build_data", "lineno": 1665, "message": "STEP 10: CREATING OBO-ONTOLOGY METADATA DICTIONARY"}

{"asctime": "2021-11-01 04:41:18,013", "levelname": "INFO", "name": "builds.data_preprocessing", "module": "data_preprocessing", "funcName": "creates_non_ontology_class_metadata_dict", "lineno": 1582, "message": "Creating Master Metadata Dictionary for Non-Ontology Entities"}
{"asctime": "2021-11-01 04:41:18,013", "levelname": "INFO", "name": "builds.data_preprocessing", "module": "data_preprocessing", "funcName": "_creates_gene_metadata_dict", "lineno": 1330, "message": "Generating Metadata for Gene Identifiers"}
{"asctime": "2021-11-01 04:41:39,478", "levelname": "INFO", "name": "builds.data_preprocessing", "module": "data_preprocessing", "funcName": "_creates_transcript_metadata_dict", "lineno": 1375, "message": "Generating Metadata for Transcript Identifiers"}
{"asctime": "2021-11-01 04:42:28,420", "levelname": "INFO", "name": "builds.data_preprocessing", "module": "data_preprocessing", "funcName": "_creates_variant_metadata_dict", "lineno": 1418, "message": "Generating Metadata for Variant IDs"}
{"asctime": "2021-11-01 04:46:58,371", "levelname": "INFO", "name": "builds.data_preprocessing", "module": "data_preprocessing", "funcName": "_creates_pathway_metadata_dict", "lineno": 1500, "message": "Generating Metadata for Pathway IDs"}
{"asctime": "2021-11-01 04:52:02,207", "levelname": "INFO", "name": "builds.data_preprocessing", "module": "data_preprocessing", "funcName": "_creates_relations_metadata_dict", "lineno": 1541, "message": "Generating Metadata for Relations IDs"}
{"asctime": "2021-11-01 04:52:37,865", "levelname": "INFO", "name": "builds.ontology_cleaning", "module": "ontology_cleaning", "funcName": "cleans_ontology_data", "lineno": 577, "message": "*** CLEANING INDIVIDUAL ONTOLOGY DATA SOURCES ***"}
{"asctime": "2021-11-01 04:52:37,866", "levelname": "INFO", "name": "builds.ontology_cleaning", "module": "ontology_cleaning", "funcName": "cleans_ontology_data", "lineno": 582, "message": "\nProcessing Ontology: CHEBI_WITH_IMPORTS.OWL"}
{"asctime": "2021-11-01 05:09:39,392", "levelname": "INFO", "name": "builds.ontology_cleaning", "module": "ontology_cleaning", "funcName": "updates_ontology_reporter", "lineno": 201, "message": "Obtaining Ontology Statistics"}
{"asctime": "2021-11-01 05:20:44,620", "levelname": "INFO", "name": "builds.ontology_cleaning", "module": "ontology_cleaning", "funcName": "fixes_ontology_parsing_errors", "lineno": 253, "message": "Finding Parsing Errors"}
{"asctime": "2021-11-01 05:22:09,099", "levelname": "INFO", "name": "builds.ontology_cleaning", "module": "ontology_cleaning", "funcName": "fixes_identifier_errors", "lineno": 278, "message": "Fixing Identifier Errors"}
{"asctime": "2021-11-01 05:23:00,468", "levelname": "INFO", "name": "builds.ontology_cleaning", "module": "ontology_cleaning", "funcName": "removes_deprecated_obsolete_entities", "lineno": 332, "message": "Removing Deprecated and Obsolete Classes"}
{"asctime": "2021-11-01 05:23:47,669", "levelname": "INFO", "name": "builds.ontology_cleaning", "module": "ontology_cleaning", "funcName": "fixes_punning_errors", "lineno": 362, "message": "Resolving Punning Errors"}
{"asctime": "2021-11-01 05:24:57,896", "levelname": "INFO", "name": "builds.ontology_cleaning", "module": "ontology_cleaning", "funcName": "_logically_verifies_cleaned_ontologies", "lineno": 178, "message": "PKT: Logically Verifying Ontology"}
{"asctime": "2021-11-01 05:30:47,755", "levelname": "INFO", "name": "builds.ontology_cleaning", "module": "ontology_cleaning", "funcName": "cleans_ontology_data", "lineno": 592, "message": "Reading in Cleaned Ontology -- Needed to Calculate Final Statistics"}
{"asctime": "2021-11-01 05:47:36,351", "levelname": "INFO", "name": "builds.ontology_cleaning", "module": "ontology_cleaning", "funcName": "updates_ontology_reporter", "lineno": 201, "message": "Obtaining Ontology Statistics"}
{"asctime": "2021-11-01 05:58:44,280", "levelname": "INFO", "name": "builds.ontology_cleaning", "module": "ontology_cleaning", "funcName": "cleans_ontology_data", "lineno": 582, "message": "\nProcessing Ontology: CLO_WITH_IMPORTS.OWL"}
{"asctime": "2021-11-01 06:02:04,699", "levelname": "INFO", "name": "builds.ontology_cleaning", "module": "ontology_cleaning", "funcName": "updates_ontology_reporter", "lineno": 201, "message": "Obtaining Ontology Statistics"}
{"asctime": "2021-11-01 06:04:33,690", "levelname": "INFO", "name": "builds.ontology_cleaning", "module": "ontology_cleaning", "funcName": "fixes_ontology_parsing_errors", "lineno": 253, "message": "Finding Parsing Errors"}
{"asctime": "2021-11-01 06:04:46,221", "levelname": "INFO", "name": "builds.ontology_cleaning", "module": "ontology_cleaning", "funcName": "fixes_identifier_errors", "lineno": 278, "message": "Fixing Identifier Errors"}
{"asctime": "2021-11-01 06:04:59,128", "levelname": "INFO", "name": "builds.ontology_cleaning", "module": "ontology_cleaning", "funcName": "removes_deprecated_obsolete_entities", "lineno": 332, "message": "Removing Deprecated and Obsolete Classes"}
{"asctime": "2021-11-01 06:05:09,062", "levelname": "INFO", "name": "builds.ontology_cleaning", "module": "ontology_cleaning", "funcName": "fixes_punning_errors", "lineno": 362, "message": "Resolving Punning Errors"}
{"asctime": "2021-11-01 06:05:32,673", "levelname": "INFO", "name": "builds.ontology_cleaning", "module": "ontology_cleaning", "funcName": "_logically_verifies_cleaned_ontologies", "lineno": 178, "message": "PKT: Logically Verifying Ontology"}
{"asctime": "2021-11-01 06:07:15,029", "levelname": "INFO", "name": "builds.ontology_cleaning", "module": "ontology_cleaning", "funcName": "cleans_ontology_data", "lineno": 592, "message": "Reading in Cleaned Ontology -- Needed to Calculate Final Statistics"}
{"asctime": "2021-11-01 06:10:43,709", "levelname": "INFO", "name": "builds.ontology_cleaning", "module": "ontology_cleaning", "funcName": "updates_ontology_reporter", "lineno": 201, "message": "Obtaining Ontology Statistics"}
{"asctime": "2021-11-01 06:13:10,116", "levelname": "INFO", "name": "builds.ontology_cleaning", "module": "ontology_cleaning", "funcName": "cleans_ontology_data", "lineno": 582, "message": "\nProcessing Ontology: EXT_WITH_IMPORTS.OWL"}
{"asctime": "2021-11-01 06:15:09,658", "levelname": "INFO", "name": "builds.ontology_cleaning", "module": "ontology_cleaning", "funcName": "updates_ontology_reporter", "lineno": 201, "message": "Obtaining Ontology Statistics"}
{"asctime": "2021-11-01 06:16:26,494", "levelname": "INFO", "name": "builds.ontology_cleaning", "module": "ontology_cleaning", "funcName": "fixes_ontology_parsing_errors", "lineno": 253, "message": "Finding Parsing Errors"}
{"asctime": "2021-11-01 06:16:39,857", "levelname": "INFO", "name": "builds.ontology_cleaning", "module": "ontology_cleaning", "funcName": "fixes_identifier_errors", "lineno": 278, "message": "Fixing Identifier Errors"}
{"asctime": "2021-11-01 06:16:47,098", "levelname": "INFO", "name": "builds.ontology_cleaning", "module": "ontology_cleaning", "funcName": "removes_deprecated_obsolete_entities", "lineno": 332, "message": "Removing Deprecated and Obsolete Classes"}
{"asctime": "2021-11-01 06:16:54,469", "levelname": "INFO", "name": "builds.ontology_cleaning", "module": "ontology_cleaning", "funcName": "fixes_punning_errors", "lineno": 362, "message": "Resolving Punning Errors"}
{"asctime": "2021-11-01 06:17:05,375", "levelname": "INFO", "name": "builds.ontology_cleaning", "module": "ontology_cleaning", "funcName": "_logically_verifies_cleaned_ontologies", "lineno": 178, "message": "PKT: Logically Verifying Ontology"}
{"asctime": "2021-11-01 06:17:59,074", "levelname": "INFO", "name": "builds.ontology_cleaning", "module": "ontology_cleaning", "funcName": "cleans_ontology_data", "lineno": 592, "message": "Reading in Cleaned Ontology -- Needed to Calculate Final Statistics"}
{"asctime": "2021-11-01 06:19:57,152", "levelname": "INFO", "name": "builds.ontology_cleaning", "module": "ontology_cleaning", "funcName": "updates_ontology_reporter", "lineno": 201, "message": "Obtaining Ontology Statistics"}
{"asctime": "2021-11-01 06:21:12,617", "levelname": "INFO", "name": "builds.ontology_cleaning", "module": "ontology_cleaning", "funcName": "cleans_ontology_data", "lineno": 582, "message": "\nProcessing Ontology: GO_WITH_IMPORTS.OWL"}
{"asctime": "2021-11-01 06:24:56,179", "levelname": "INFO", "name": "builds.ontology_cleaning", "module": "ontology_cleaning", "funcName": "updates_ontology_reporter", "lineno": 201, "message": "Obtaining Ontology Statistics"}
{"asctime": "2021-11-01 06:27:23,258", "levelname": "INFO", "name": "builds.ontology_cleaning", "module": "ontology_cleaning", "funcName": "fixes_ontology_parsing_errors", "lineno": 253, "message": "Finding Parsing Errors"}
{"asctime": "2021-11-01 06:27:46,878", "levelname": "INFO", "name": "builds.ontology_cleaning", "module": "ontology_cleaning", "funcName": "fixes_identifier_errors", "lineno": 278, "message": "Fixing Identifier Errors"}
{"asctime": "2021-11-01 06:28:00,501", "levelname": "INFO", "name": "builds.ontology_cleaning", "module": "ontology_cleaning", "funcName": "removes_deprecated_obsolete_entities", "lineno": 332, "message": "Removing Deprecated and Obsolete Classes"}
{"asctime": "2021-11-01 06:28:23,759", "levelname": "INFO", "name": "builds.ontology_cleaning", "module": "ontology_cleaning", "funcName": "fixes_punning_errors", "lineno": 362, "message": "Resolving Punning Errors"}
{"asctime": "2021-11-01 06:28:42,579", "levelname": "INFO", "name": "builds.ontology_cleaning", "module": "ontology_cleaning", "funcName": "_logically_verifies_cleaned_ontologies", "lineno": 178, "message": "PKT: Logically Verifying Ontology"}
{"asctime": "2021-11-01 06:30:12,230", "levelname": "INFO", "name": "builds.ontology_cleaning", "module": "ontology_cleaning", "funcName": "cleans_ontology_data", "lineno": 592, "message": "Reading in Cleaned Ontology -- Needed to Calculate Final Statistics"}
{"asctime": "2021-11-01 06:33:40,575", "levelname": "INFO", "name": "builds.ontology_cleaning", "module": "ontology_cleaning", "funcName": "updates_ontology_reporter", "lineno": 201, "message": "Obtaining Ontology Statistics"}
{"asctime": "2021-11-01 06:36:01,584", "levelname": "INFO", "name": "builds.ontology_cleaning", "module": "ontology_cleaning", "funcName": "cleans_ontology_data", "lineno": 582, "message": "\nProcessing Ontology: HP_WITH_IMPORTS.OWL"}
{"asctime": "2021-11-01 06:38:29,033", "levelname": "INFO", "name": "builds.ontology_cleaning", "module": "ontology_cleaning", "funcName": "updates_ontology_reporter", "lineno": 201, "message": "Obtaining Ontology Statistics"}
{"asctime": "2021-11-01 06:40:09,978", "levelname": "INFO", "name": "builds.ontology_cleaning", "module": "ontology_cleaning", "funcName": "fixes_ontology_parsing_errors", "lineno": 253, "message": "Finding Parsing Errors"}
{"asctime": "2021-11-01 06:40:25,302", "levelname": "INFO", "name": "builds.ontology_cleaning", "module": "ontology_cleaning", "funcName": "fixes_identifier_errors", "lineno": 278, "message": "Fixing Identifier Errors"}
{"asctime": "2021-11-01 06:40:34,105", "levelname": "INFO", "name": "builds.ontology_cleaning", "module": "ontology_cleaning", "funcName": "removes_deprecated_obsolete_entities", "lineno": 332, "message": "Removing Deprecated and Obsolete Classes"}
{"asctime": "2021-11-01 06:40:41,258", "levelname": "INFO", "name": "builds.ontology_cleaning", "module": "ontology_cleaning", "funcName": "fixes_punning_errors", "lineno": 362, "message": "Resolving Punning Errors"}
{"asctime": "2021-11-01 06:40:55,062", "levelname": "INFO", "name": "builds.ontology_cleaning", "module": "ontology_cleaning", "funcName": "_logically_verifies_cleaned_ontologies", "lineno": 178, "message": "PKT: Logically Verifying Ontology"}
{"asctime": "2021-11-01 06:42:02,123", "levelname": "INFO", "name": "builds.ontology_cleaning", "module": "ontology_cleaning", "funcName": "cleans_ontology_data", "lineno": 592, "message": "Reading in Cleaned Ontology -- Needed to Calculate Final Statistics"}
{"asctime": "2021-11-01 06:44:32,077", "levelname": "INFO", "name": "builds.ontology_cleaning", "module": "ontology_cleaning", "funcName": "updates_ontology_reporter", "lineno": 201, "message": "Obtaining Ontology Statistics"}
{"asctime": "2021-11-01 06:46:13,544", "levelname": "INFO", "name": "builds.ontology_cleaning", "module": "ontology_cleaning", "funcName": "cleans_ontology_data", "lineno": 582, "message": "\nProcessing Ontology: MONDO_WITH_IMPORTS.OWL"}
{"asctime": "2021-11-01 06:52:57,054", "levelname": "INFO", "name": "builds.ontology_cleaning", "module": "ontology_cleaning", "funcName": "updates_ontology_reporter", "lineno": 201, "message": "Obtaining Ontology Statistics"}
{"asctime": "2021-11-01 06:57:17,692", "levelname": "INFO", "name": "builds.ontology_cleaning", "module": "ontology_cleaning", "funcName": "fixes_ontology_parsing_errors", "lineno": 253, "message": "Finding Parsing Errors"}
{"asctime": "2021-11-01 06:57:59,960", "levelname": "INFO", "name": "builds.ontology_cleaning", "module": "ontology_cleaning", "funcName": "fixes_identifier_errors", "lineno": 278, "message": "Fixing Identifier Errors"}
{"asctime": "2021-11-01 06:58:22,654", "levelname": "INFO", "name": "builds.ontology_cleaning", "module": "ontology_cleaning", "funcName": "removes_deprecated_obsolete_entities", "lineno": 332, "message": "Removing Deprecated and Obsolete Classes"}
{"asctime": "2021-11-01 06:58:45,214", "levelname": "INFO", "name": "builds.ontology_cleaning", "module": "ontology_cleaning", "funcName": "fixes_punning_errors", "lineno": 362, "message": "Resolving Punning Errors"}
{"asctime": "2021-11-01 06:59:18,777", "levelname": "INFO", "name": "builds.ontology_cleaning", "module": "ontology_cleaning", "funcName": "_logically_verifies_cleaned_ontologies", "lineno": 178, "message": "PKT: Logically Verifying Ontology"}
{"asctime": "2021-11-01 07:01:56,990", "levelname": "INFO", "name": "builds.ontology_cleaning", "module": "ontology_cleaning", "funcName": "cleans_ontology_data", "lineno": 592, "message": "Reading in Cleaned Ontology -- Needed to Calculate Final Statistics"}
{"asctime": "2021-11-01 07:08:40,760", "levelname": "INFO", "name": "builds.ontology_cleaning", "module": "ontology_cleaning", "funcName": "updates_ontology_reporter", "lineno": 201, "message": "Obtaining Ontology Statistics"}
{"asctime": "2021-11-01 07:13:09,817", "levelname": "INFO", "name": "builds.ontology_cleaning", "module": "ontology_cleaning", "funcName": "cleans_ontology_data", "lineno": 582, "message": "\nProcessing Ontology: PR_WITH_IMPORTS.OWL"}
{"asctime": "2021-11-01 07:19:05,834", "levelname": "INFO", "name": "builds.ontology_cleaning", "module": "ontology_cleaning", "funcName": "updates_ontology_reporter", "lineno": 201, "message": "Obtaining Ontology Statistics"}
{"asctime": "2021-11-01 07:23:36,231", "levelname": "INFO", "name": "builds.ontology_cleaning", "module": "ontology_cleaning", "funcName": "fixes_ontology_parsing_errors", "lineno": 253, "message": "Finding Parsing Errors"}
{"asctime": "2021-11-01 07:24:09,599", "levelname": "INFO", "name": "builds.ontology_cleaning", "module": "ontology_cleaning", "funcName": "fixes_identifier_errors", "lineno": 278, "message": "Fixing Identifier Errors"}
{"asctime": "2021-11-01 07:24:31,041", "levelname": "INFO", "name": "builds.ontology_cleaning", "module": "ontology_cleaning", "funcName": "removes_deprecated_obsolete_entities", "lineno": 332, "message": "Removing Deprecated and Obsolete Classes"}
{"asctime": "2021-11-01 07:24:47,202", "levelname": "INFO", "name": "builds.ontology_cleaning", "module": "ontology_cleaning", "funcName": "fixes_punning_errors", "lineno": 362, "message": "Resolving Punning Errors"}
{"asctime": "2021-11-01 07:25:18,307", "levelname": "INFO", "name": "builds.ontology_cleaning", "module": "ontology_cleaning", "funcName": "_logically_verifies_cleaned_ontologies", "lineno": 178, "message": "PKT: Logically Verifying Ontology"}
{"asctime": "2021-11-01 07:27:46,337", "levelname": "INFO", "name": "builds.ontology_cleaning", "module": "ontology_cleaning", "funcName": "cleans_ontology_data", "lineno": 592, "message": "Reading in Cleaned Ontology -- Needed to Calculate Final Statistics"}
{"asctime": "2021-11-01 07:33:46,590", "levelname": "INFO", "name": "builds.ontology_cleaning", "module": "ontology_cleaning", "funcName": "updates_ontology_reporter", "lineno": 201, "message": "Obtaining Ontology Statistics"}
{"asctime": "2021-11-01 07:38:14,914", "levelname": "INFO", "name": "builds.ontology_cleaning", "module": "ontology_cleaning", "funcName": "cleans_ontology_data", "lineno": 582, "message": "\nProcessing Ontology: PW_WITH_IMPORTS.OWL"}

{"asctime": "2021-11-01 07:38:21,747", "levelname": "INFO", "name": "builds.ontology_cleaning", "module": "ontology_cleaning", "funcName": "updates_ontology_reporter", "lineno": 201, "message": "Obtaining Ontology Statistics"}
{"asctime": "2021-11-01 07:38:26,004", "levelname": "INFO", "name": "builds.ontology_cleaning", "module": "ontology_cleaning", "funcName": "fixes_ontology_parsing_errors", "lineno": 253, "message": "Finding Parsing Errors"}
{"asctime": "2021-11-01 07:38:28,882", "levelname": "INFO", "name": "builds.ontology_cleaning", "module": "ontology_cleaning", "funcName": "fixes_identifier_errors", "lineno": 278, "message": "Fixing Identifier Errors"}
{"asctime": "2021-11-01 07:38:29,205", "levelname": "INFO", "name": "builds.ontology_cleaning", "module": "ontology_cleaning", "funcName": "removes_deprecated_obsolete_entities", "lineno": 332, "message": "Removing Deprecated and Obsolete Classes"}
{"asctime": "2021-11-01 07:38:29,488", "levelname": "INFO", "name": "builds.ontology_cleaning", "module": "ontology_cleaning", "funcName": "fixes_punning_errors", "lineno": 362, "message": "Resolving Punning Errors"}
{"asctime": "2021-11-01 07:38:29,933", "levelname": "INFO", "name": "builds.ontology_cleaning", "module": "ontology_cleaning", "funcName": "_logically_verifies_cleaned_ontologies", "lineno": 178, "message": "PKT: Logically Verifying Ontology"}
{"asctime": "2021-11-01 07:38:35,443", "levelname": "INFO", "name": "builds.ontology_cleaning", "module": "ontology_cleaning", "funcName": "cleans_ontology_data", "lineno": 592, "message": "Reading in Cleaned Ontology -- Needed to Calculate Final Statistics"}
{"asctime": "2021-11-01 07:38:41,903", "levelname": "INFO", "name": "builds.ontology_cleaning", "module": "ontology_cleaning", "funcName": "updates_ontology_reporter", "lineno": 201, "message": "Obtaining Ontology Statistics"}
{"asctime": "2021-11-01 07:38:46,242", "levelname": "INFO", "name": "builds.ontology_cleaning", "module": "ontology_cleaning", "funcName": "cleans_ontology_data", "lineno": 582, "message": "\nProcessing Ontology: RO_WITH_IMPORTS.OWL"}
{"asctime": "2021-11-01 07:38:47,661", "levelname": "INFO", "name": "builds.ontology_cleaning", "module": "ontology_cleaning", "funcName": "updates_ontology_reporter", "lineno": 201, "message": "Obtaining Ontology Statistics"}
{"asctime": "2021-11-01 07:38:48,418", "levelname": "INFO", "name": "builds.ontology_cleaning", "module": "ontology_cleaning", "funcName": "fixes_ontology_parsing_errors", "lineno": 253, "message": "Finding Parsing Errors"}
{"asctime": "2021-11-01 07:38:50,807", "levelname": "INFO", "name": "builds.ontology_cleaning", "module": "ontology_cleaning", "funcName": "fixes_identifier_errors", "lineno": 278, "message": "Fixing Identifier Errors"}
{"asctime": "2021-11-01 07:38:50,872", "levelname": "INFO", "name": "builds.ontology_cleaning", "module": "ontology_cleaning", "funcName": "removes_deprecated_obsolete_entities", "lineno": 332, "message": "Removing Deprecated and Obsolete Classes"}
{"asctime": "2021-11-01 07:38:50,935", "levelname": "INFO", "name": "builds.ontology_cleaning", "module": "ontology_cleaning", "funcName": "fixes_punning_errors", "lineno": 362, "message": "Resolving Punning Errors"}
{"asctime": "2021-11-01 07:38:51,053", "levelname": "INFO", "name": "builds.ontology_cleaning", "module": "ontology_cleaning", "funcName": "_logically_verifies_cleaned_ontologies", "lineno": 178, "message": "PKT: Logically Verifying Ontology"}
{"asctime": "2021-11-01 07:38:54,246", "levelname": "INFO", "name": "builds.ontology_cleaning", "module": "ontology_cleaning", "funcName": "cleans_ontology_data", "lineno": 592, "message": "Reading in Cleaned Ontology -- Needed to Calculate Final Statistics"}
{"asctime": "2021-11-01 07:38:55,667", "levelname": "INFO", "name": "builds.ontology_cleaning", "module": "ontology_cleaning", "funcName": "updates_ontology_reporter", "lineno": 201, "message": "Obtaining Ontology Statistics"}
{"asctime": "2021-11-01 07:38:56,574", "levelname": "INFO", "name": "builds.ontology_cleaning", "module": "ontology_cleaning", "funcName": "cleans_ontology_data", "lineno": 582, "message": "\nProcessing Ontology: SO_WITH_IMPORTS.OWL"}
{"asctime": "2021-11-01 07:39:04,809", "levelname": "INFO", "name": "builds.ontology_cleaning", "module": "ontology_cleaning", "funcName": "updates_ontology_reporter", "lineno": 201, "message": "Obtaining Ontology Statistics"}
{"asctime": "2021-11-01 07:39:16,320", "levelname": "INFO", "name": "builds.ontology_cleaning", "module": "ontology_cleaning", "funcName": "fixes_ontology_parsing_errors", "lineno": 253, "message": "Finding Parsing Errors"}
{"asctime": "2021-11-01 07:39:19,403", "levelname": "INFO", "name": "builds.ontology_cleaning", "module": "ontology_cleaning", "funcName": "fixes_identifier_errors", "lineno": 278, "message": "Fixing Identifier Errors"}
{"asctime": "2021-11-01 07:39:19,809", "levelname": "INFO", "name": "builds.ontology_cleaning", "module": "ontology_cleaning", "funcName": "removes_deprecated_obsolete_entities", "lineno": 332, "message": "Removing Deprecated and Obsolete Classes"}
{"asctime": "2021-11-01 07:39:20,357", "levelname": "INFO", "name": "builds.ontology_cleaning", "module": "ontology_cleaning", "funcName": "fixes_punning_errors", "lineno": 362, "message": "Resolving Punning Errors"}
{"asctime": "2021-11-01 07:39:20,914", "levelname": "INFO", "name": "builds.ontology_cleaning", "module": "ontology_cleaning", "funcName": "_logically_verifies_cleaned_ontologies", "lineno": 178, "message": "PKT: Logically Verifying Ontology"}
{"asctime": "2021-11-01 07:39:27,169", "levelname": "INFO", "name": "builds.ontology_cleaning", "module": "ontology_cleaning", "funcName": "cleans_ontology_data", "lineno": 592, "message": "Reading in Cleaned Ontology -- Needed to Calculate Final Statistics"}
{"asctime": "2021-11-01 07:39:34,919", "levelname": "INFO", "name": "builds.ontology_cleaning", "module": "ontology_cleaning", "funcName": "updates_ontology_reporter", "lineno": 201, "message": "Obtaining Ontology Statistics"}
{"asctime": "2021-11-01 07:39:39,877", "levelname": "INFO", "name": "builds.ontology_cleaning", "module": "ontology_cleaning", "funcName": "cleans_ontology_data", "lineno": 582, "message": "\nProcessing Ontology: VO_WITH_IMPORTS.OWL"}
{"asctime": "2021-11-01 07:39:55,084", "levelname": "INFO", "name": "builds.ontology_cleaning", "module": "ontology_cleaning", "funcName": "updates_ontology_reporter", "lineno": 201, "message": "Obtaining Ontology Statistics"}
{"asctime": "2021-11-01 07:40:04,778", "levelname": "INFO", "name": "builds.ontology_cleaning", "module": "ontology_cleaning", "funcName": "fixes_ontology_parsing_errors", "lineno": 253, "message": "Finding Parsing Errors"}
{"asctime": "2021-11-01 07:40:08,518", "levelname": "INFO", "name": "builds.ontology_cleaning", "module": "ontology_cleaning", "funcName": "fixes_identifier_errors", "lineno": 278, "message": "Fixing Identifier Errors"}
{"asctime": "2021-11-01 07:40:09,438", "levelname": "INFO", "name": "builds.ontology_cleaning", "module": "ontology_cleaning", "funcName": "removes_deprecated_obsolete_entities", "lineno": 332, "message": "Removing Deprecated and Obsolete Classes"}
{"asctime": "2021-11-01 07:40:10,082", "levelname": "INFO", "name": "builds.ontology_cleaning", "module": "ontology_cleaning", "funcName": "fixes_punning_errors", "lineno": 362, "message": "Resolving Punning Errors"}
{"asctime": "2021-11-01 07:40:11,394", "levelname": "INFO", "name": "builds.ontology_cleaning", "module": "ontology_cleaning", "funcName": "_logically_verifies_cleaned_ontologies", "lineno": 178, "message": "PKT: Logically Verifying Ontology"}
{"asctime": "2021-11-01 07:40:22,176", "levelname": "INFO", "name": "builds.ontology_cleaning", "module": "ontology_cleaning", "funcName": "cleans_ontology_data", "lineno": 592, "message": "Reading in Cleaned Ontology -- Needed to Calculate Final Statistics"}
{"asctime": "2021-11-01 07:40:37,907", "levelname": "INFO", "name": "builds.ontology_cleaning", "module": "ontology_cleaning", "funcName": "updates_ontology_reporter", "lineno": 201, "message": "Obtaining Ontology Statistics"}
{"asctime": "2021-11-01 07:40:48,694", "levelname": "INFO", "name": "builds.ontology_cleaning", "module": "ontology_cleaning", "funcName": "cleans_ontology_data", "lineno": 598, "message": "*** CLEANING MERGED ONTOLOGY DATA ***"}
{"asctime": "2021-11-01 07:40:48,695", "levelname": "INFO", "name": "builds.ontology_cleaning", "module": "ontology_cleaning", "funcName": "merge_ontologies", "lineno": 161, "message": "Merging Ontologies: vo_with_imports.owl, so_with_imports.owl"}
{"asctime": "2021-11-01 07:40:55,252", "levelname": "INFO", "name": "builds.ontology_cleaning", "module": "ontology_cleaning", "funcName": "merge_ontologies", "lineno": 161, "message": "Merging Ontologies: ro_with_imports.owl, PheKnowLator_MergedOntologies.owl"}
{"asctime": "2021-11-01 07:41:01,303", "levelname": "INFO", "name": "builds.ontology_cleaning", "module": "ontology_cleaning", "funcName": "merge_ontologies", "lineno": 161, "message": "Merging Ontologies: pw_with_imports.owl, PheKnowLator_MergedOntologies.owl"}
{"asctime": "2021-11-01 07:41:09,946", "levelname": "INFO", "name": "builds.ontology_cleaning", "module": "ontology_cleaning", "funcName": "merge_ontologies", "lineno": 161, "message": "Merging Ontologies: pr_with_imports.owl, PheKnowLator_MergedOntologies.owl"}
{"asctime": "2021-11-01 07:41:55,436", "levelname": "INFO", "name": "builds.ontology_cleaning", "module": "ontology_cleaning", "funcName": "merge_ontologies", "lineno": 161, "message": "Merging Ontologies: mondo_with_imports.owl, PheKnowLator_MergedOntologies.owl"}
{"asctime": "2021-11-01 07:43:32,816", "levelname": "INFO", "name": "builds.ontology_cleaning", "module": "ontology_cleaning", "funcName": "merge_ontologies", "lineno": 161, "message": "Merging Ontologies: hp_with_imports.owl, PheKnowLator_MergedOntologies.owl"}
{"asctime": "2021-11-01 07:45:20,567", "levelname": "INFO", "name": "builds.ontology_cleaning", "module": "ontology_cleaning", "funcName": "merge_ontologies", "lineno": 161, "message": "Merging Ontologies: go_with_imports.owl, PheKnowLator_MergedOntologies.owl"}
{"asctime": "2021-11-01 07:47:28,284", "levelname": "INFO", "name": "builds.ontology_cleaning", "module": "ontology_cleaning", "funcName": "merge_ontologies", "lineno": 161, "message": "Merging Ontologies: ext_with_imports.owl, PheKnowLator_MergedOntologies.owl"}
{"asctime": "2021-11-01 07:49:47,810", "levelname": "INFO", "name": "builds.ontology_cleaning", "module": "ontology_cleaning", "funcName": "merge_ontologies", "lineno": 161, "message": "Merging Ontologies: clo_with_imports.owl, PheKnowLator_MergedOntologies.owl"}
{"asctime": "2021-11-01 07:52:32,133", "levelname": "INFO", "name": "builds.ontology_cleaning", "module": "ontology_cleaning", "funcName": "merge_ontologies", "lineno": 161, "message": "Merging Ontologies: chebi_with_imports.owl, PheKnowLator_MergedOntologies.owl"}
{"asctime": "2021-11-01 07:56:49,039", "levelname": "INFO", "name": "builds.ontology_cleaning", "module": "ontology_cleaning", "funcName": "cleans_ontology_data", "lineno": 604, "message": "Loading Merged Ontology"}
{"asctime": "2021-11-01 08:37:18,196", "levelname": "INFO", "name": "builds.ontology_cleaning", "module": "ontology_cleaning", "funcName": "updates_ontology_reporter", "lineno": 201, "message": "Obtaining Ontology Statistics"}
{"asctime": "2021-11-01 09:07:05,918", "levelname": "INFO", "name": "builds.ontology_cleaning", "module": "ontology_cleaning", "funcName": "fixes_identifier_errors", "lineno": 278, "message": "Fixing Identifier Errors"}
{"asctime": "2021-11-01 09:09:38,640", "levelname": "INFO", "name": "builds.ontology_cleaning", "module": "ontology_cleaning", "funcName": "normalizes_duplicate_classes", "lineno": 411, "message": "Normalizing Duplicate Concepts"}
{"asctime": "2021-11-01 09:09:38,641", "levelname": "INFO", "name": "builds.ontology_cleaning", "module": "ontology_cleaning", "funcName": "normalizes_existing_classes", "lineno": 444, "message": "Normalizing Existing Classes"}
{"asctime": "2021-11-01 09:10:10,035", "levelname": "INFO", "name": "builds.ontology_cleaning", "module": "ontology_cleaning", "funcName": "fixes_punning_errors", "lineno": 362, "message": "Resolving Punning Errors"}
{"asctime": "2021-11-01 09:13:36,293", "levelname": "INFO", "name": "builds.ontology_cleaning", "module": "ontology_cleaning", "funcName": "updates_ontology_reporter", "lineno": 201, "message": "Obtaining Ontology Statistics"}
{"asctime": "2021-11-01 09:57:17,472", "levelname": "INFO", "name": "builds.ontology_cleaning", "module": "ontology_cleaning", "funcName": "cleans_ontology_data", "lineno": 619, "message": "*** GENERATING ONTOLOGY CLEANING REPORT ***"}
{"asctime": "2021-11-01 09:57:17,931", "levelname": "INFO", "name": "builds.build_phase_2", "module": "build_phase_2", "funcName": "run_phase_2", "lineno": 165, "message": "Generating and Writing Preprocessed Data Metadata"}
{"asctime": "2021-11-01 10:00:03,648", "levelname": "INFO", "name": "builds.build_phase_2", "module": "build_phase_2", "funcName": "run_phase_2", "lineno": 181, "message": "Updating Input Dependency Documents"}
{"asctime": "2021-11-01 10:00:04,945", "levelname": "INFO", "name": "builds.build_phase_2", "module": "build_phase_2", "funcName": "run_phase_2", "lineno": 196, "message": "Uploading Processed Data to the temp_build_inprogress/data Directory"}
{"asctime": "2021-11-01 10:00:04,972", "levelname": "INFO", "name": "builds.build_phase_2", "module": "build_phase_2", "funcName": "run_phase_2", "lineno": 200, "message": "Copying Data FROM: archived_builds/release_v3.0.2/build_01NOV2021/data/ TO: temp_build_inprogress/data/"}
{"asctime": "2021-11-01 10:00:16,536", "levelname": "INFO", "name": "__main__", "module": "phases1_2_entrypoint", "funcName": "main", "lineno": 60, "message": " COMPLETED BUILD PHASES 1-2: 525.81 MINUTES "}
{"asctime": "2021-11-01 10:00:16,536", "levelname": "INFO", "name": "__main__", "module": "phases1_2_entrypoint", "funcName": "main", "lineno": 61, "message": "EXIT BUILD PHASES 1-2"}

Supplementary Document 7. pkt_build_log.log (Knowledge Graph Construction).

{"asctime": "2021-11-02 01:02:08,692", "levelname": "INFO", "name": "__main__", "module": "build_phase_3", "funcName": "main", "lineno": 72, "message": "#####\nBUILD PHASE 3: DATA PRE-PROCESSING\n#####"}
{"asctime": "2021-11-02 01:02:08,693", "levelname": "INFO", "name": "__main__", "module": "build_phase_3", "funcName": "main", "lineno": 77, "message": "STEP 1: INITIALIZE GCS BUCKET AND REFORMAT INPUT ARGUMENTS"}
{"asctime": "2021-11-02 01:02:08,985", "levelname": "INFO", "name": "__main__", "module": "build_phase_3", "funcName": "main", "lineno": 105, "message": "STEP 2: CONSTRUCT KNOWLEDGE GRAPH"}
{"asctime": "2021-11-02 01:02:08,985", "levelname": "INFO", "name": "__main__", "module": "build_phase_3", "funcName": "main", "lineno": 105, "message": "KG Build: subclass + relations_only.txt"}
{"asctime": "2021-11-02 01:02:12,818", "levelname": "INFO", "name": "pkt_kg.downloads", "module": "downloads", "funcName": "__init__", "lineno": 59, "message": "**********PKT STEP: DOWNLOADING KNOWLEDGE GRAPH DATA**********"}
{"asctime": "2021-11-02 01:02:12,821", "levelname": "INFO", "name": "pkt_kg.downloads", "module": "downloads", "funcName": "downloads_data_from_url", "lineno": 279, "message": "***Downloading Data: ontology_source_list to \"resources/ontologies/\" ***"}
{"asctime": "2021-11-02 01:02:12,830", "levelname": "INFO", "name": "pkt_kg.downloads", "module": "downloads", "funcName": "downloads_data_from_url", "lineno": 284, "message": "Downloading: hp_with_imports"}
{"asctime": "2021-11-02 01:02:28,895", "levelname": "INFO", "name": "pkt_kg.downloads", "module": "downloads", "funcName": "downloads_data_from_url", "lineno": 300, "message": "The knowledge graph contains 27169 classes, 341782 axioms, 256 object properties, and 0 individuals"}
{"asctime": "2021-11-02 01:02:28,896", "levelname": "INFO", "name": "pkt_kg.downloads", "module": "downloads", "funcName": "downloads_data_from_url", "lineno": 284, "message": "Downloading: go_with_imports"}
{"asctime": "2021-11-02 01:02:50,145", "levelname": "INFO", "name": "pkt_kg.downloads", "module": "downloads", "funcName": "downloads_data_from_url", "lineno": 300, "message": "The knowledge graph contains 43832 classes, 509854 axioms, 9 object properties, and 0 individuals"}
{"asctime": "2021-11-02 01:02:50,146", "levelname": "INFO", "name": "pkt_kg.downloads", "module": "downloads", "funcName": "downloads_data_from_url", "lineno": 284, "message": "Downloading: mondo_with_imports"}
{"asctime": "2021-11-02 01:03:26,234", "levelname": "INFO", "name": "pkt_kg.downloads", "module": "downloads", "funcName": "downloads_data_from_url", "lineno": 300, "message": "The knowledge graph contains 40975 classes, 612772 axioms, 338 object properties, and 17 individuals"}
{"asctime": "2021-11-02 01:03:26,235", "levelname": "INFO", "name": "pkt_kg.downloads", "module": "downloads", "funcName": "downloads_data_from_url", "lineno": 284, "message": "Downloading: vo_with_imports"}
{"asctime": "2021-11-02 01:03:30,130", "levelname": "INFO", "name": "pkt_kg.downloads", "module": "downloads", "funcName": "downloads_data_from_url", "lineno": 300, "message": "The knowledge graph contains 6825 classes, 62120 axioms, 232 object properties, and 167 individuals"}
{"asctime": "2021-11-02 01:03:30,131", "levelname": "INFO", "name": "pkt_kg.downloads", "module": "downloads", "funcName": "downloads_data_from_url", "lineno": 284, "message": "Downloading: chebi_with_imports"}
{"asctime": "2021-11-02 01:04:33,057", "levelname": "INFO", "name": "pkt_kg.downloads", "module": "downloads", "funcName": "downloads_data_from_url", "lineno": 300, "message": "The knowledge graph contains 150080 classes, 2719571 axioms, 10 object properties, and 0 individuals"}
{"asctime": "2021-11-02 01:04:33,058", "levelname": "INFO", "name": "pkt_kg.downloads", "module": "downloads", "funcName": "downloads_data_from_url", "lineno": 284, "message": "Downloading: ext_with_imports"}
{"asctime": "2021-11-02 01:04:47,341", "levelname": "INFO", "name": "pkt_kg.downloads", "module": "downloads", "funcName": "downloads_data_from_url", "lineno": 300, "message": "The knowledge graph contains 19096 classes, 266646 axioms, 239 object properties, and 0 individuals"}
{"asctime": "2021-11-02 01:04:47,342", "levelname": "INFO", "name": "pkt_kg.downloads", "module": "downloads", "funcName": "downloads_data_from_url", "lineno": 284, "message": "Downloading: clo_with_imports"}
{"asctime": "2021-11-02 01:05:07,293", "levelname": "INFO", "name": "pkt_kg.downloads", "module": "downloads", "funcName": "downloads_data_from_url", "lineno": 300, "message": "The knowledge graph contains 44858 classes, 548206 axioms, 112 object properties, and 33 individuals"}
{"asctime": "2021-11-02 01:05:07,294", "levelname": "INFO", "name": "pkt_kg.downloads", "module": "downloads", "funcName": "downloads_data_from_url", "lineno": 284, "message": "Downloading: pr_with_imports"}
{"asctime": "2021-11-02 01:05:34,557", "levelname": "INFO", "name": "pkt_kg.downloads", "module": "downloads", "funcName": "downloads_data_from_url", "lineno": 300, "message": "The knowledge graph contains 117081 classes, 1385427 axioms, 12 object properties, and 0 individuals"}
{"asctime": "2021-11-02 01:05:34,558", "levelname": "INFO", "name": "pkt_kg.downloads", "module": "downloads", "funcName": "downloads_data_from_url", "lineno": 284, "message": "Downloading: so_with_imports"}
{"asctime": "2021-11-02 01:05:37,171", "levelname": "INFO", "name": "pkt_kg.downloads", "module": "downloads", "funcName": "downloads_data_from_url", "lineno": 300, "message": "The knowledge graph contains 2363 classes, 23204 axioms, 50 object properties, and 0 individuals"}
{"asctime": "2021-11-02 01:05:37,171", "levelname": "INFO", "name": "pkt_kg.downloads", "module": "downloads", "funcName": "downloads_data_from_url", "lineno": 284, "message": "Downloading: pw_with_imports"}
{"asctime": "2021-11-02 01:05:39,867", "levelname": "INFO", "name": "pkt_kg.downloads", "module": "downloads", "funcName": "downloads_data_from_url", "lineno": 300, "message": "The knowledge graph contains 2600 classes, 21868 axioms, 1 object properties, and 0 individuals"}
{"asctime": "2021-11-02 01:05:39,868", "levelname": "INFO", "name": "pkt_kg.downloads", "module": "downloads", "funcName": "downloads_data_from_url", "lineno": 284, "message": "Downloading: ro_with_imports"}
{"asctime": "2021-11-02 01:05:41,962", "levelname": "INFO", "name": "pkt_kg.downloads", "module": "downloads", "funcName": "downloads_data_from_url", "lineno": 300, "message": "The knowledge graph contains 69 classes, 5823 axioms, 600 object properties, and 5 individuals"}
{"asctime": "2021-11-02 01:05:41,964", "levelname": "INFO", "name": "pkt_kg.downloads", "module": "downloads", "funcName": "generates_source_metadata", "lineno": 198, "message": "*** Generating Metadata ***"}
{"asctime": "2021-11-02 01:05:42,124", "levelname": "INFO", "name": "pkt_kg.downloads", "module": "downloads", "funcName": "__init__", "lineno": 59, "message": "**********PKT STEP: DOWNLOADING KNOWLEDGE GRAPH DATA**********"}
{"asctime": "2021-11-02 01:05:42,128", "levelname": "INFO", "name": "pkt_kg.downloads", "module": "downloads", "funcName": "downloads_data_from_url", "lineno": 348, "message": "*** Downloading Data: edge_source_list to \"resources/edge_data/\" ***"}
{"asctime": "2021-11-02 01:05:42,129", "levelname": "INFO", "name": "pkt_kg.downloads", "module": "downloads", "funcName": "downloads_data_from_url", "lineno": 353, "message": "Edge: chemical-disease"}
{"asctime": "2021-11-02 01:05:46,847", "levelname": "INFO", "name": "pkt_kg.downloads", "module": "downloads", "funcName": "downloads_data_from_url", "lineno": 353, "message": "Edge: chemical-gene"}
{"asctime": "2021-11-02 01:05:50,002", "levelname": "INFO", "name": "pkt_kg.downloads", "module": "downloads", "funcName": "downloads_data_from_url", "lineno": 353, "message": "Edge: chemical-gobp"}
{"asctime": "2021-11-02 01:05:55,511", "levelname": "INFO", "name": "pkt_kg.downloads", "module": "downloads", "funcName": "downloads_data_from_url", "lineno": 353, "message": "Edge: chemical-gocc"}
{"asctime": "2021-11-02 01:05:56,544", "levelname": "INFO", "name": "pkt_kg.downloads", "module": "downloads", "funcName": "downloads_data_from_url", "lineno": 353, "message": "Edge: chemical-gomf"}
{"asctime": "2021-11-02 01:05:57,597", "levelname": "INFO", "name": "pkt_kg.downloads", "module": "downloads", "funcName": "downloads_data_from_url", "lineno": 353, "message": "Edge: chemical-pathway"}
{"asctime": "2021-11-02 01:05:57,852", "levelname": "INFO", "name": "pkt_kg.downloads", "module": "downloads", "funcName": "downloads_data_from_url", "lineno": 353, "message": "Edge: chemical-phenotype"}
{"asctime": "2021-11-02 01:05:58,730", "levelname": "INFO", "name": "pkt_kg.downloads", "module": "downloads", "funcName": "downloads_data_from_url", "lineno": 353, "message": "Edge: chemical-protein"}
{"asctime": "2021-11-02 01:05:59,290", "levelname": "INFO", "name": "pkt_kg.downloads", "module": "downloads", "funcName": "downloads_data_from_url", "lineno": 353, "message": "Edge: disease-phenotype"}
{"asctime": "2021-11-02 01:05:59,665", "levelname": "INFO", "name": "pkt_kg.downloads", "module": "downloads", "funcName": "downloads_data_from_url", "lineno": 353, "message": "Edge: gene-disease"}
{"asctime": "2021-11-02 01:05:59,810", "levelname": "INFO", "name": "pkt_kg.downloads", "module": "downloads", "funcName": "downloads_data_from_url", "lineno": 353, "message": "Edge: gene-gene"}
{"asctime": "2021-11-02 01:06:01,624", "levelname": "INFO", "name": "pkt_kg.downloads", "module": "downloads", "funcName": "downloads_data_from_url", "lineno": 353, "message": "Edge: gene-pathway"}
{"asctime": "2021-11-02 01:06:01,694", "levelname": "INFO", "name": "pkt_kg.downloads", "module": "downloads", "funcName": "downloads_data_from_url", "lineno": 353, "message": "Edge: gene-phenotype"}
{"asctime": "2021-11-02 01:06:01,709", "levelname": "INFO", "name": "pkt_kg.downloads", "module": "downloads", "funcName": "downloads_data_from_url", "lineno": 353, "message": "Edge: gene-protein"}
{"asctime": "2021-11-02 01:06:01,735", "levelname": "INFO", "name": "pkt_kg.downloads", "module": "downloads", "funcName": "downloads_data_from_url", "lineno": 353, "message": "Edge: gene-rna"}
{"asctime": "2021-11-02 01:06:01,910", "levelname": "INFO", "name": "pkt_kg.downloads", "module": "downloads", "funcName": "downloads_data_from_url", "lineno": 353, "message": "Edge: gobp-pathway"}
{"asctime": "2021-11-02 01:06:02,114", "levelname": "INFO", "name": "pkt_kg.downloads", "module": "downloads", "funcName": "downloads_data_from_url", "lineno": 353, "message": "Edge: pathway-gocc"}
{"asctime": "2021-11-02 01:06:02,130", "levelname": "INFO", "name": "pkt_kg.downloads", "module": "downloads", "funcName": "downloads_data_from_url", "lineno": 353, "message": "Edge: pathway-gomf"}
{"asctime": "2021-11-02 01:06:02,146", "levelname": "INFO", "name": "pkt_kg.downloads", "module": "downloads", "funcName": "downloads_data_from_url", "lineno": 353, "message": "Edge: protein-anatomy"}
{"asctime": "2021-11-02 01:06:02,313", "levelname": "INFO", "name": "pkt_kg.downloads", "module": "downloads", "funcName": "downloads_data_from_url", "lineno": 353, "message": "Edge: protein-catalyst"}
{"asctime": "2021-11-02 01:06:02,342", "levelname": "INFO", "name": "pkt_kg.downloads", "module": "downloads", "funcName": "downloads_data_from_url", "lineno": 353, "message": "Edge: protein-cell"}
{"asctime": "2021-11-02 01:06:02,369", "levelname": "INFO", "name": "pkt_kg.downloads", "module": "downloads", "funcName": "downloads_data_from_url", "lineno": 353, "message": "Edge: protein-cofactor"}
{"asctime": "2021-11-02 01:06:02,384", "levelname": "INFO", "name": "pkt_kg.downloads", "module": "downloads", "funcName": "downloads_data_from_url", "lineno": 353, "message": "Edge: protein-gobp"}
{"asctime": "2021-11-02 01:06:03,132", "levelname": "INFO", "name": "pkt_kg.downloads", "module": "downloads", "funcName": "downloads_data_from_url", "lineno": 353, "message": "Edge: protein-gocc"}
{"asctime": "2021-11-02 01:06:03,274", "levelname": "INFO", "name": "pkt_kg.downloads", "module": "downloads", "funcName": "downloads_data_from_url", "lineno": 353, "message": "Edge: protein-gomf"}
{"asctime": "2021-11-02 01:06:03,410", "levelname": "INFO", "name": "pkt_kg.downloads", "module": "downloads", "funcName": "downloads_data_from_url", "lineno": 353, "message": "Edge: protein-pathway"}
{"asctime": "2021-11-02 01:06:04,093", "levelname": "INFO", "name": "pkt_kg.downloads", "module": "downloads", "funcName": "downloads_data_from_url", "lineno": 353, "message": "Edge: protein-protein"}
{"asctime": "2021-11-02 01:06:07,765", "levelname": "INFO", "name": "pkt_kg.downloads", "module": "downloads", "funcName": "downloads_data_from_url", "lineno": 353, "message": "Edge: rna-anatomy"}
{"asctime": "2021-11-02 01:06:07,793", "levelname": "INFO", "name": "pkt_kg.downloads", "module": "downloads", "funcName": "downloads_data_from_url", "lineno": 353, "message": "Edge: rna-cell"}

{"asctime": "2021-11-02 01:06:07,820", "levelname": "INFO", "name": "pkt_kg.downloads", "module": "downloads", "funcName": "downloads_data_from_url", "lineno": 353, "message": "Edge: rna-protein"}
{"asctime": "2021-11-02 01:06:07,872", "levelname": "INFO", "name": "pkt_kg.downloads", "module": "downloads", "funcName": "downloads_data_from_url", "lineno": 353, "message": "Edge: variant-disease"}
{"asctime": "2021-11-02 01:06:32,380", "levelname": "INFO", "name": "pkt_kg.downloads", "module": "downloads", "funcName": "downloads_data_from_url", "lineno": 353, "message": "Edge: variant-gene"}
{"asctime": "2021-11-02 01:06:37,059", "levelname": "INFO", "name": "pkt_kg.downloads", "module": "downloads", "funcName": "downloads_data_from_url", "lineno": 353, "message": "Edge: variant-phenotype"}
{"asctime": "2021-11-02 01:06:42,265", "levelname": "INFO", "name": "pkt_kg.downloads", "module": "downloads", "funcName": "generates_source_metadata", "lineno": 198, "message": "*** Generating Metadata ***"}
{"asctime": "2021-11-02 01:06:46,061", "levelname": "INFO", "name": "pkt_kg.edge_list", "module": "edge_list", "funcName": "runs_creates_knowledge_graph_edges", "lineno": 401, "message": "**********PKT STEP: GENERATING KNOWLEDGE GRAPH MASTER EDGE LIST**********"}
{"asctime": "2021-11-02 01:08:34,019", "levelname": "INFO", "name": "pkt_kg.edge_list", "module": "edge_list", "funcName": "creates_knowledge_graph_edges", "lineno": 383, "message": "Finished Edge: chemical-gene (chemical = 466, gene = 11978); 16708 unique edges"}
{"asctime": "2021-11-02 01:08:41,757", "levelname": "INFO", "name": "pkt_kg.edge_list", "module": "edge_list", "funcName": "creates_knowledge_graph_edges", "lineno": 383, "message": "Finished Edge: chemical-pathway (chemical = 2247, pathway = 2243); 29988 unique edges"}
{"asctime": "2021-11-02 01:08:46,193", "levelname": "INFO", "name": "pkt_kg.edge_list", "module": "edge_list", "funcName": "creates_knowledge_graph_edges", "lineno": 383, "message": "Finished Edge: gene-disease (gene = 5060, disease = 4436); 12842 unique edges"}
{"asctime": "2021-11-02 01:08:46,943", "levelname": "INFO", "name": "pkt_kg.edge_list", "module": "edge_list", "funcName": "creates_knowledge_graph_edges", "lineno": 383, "message": "Finished Edge: gene-protein (gene = 19316, protein = 19134); 19521 unique edges"}
{"asctime": "2021-11-02 01:08:50,589", "levelname": "INFO", "name": "pkt_kg.edge_list", "module": "edge_list", "funcName": "creates_knowledge_graph_edges", "lineno": 383, "message": "Finished Edge: pathway-gomf (pathway = 2422, gomf = 728); 2426 unique edges"}
{"asctime": "2021-11-02 01:08:50,680", "levelname": "INFO", "name": "pkt_kg.edge_list", "module": "edge_list", "funcName": "creates_knowledge_graph_edges", "lineno": 383, "message": "Finished Edge: protein-cofactor (protein = 1584, cofactor = 44); 1998 unique edges"}
{"asctime": "2021-11-02 01:09:16,316", "levelname": "INFO", "name": "pkt_kg.edge_list", "module": "edge_list", "funcName": "creates_knowledge_graph_edges", "lineno": 383, "message": "Finished Edge: protein-pathway (protein = 10546, pathway = 2507); 117813 unique edges"}
{"asctime": "2021-11-02 01:09:18,343", "levelname": "INFO", "name": "pkt_kg.edge_list", "module": "edge_list", "funcName": "creates_knowledge_graph_edges", "lineno": 383, "message": "Finished Edge: rna-protein (rna = 44202, protein = 19200); 44205 unique edges"}
{"asctime": "2021-11-02 01:09:57,823", "levelname": "INFO", "name": "pkt_kg.edge_list", "module": "edge_list", "funcName": "creates_knowledge_graph_edges", "lineno": 383, "message": "Finished Edge: chemical-gocc (chemical = 1121, gocc = 262); 47716 unique edges"}
{"asctime": "2021-11-02 01:10:17,756", "levelname": "INFO", "name": "pkt_kg.edge_list", "module": "edge_list", "funcName": "creates_knowledge_graph_edges", "lineno": 383, "message": "Finished Edge: chemical-disease (chemical = 4341, disease = 4583); 172573 unique edges"}
{"asctime": "2021-11-02 01:11:24,875", "levelname": "INFO", "name": "pkt_kg.edge_list", "module": "edge_list", "funcName": "creates_knowledge_graph_edges", "lineno": 383, "message": "Finished Edge: chemical-protein (chemical = 4272, protein = 7946); 71679 unique edges"}
{"asctime": "2021-11-02 01:11:29,606", "levelname": "INFO", "name": "pkt_kg.edge_list", "module": "edge_list", "funcName": "creates_knowledge_graph_edges", "lineno": 383, "message": "Finished Edge: gene-pathway (gene = 10369, pathway = 1860); 107009 unique edges"}
{"asctime": "2021-11-02 01:11:32,172", "levelname": "INFO", "name": "pkt_kg.edge_list", "module": "edge_list", "funcName": "creates_knowledge_graph_edges", "lineno": 383, "message": "Finished Edge: gobp-pathway (gobp = 479, pathway = 672); 672 unique edges"}
{"asctime": "2021-11-02 01:11:32,499", "levelname": "INFO", "name": "pkt_kg.edge_list", "module": "edge_list", "funcName": "creates_knowledge_graph_edges", "lineno": 383, "message": "Finished Edge: protein-catalyst (protein = 3049, catalyst = 3758); 25136 unique edges"}
{"asctime": "2021-11-02 01:11:34,088", "levelname": "INFO", "name": "pkt_kg.edge_list", "module": "edge_list", "funcName": "creates_knowledge_graph_edges", "lineno": 383, "message": "Finished Edge: chemical-gobp (chemical = 1350, gobp = 1510); 288873 unique edges"}
{"asctime": "2021-11-02 01:11:53,756", "levelname": "INFO", "name": "pkt_kg.edge_list", "module": "edge_list", "funcName": "creates_knowledge_graph_edges", "lineno": 383, "message": "Finished Edge: protein-gocc (protein = 18451, gocc = 1752); 82526 unique edges"}
{"asctime": "2021-11-02 01:12:07,668", "levelname": "INFO", "name": "pkt_kg.edge_list", "module": "edge_list", "funcName": "creates_knowledge_graph_edges", "lineno": 383, "message": "Finished Edge: rna-anatomy (rna = 29121, anatomy = 103); 444974 unique edges"}
{"asctime": "2021-11-02 01:12:47,883", "levelname": "INFO", "name": "pkt_kg.edge_list", "module": "edge_list", "funcName": "creates_knowledge_graph_edges", "lineno": 383, "message": "Finished Edge: chemical-gomf (chemical = 1133, gomf = 214); 28077 unique edges"}
{"asctime": "2021-11-02 01:12:57,504", "levelname": "INFO", "name": "pkt_kg.edge_list", "module": "edge_list", "funcName": "creates_knowledge_graph_edges", "lineno": 383, "message": "Finished Edge: disease-phenotype (disease = 11930, phenotype = 10068); 435102 unique edges"}
{"asctime": "2021-11-02 01:13:01,176", "levelname": "INFO", "name": "pkt_kg.edge_list", "module": "edge_list", "funcName": "creates_knowledge_graph_edges", "lineno": 383, "message": "Finished Edge: gene-phenotype (gene = 6785, phenotype = 1597); 24760 unique edges"}
{"asctime": "2021-11-02 01:13:06,564", "levelname": "INFO", "name": "pkt_kg.edge_list", "module": "edge_list", "funcName": "creates_knowledge_graph_edges", "lineno": 383, "message": "Finished Edge: pathway-gocc (pathway = 11252, gocc = 99); 16014 unique edges"}
{"asctime": "2021-11-02 01:13:14,213", "levelname": "INFO", "name": "pkt_kg.edge_list", "module": "edge_list", "funcName": "creates_knowledge_graph_edges", "lineno": 383, "message": "Finished Edge: protein-cell (protein = 10044, cell = 128); 75313 unique edges"}
{"asctime": "2021-11-02 01:13:37,715", "levelname": "INFO", "name": "pkt_kg.edge_list", "module": "edge_list", "funcName": "creates_knowledge_graph_edges", "lineno": 383, "message": "Finished Edge: protein-gomf (protein = 17801, gomf = 4430); 69801 unique edges"}
{"asctime": "2021-11-02 01:13:47,777", "levelname": "INFO", "name": "pkt_kg.edge_list", "module": "edge_list", "funcName": "creates_knowledge_graph_edges", "lineno": 383, "message": "Finished Edge: rna-cell (rna = 14044, cell = 130); 65180 unique edges"}
{"asctime": "2021-11-02 01:14:26,632", "levelname": "INFO", "name": "pkt_kg.edge_list", "module": "edge_list", "funcName": "creates_knowledge_graph_edges", "lineno": 383, "message": "Finished Edge: chemical-phenotype (chemical = 4102, phenotype = 1742); 110898 unique edges"}
{"asctime": "2021-11-02 01:15:15,286", "levelname": "INFO", "name": "pkt_kg.edge_list", "module": "edge_list", "funcName": "creates_knowledge_graph_edges", "lineno": 383, "message": "Finished Edge: gene-gene (gene = 250, gene = 267); 1694 unique edges"}
{"asctime": "2021-11-02 01:15:18,594", "levelname": "INFO", "name": "pkt_kg.edge_list", "module": "edge_list", "funcName": "creates_knowledge_graph_edges", "lineno": 383, "message": "Finished Edge: gene-rna (gene = 25527, rna = 179872); 182692 unique edges"}
{"asctime": "2021-11-02 01:15:26,108", "levelname": "INFO", "name": "pkt_kg.edge_list", "module": "edge_list", "funcName": "creates_knowledge_graph_edges", "lineno": 383, "message": "Finished Edge: protein-anatomy (protein = 10746, anatomy = 68); 30681 unique edges"}
{"asctime": "2021-11-02 01:15:48,464", "levelname": "INFO", "name": "pkt_kg.edge_list", "module": "edge_list", "funcName": "creates_knowledge_graph_edges", "lineno": 383, "message": "Finished Edge: protein-gobp (protein = 17404, gobp = 12329); 129424 unique edges"}
{"asctime": "2021-11-02 01:20:08,039", "levelname": "INFO", "name": "pkt_kg.edge_list", "module": "edge_list", "funcName": "creates_knowledge_graph_edges", "lineno": 383, "message": "Finished Edge: protein-protein (protein = 14230, protein = 14230); 618069 unique edges"}
{"asctime": "2021-11-02 01:21:54,271", "levelname": "INFO", "name": "pkt_kg.edge_list", "module": "edge_list", "funcName": "creates_knowledge_graph_edges", "lineno": 383, "message": "Finished Edge: variant-gene (variant = 145129, gene = 3626); 145129 unique edges"}
{"asctime": "2021-11-02 01:21:59,856", "levelname": "INFO", "name": "pkt_kg.edge_list", "module": "edge_list", "funcName": "creates_knowledge_graph_edges", "lineno": 383, "message": "Finished Edge: variant-phenotype (variant = 2100, phenotype = 436); 3081 unique edges"}
{"asctime": "2021-11-02 01:26:14,303", "levelname": "INFO", "name": "pkt_kg.edge_list", "module": "edge_list", "funcName": "creates_knowledge_graph_edges", "lineno": 383, "message": "Finished Edge: variant-disease (variant = 14732, disease = 3753); 43439 unique edges"}
{"asctime": "2021-11-02 01:27:05,318", "levelname": "INFO", "name": "pkt_kg.knowledge_graph", "module": "knowledge_graph", "funcName": "construct_knowledge_graph", "lineno": 543, "message": "**********PKT STEP: CONSTRUCTING KNOWLEDGE GRAPH**********\n### Starting Knowledge Graph Build: FULL ###"}
{"asctime": "2021-11-02 01:27:05,318", "levelname": "INFO", "name": "pkt_kg.knowledge_graph", "module": "knowledge_graph", "funcName": "construct_knowledge_graph", "lineno": 546, "message": "*** Loading Relations Data ***"}
{"asctime": "2021-11-02 01:27:05,319", "levelname": "INFO", "name": "pkt_kg.knowledge_graph", "module": "knowledge_graph", "funcName": "construct_knowledge_graph", "lineno": 551, "message": "*** Loading Merged Ontologies ***"}
{"asctime": "2021-11-02 02:24:06,890", "levelname": "INFO", "name": "pkt_kg.knowledge_graph", "module": "knowledge_graph", "funcName": "construct_knowledge_graph", "lineno": 557, "message": "Merged Ontologies Graph Stats: 13933782 triples, 5716554 nodes, 355 predicates, 558715 classes, 190 individuals, 853 object props, 635 annotation props"}
{"asctime": "2021-11-02 02:24:06,890", "levelname": "INFO", "name": "pkt_kg.knowledge_graph", "module": "knowledge_graph", "funcName": "construct_knowledge_graph", "lineno": 560, "message": "*** Loading Node Metadata Data ***"}
{"asctime": "2021-11-02 02:24:06,891", "levelname": "INFO", "name": "pkt_kg.metadata", "module": "metadata", "funcName": "metadata_processor", "lineno": 80, "message": "Loading and Processing Node Metadata"}
{"asctime": "2021-11-02 02:24:09,201", "levelname": "INFO", "name": "pkt_kg.metadata", "module": "metadata", "funcName": "extract_metadata", "lineno": 116, "message": "Extracting Class and Relation Metadata"}
{"asctime": "2021-11-02 02:26:39,115", "levelname": "INFO", "name": "pkt_kg.knowledge_graph", "module": "knowledge_graph", "funcName": "construct_knowledge_graph", "lineno": 565, "message": "*** Splitting Graph ***"}
{"asctime": "2021-11-02 03:20:03,997", "levelname": "INFO", "name": "pkt_kg.knowledge_graph", "module": "knowledge_graph", "funcName": "construct_knowledge_graph", "lineno": 567, "message": "Merged Ontologies - Logic Subset Graph Stats: 4103821 triples, 1420834 nodes, 43 predicates, 558715 classes, 190 individuals, 853 object props, 635 annotation props"}
{"asctime": "2021-11-02 03:23:44,592", "levelname": "INFO", "name": "pkt_kg.knowledge_graph", "module": "knowledge_graph", "funcName": "construct_knowledge_graph", "lineno": 574, "message": "*** Building Knowledge Graph Edges ***"}
{"asctime": "2021-11-02 03:58:16,555", "levelname": "INFO", "name": "pkt_kg.knowledge_graph", "module": "knowledge_graph", "funcName": "creates_new_edges", "lineno": 347, "message": "Created CHEMICAL-GOBP (class-class) Edges: 1722549 OWL Edges, 286618 Original Edges; 576080 OWL Nodes, Original Nodes: 1350 chemical(s), 1490 gobp(s)"}
{"asctime": "2021-11-02 04:15:32,538", "levelname": "INFO", "name": "pkt_kg.knowledge_graph", "module": "knowledge_graph", "funcName": "creates_new_edges", "lineno": 347, "message": "Created DISEASE-PHENOTYPE (class-class) Edges: 2592087 OWL Edges, 428374 Original Edges; 878594 OWL Nodes, Original Nodes: 11780 disease(s), 10062 phenotype(s)"}
{"asctime": "2021-11-02 04:29:10,127", "levelname": "INFO", "name": "pkt_kg.knowledge_graph", "module": "knowledge_graph", "funcName": "creates_new_edges", "lineno": 347, "message": "Created GENE-RNA (entity-entity) Edges: 1686851 OWL Edges, 182692 Original Edges; 570815 OWL Nodes, Original Nodes: 25527 gene(s), 179872 rna(s)"}
{"asctime": "2021-11-02 04:34:08,778", "levelname": "INFO", "name": "pkt_kg.knowledge_graph", "module": "knowledge_graph", "funcName": "creates_new_edges", "lineno": 347, "message": "Created RNA-ANATOMY (entity-class) Edges: 2728768 OWL Edges, 440217 Original Edges; 909683 OWL Nodes, Original Nodes: 29110 rna(s), 102 anatomy(s)"}
{"asctime": "2021-11-02 04:34:47,245", "levelname": "INFO", "name": "pkt_kg.knowledge_graph", "module": "knowledge_graph", "funcName": "creates_new_edges", "lineno": 347, "message": "Created CHEMICAL-DISEASE (class-class) Edges: 1021872 OWL Edges, 168841 Original Edges; 346511 OWL Nodes, Original Nodes: 4339 chemical(s), 4486 disease(s)"}

{"asctime": "2021-11-02 04:40:18,626", "levelname": "INFO", "name": "pkt_kg.knowledge_graph", "module": "knowledge_graph", "funcName": "creates_new_edges", "lineno": 347, "message": "Created PROTEIN-PROTEIN (class-class) Edges: 3722645 OWL Edges, 618069 Original Edges; 1250372 OWL Nodes, Original Nodes: 14230 protein(s), 14230 protein(s)"}
{"asctime": "2021-11-02 04:44:04,526", "levelname": "INFO", "name": "pkt_kg.knowledge_graph", "module": "knowledge_graph", "funcName": "creates_new_edges", "lineno": 347, "message": "Created PROTEIN-GOBP (class-class) Edges: 806278 OWL Edges, 129424 Original Edges; 288585 OWL Nodes, Original Nodes: 17404 protein(s), 12329 gobp(s)"}
{"asctime": "2021-11-02 04:49:59,442", "levelname": "INFO", "name": "pkt_kg.knowledge_graph", "module": "knowledge_graph", "funcName": "creates_new_edges", "lineno": 347, "message": "Created PROTEIN-GOCC (class-class) Edges: 515360 OWL Edges, 82526 Original Edges; 185259 OWL Nodes, Original Nodes: 18451 protein(s), 1752 gocc(s)"}
{"asctime": "2021-11-02 04:55:23,205", "levelname": "INFO", "name": "pkt_kg.knowledge_graph", "module": "knowledge_graph", "funcName": "creates_new_edges", "lineno": 347, "message": "Created GENE-PATHWAY (entity-entity) Edges: 655865 OWL Edges, 104891 Original Edges; 222723 OWL Nodes, Original Nodes: 10369 gene(s), 1809 pathway(s)"}
{"asctime": "2021-11-02 04:56:44,758", "levelname": "INFO", "name": "pkt_kg.knowledge_graph", "module": "knowledge_graph", "funcName": "creates_new_edges", "lineno": 347, "message": "Created VARIANT-GENE (entity-entity) Edges: 1168894 OWL Edges, 145129 Original Edges; 439031 OWL Nodes, Original Nodes: 145129 variant(s), 3626 gene(s)"}
{"asctime": "2021-11-02 04:56:51,244", "levelname": "INFO", "name": "pkt_kg.knowledge_graph", "module": "knowledge_graph", "funcName": "creates_new_edges", "lineno": 347, "message": "Created CHEMICAL-PHENOTYPE (class-class) Edges: 667099 OWL Edges, 110211 Original Edges; 226258 OWL Nodes, Original Nodes: 4100 chemical(s), 1732 phenotype(s)"}
{"asctime": "2021-11-02 04:58:30,807", "levelname": "INFO", "name": "pkt_kg.knowledge_graph", "module": "knowledge_graph", "funcName": "creates_new_edges", "lineno": 347, "message": "Created PROTEIN-CELL (class-class) Edges: 451320 OWL Edges, 73525 Original Edges; 157223 OWL Nodes, Original Nodes: 10044 protein(s), 125 cell(s)"}
{"asctime": "2021-11-02 05:03:40,898", "levelname": "INFO", "name": "pkt_kg.knowledge_graph", "module": "knowledge_graph", "funcName": "creates_new_edges", "lineno": 347, "message": "Created CHEMICAL-GOCC (class-class) Edges: 264351 OWL Edges, 43832 Original Edges; 89026 OWL Nodes, Original Nodes: 1121 chemical(s), 237 gocc(s)"}
{"asctime": "2021-11-02 05:04:55,020", "levelname": "INFO", "name": "pkt_kg.knowledge_graph", "module": "knowledge_graph", "funcName": "creates_new_edges", "lineno": 347, "message": "Created PROTEIN-GOMF (class-class) Edges: 441038 OWL Edges, 69801 Original Edges; 161837 OWL Nodes, Original Nodes: 17801 protein(s), 4430 gomf(s)"}
{"asctime": "2021-11-02 05:05:12,368", "levelname": "INFO", "name": "pkt_kg.knowledge_graph", "module": "knowledge_graph", "funcName": "creates_new_edges", "lineno": 347, "message": "Created RNA-CELL (entity-class) Edges: 428998 OWL Edges, 64451 Original Edges; 143109 OWL Nodes, Original Nodes: 14044 rna(s), 127 cell(s)"}
{"asctime": "2021-11-02 05:06:21,474", "levelname": "INFO", "name": "pkt_kg.knowledge_graph", "module": "knowledge_graph", "funcName": "creates_new_edges", "lineno": 347, "message": "Created PROTEIN-CATALYST (class-class) Edges: 149562 OWL Edges, 23794 Original Edges; 54389 OWL Nodes, Original Nodes: 3048 protein(s), 3749 catalyst(s)"}
{"asctime": "2021-11-02 05:08:22,977", "levelname": "INFO", "name": "pkt_kg.knowledge_graph", "module": "knowledge_graph", "funcName": "creates_new_edges", "lineno": 347, "message": "Created PROTEIN-ANATOMY (class-class) Edges: 194901 OWL Edges, 30681 Original Edges; 72180 OWL Nodes, Original Nodes: 10746 protein(s), 68 anatomy(s)"}
{"asctime": "2021-11-02 05:08:45,554", "levelname": "INFO", "name": "pkt_kg.knowledge_graph", "module": "knowledge_graph", "funcName": "creates_new_edges", "lineno": 347, "message": "Created PATHWAY-GOCC (entity-class) Edges: 119256 OWL Edges, 16014 Original Edges; 43559 OWL Nodes, Original Nodes: 11252 pathway(s), 99 gocc(s)"}
{"asctime": "2021-11-02 05:09:15,503", "levelname": "INFO", "name": "pkt_kg.knowledge_graph", "module": "knowledge_graph", "funcName": "creates_new_edges", "lineno": 347, "message": "Created PROTEIN-COFACTOR (class-class) Edges: 13388 OWL Edges, 1960 Original Edges; 5551 OWL Nodes, Original Nodes: 1583 protein(s), 44 cofactor(s)"}
{"asctime": "2021-11-02 05:10:51,535", "levelname": "INFO", "name": "pkt_kg.knowledge_graph", "module": "knowledge_graph", "funcName": "creates_new_edges", "lineno": 347, "message": "Created VARIANT-DISEASE (entity-class) Edges: 291895 OWL Edges, 43088 Original Edges; 104581 OWL Nodes, Original Nodes: 14712 variant(s), 3683 disease(s)"}
{"asctime": "2021-11-02 05:13:24,844", "levelname": "INFO", "name": "pkt_kg.knowledge_graph", "module": "knowledge_graph", "funcName": "creates_new_edges", "lineno": 347, "message": "Created CHEMICAL-GOMF (class-class) Edges: 164592 OWL Edges, 27209 Original Edges; 55759 OWL Nodes, Original Nodes: 1133 chemical(s), 204 gomf(s)"}
{"asctime": "2021-11-02 05:14:08,016", "levelname": "INFO", "name": "pkt_kg.knowledge_graph", "module": "knowledge_graph", "funcName": "creates_new_edges", "lineno": 347, "message": "Created CHEMICAL-PATHWAY (class-entity) Edges: 188972 OWL Edges, 29973 Original Edges; 65285 OWL Nodes, Original Nodes: 2245 chemical(s), 2243 pathway(s)"}
{"asctime": "2021-11-02 05:14:57,582", "levelname": "INFO", "name": "pkt_kg.knowledge_graph", "module": "knowledge_graph", "funcName": "creates_new_edges", "lineno": 347, "message": "Created GENE-DISEASE (entity-class) Edges: 91269 OWL Edges, 12808 Original Edges; 35057 OWL Nodes, Original Nodes: 5056 gene(s), 4422 disease(s)"}
{"asctime": "2021-11-02 05:15:14,241", "levelname": "INFO", "name": "pkt_kg.knowledge_graph", "module": "knowledge_graph", "funcName": "creates_new_edges", "lineno": 347, "message": "Created PATHWAY-GOMF (entity-class) Edges: 20272 OWL Edges, 2426 Original Edges; 8065 OWL Nodes, Original Nodes: 2422 pathway(s), 728 gomf(s)"}
{"asctime": "2021-11-02 05:15:45,160", "levelname": "INFO", "name": "pkt_kg.knowledge_graph", "module": "knowledge_graph", "funcName": "creates_new_edges", "lineno": 347, "message": "Created PROTEIN-PATHWAY (class-entity) Edges: 722480 OWL Edges, 117410 Original Edges; 248711 OWL Nodes, Original Nodes: 10512 protein(s), 2495 pathway(s)"}
{"asctime": "2021-11-02 05:16:10,820", "levelname": "INFO", "name": "pkt_kg.knowledge_graph", "module": "knowledge_graph", "funcName": "creates_new_edges", "lineno": 347, "message": "Created GENE-PROTEIN (entity-class) Edges: 174902 OWL Edges, 19521 Original Edges; 77505 OWL Nodes, Original Nodes: 19316 gene(s), 19134 protein(s)"}
{"asctime": "2021-11-02 05:16:43,553", "levelname": "INFO", "name": "pkt_kg.knowledge_graph", "module": "knowledge_graph", "funcName": "creates_new_edges", "lineno": 347, "message": "Created VARIANT-PHENOTYPE (entity-class) Edges: 22689 OWL Edges, 3005 Original Edges; 8555 OWL Nodes, Original Nodes: 2100 variant(s), 435 phenotype(s)"}
{"asctime": "2021-11-02 05:16:53,519", "levelname": "INFO", "name": "pkt_kg.knowledge_graph", "module": "knowledge_graph", "funcName": "creates_new_edges", "lineno": 347, "message": "Created GENE-GENE (entity-entity) Edges: 10819 OWL Edges, 1694 Original Edges; 3721 OWL Nodes, Original Nodes: 250 gene(s), 267 gene(s)"}
{"asctime": "2021-11-02 05:20:24,790", "levelname": "INFO", "name": "pkt_kg.knowledge_graph", "module": "knowledge_graph", "funcName": "creates_new_edges", "lineno": 347, "message": "Created CHEMICAL-PROTEIN (class-class) Edges: 442293 OWL Edges, 71679 Original Edges; 155580 OWL Nodes, Original Nodes: 4272 chemical(s), 7946 protein(s)"}
{"asctime": "2021-11-02 05:23:49,688", "levelname": "INFO", "name": "pkt_kg.knowledge_graph", "module": "knowledge_graph", "funcName": "creates_new_edges", "lineno": 347, "message": "Created RNA-PROTEIN (entity-class) Edges: 417047 OWL Edges, 44205 Original Edges; 151826 OWL Nodes, Original Nodes: 44202 rna(s), 19200 protein(s)"}
{"asctime": "2021-11-02 05:25:28,141", "levelname": "INFO", "name": "pkt_kg.knowledge_graph", "module": "knowledge_graph", "funcName": "creates_new_edges", "lineno": 347, "message": "Created GENE-PHENOTYPE (entity-class) Edges: 163019 OWL Edges, 24664 Original Edges; 57673 OWL Nodes, Original Nodes: 6782 gene(s), 1594 phenotype(s)"}
{"asctime": "2021-11-02 05:26:43,080", "levelname": "INFO", "name": "pkt_kg.knowledge_graph", "module": "knowledge_graph", "funcName": "creates_new_edges", "lineno": 347, "message": "Created CHEMICAL-GENE (class-entity) Edges: 124162 OWL Edges, 16652 Original Edges; 45687 OWL Nodes, Original Nodes: 466 chemical(s), 11923 gene(s)"}
{"asctime": "2021-11-02 05:26:47,258", "levelname": "INFO", "name": "pkt_kg.knowledge_graph", "module": "knowledge_graph", "funcName": "creates_new_edges", "lineno": 347, "message": "Created GOBP-PATHWAY (class-entity) Edges: 6745 OWL Edges, 671 Original Edges; 2578 OWL Nodes, Original Nodes: 478 gobp(s), 671 pathway(s)"}
{"asctime": "2021-11-02 05:36:55,564", "levelname": "INFO", "name": "pkt_kg.knowledge_graph", "module": "knowledge_graph", "funcName": "construct_knowledge_graph", "lineno": 590, "message": "See log: /PheKnowLator/resources/construction_approach/subclass_map_log.json"}
{"asctime": "2021-11-02 06:08:53,745", "levelname": "INFO", "name": "pkt_kg.knowledge_graph", "module": "knowledge_graph", "funcName": "construct_knowledge_graph", "lineno": 594, "message": "Full Logic Graph Stats: 25611453 triples, 8644230 nodes, 43 predicates, 4348593 classes, 190 individuals, 853 object props, 635 annotation props"}
{"asctime": "2021-11-02 07:23:38,482", "levelname": "INFO", "name": "pkt_kg.knowledge_graph", "module": "knowledge_graph", "funcName": "construct_knowledge_graph", "lineno": 596, "message": "Full Logic Subset (OWL) Graph Stats: 8644230 nodes, 25611453 edges, 2 self-loops, 5 most most common edges: http://www.w3.org/2000/01/rdf-schema#subClassOf:8411703, http://www.w3.org/1999/02/22-rdf-syntax-ns#type:8364393, http://www.w3.org/2002/07/owl#onProperty:3957847, http://www.w3.org/2002/07/owl#someValuesFrom:3953628, http://www.w3.org/1999/02/22-rdf-syntax-ns#first:280278, http://www.w3.org/1999/02/22-rdf-syntax-ns#rest:280278, average degree 2.962837985569565, 5 highest degree nodes: http://www.w3.org/2002/07/owl#Class:4348593, http://www.w3.org/2002/07/owl#Restriction:3957847,

http://purl.obolibrary.org/obo/RO_0002436:1001470, http://purl.obolibrary.org/obo/RO_0001025:689237, http://purl.obolibrary.org/obo/RO_0002200:428381, http://purl.obolibrary.org/obo/RO_0000056:381743, density: 3.427532965137278e-07, 2 component(s): {0: 8644227, 1: '3 nodes: http://www.w3.org/2002/07/owl#Ontology | http://purl.obolibrary.org/obo/chebi/204/chebi.owl | http://purl.obolibrary.org/obo/chebi.owl'}"}
{"asctime": "2021-11-02 07:31:29,029", "levelname": "INFO", "name": "pkt_kg.owlnets", "module": "owlnets", "funcName": "runs_owlnets", "lineno": 766, "message": "*** Running OWL-NETS ***"}
{"asctime": "2021-11-02 07:31:29,030", "levelname": "INFO", "name": "pkt_kg.owlnets", "module": "owlnets", "funcName": "removes_disjoint_with_axioms", "lineno": 130, "message": "Removing owl:disjointWith Axioms"}
{"asctime": "2021-11-02 07:31:29,156", "levelname": "INFO", "name": "pkt_kg.owlnets", "module": "owlnets", "funcName": "removes_edges_with_owl_semantics", "lineno": 157, "message": "Filtering Triples"}
{"asctime": "2021-11-02 07:39:27,341", "levelname": "INFO", "name": "pkt_kg.owlnets", "module": "owlnets", "funcName": "cleans_owl_encoded_entities", "lineno": 610, "message": "Decoding 114668 OWL Classes and Axioms"}
{"asctime": "2021-11-02 07:39:31,150", "levelname": "INFO", "name": "pkt_kg.owlnets", "module": "owlnets", "funcName": "cleans_owl_encoded_entities", "lineno": 610, "message": "Decoding 114667 OWL Classes and Axioms"}
{"asctime": "2021-11-02 07:39:35,410", "levelname": "INFO", "name": "pkt_kg.owlnets", "module": "owlnets", "funcName": "cleans_owl_encoded_entities", "lineno": 610, "message": "Decoding 114667 OWL Classes and Axioms"}
{"asctime": "2021-11-02 07:40:22,156", "levelname": "INFO", "name": "pkt_kg.owlnets", "module": "owlnets", "funcName": "cleans_owl_encoded_entities", "lineno": 610, "message": "Decoding 114667 OWL Classes and Axioms"}
{"asctime": "2021-11-02 07:44:34,169", "levelname": "INFO", "name": "pkt_kg.owlnets", "module": "owlnets", "funcName": "cleans_decoded_graph", "lineno": 206, "message": "Filtering Triples"}
{"asctime": "2021-11-02 07:44:34,653", "levelname": "INFO", "name": "pkt_kg.owlnets", "module": "owlnets", "funcName": "cleans_decoded_graph", "lineno": 206, "message": "Filtering Triples"}
{"asctime": "2021-11-02 07:45:14,083", "levelname": "INFO", "name": "pkt_kg.owlnets", "module": "owlnets", "funcName": "cleans_decoded_graph", "lineno": 206, "message": "Filtering Triples"}
{"asctime": "2021-11-02 07:46:20,730", "levelname": "INFO", "name": "pkt_kg.owlnets", "module": "owlnets", "funcName": "cleans_decoded_graph", "lineno": 206, "message": "Filtering Triples"}
{"asctime": "2021-11-02 07:47:22,051", "levelname": "INFO", "name": "pkt_kg.owlnets", "module": "owlnets", "funcName": "removes_disjoint_with_axioms", "lineno": 130, "message": "Removing owl:disjointWith Axioms"}
{"asctime": "2021-11-02 07:47:22,052", "levelname": "INFO", "name": "pkt_kg.owlnets", "module": "owlnets", "funcName": "removes_edges_with_owl_semantics", "lineno": 157, "message": "Filtering Triples"}
{"asctime": "2021-11-02 07:50:18,265", "levelname": "INFO", "name": "pkt_kg.owlnets", "module": "owlnets", "funcName": "cleans_owl_encoded_entities", "lineno": 610, "message": "Decoding 9364 OWL Classes and Axioms"}
{"asctime": "2021-11-02 07:50:18,715", "levelname": "INFO", "name": "pkt_kg.owlnets", "module": "owlnets", "funcName": "cleans_owl_encoded_entities", "lineno": 610, "message": "Decoding 9363 OWL Classes and Axioms"}
{"asctime": "2021-11-02 07:50:26,971", "levelname": "INFO", "name": "pkt_kg.owlnets", "module": "owlnets", "funcName": "cleans_owl_encoded_entities", "lineno": 610, "message": "Decoding 9363 OWL Classes and Axioms"}
{"asctime": "2021-11-02 07:51:00,114", "levelname": "INFO", "name": "pkt_kg.owlnets", "module": "owlnets", "funcName": "cleans_owl_encoded_entities", "lineno": 610, "message": "Decoding 9363 OWL Classes and Axioms"}
{"asctime": "2021-11-02 07:58:59,179", "levelname": "INFO", "name": "pkt_kg.owlnets", "module": "owlnets", "funcName": "cleans_decoded_graph", "lineno": 206, "message": "Filtering Triples"}
{"asctime": "2021-11-02 07:59:05,319", "levelname": "INFO", "name": "pkt_kg.owlnets", "module": "owlnets", "funcName": "cleans_decoded_graph", "lineno": 206, "message": "Filtering Triples"}
{"asctime": "2021-11-02 07:59:30,560", "levelname": "INFO", "name": "pkt_kg.owlnets", "module": "owlnets", "funcName": "cleans_decoded_graph", "lineno": 206, "message": "Filtering Triples"}
{"asctime": "2021-11-02 08:00:08,372", "levelname": "INFO", "name": "pkt_kg.owlnets", "module": "owlnets", "funcName": "cleans_decoded_graph", "lineno": 206, "message": "Filtering Triples"}
{"asctime": "2021-11-02 08:01:33,118", "levelname": "INFO", "name": "pkt_kg.owlnets", "module": "owlnets", "funcName": "removes_disjoint_with_axioms", "lineno": 130, "message": "Removing owl:disjointWith Axioms"}
{"asctime": "2021-11-02 08:01:33,119", "levelname": "INFO", "name": "pkt_kg.owlnets", "module": "owlnets", "funcName": "removes_edges_with_owl_semantics", "lineno": 157, "message": "Filtering Triples"}
{"asctime": "2021-11-02 08:06:55,548", "levelname": "INFO", "name": "pkt_kg.owlnets", "module": "owlnets", "funcName": "cleans_owl_encoded_entities", "lineno": 610, "message": "Decoding 65038 OWL Classes and Axioms"}
{"asctime": "2021-11-02 08:06:58,339", "levelname": "INFO", "name": "pkt_kg.owlnets", "module": "owlnets", "funcName": "cleans_owl_encoded_entities", "lineno": 610, "message": "Decoding 65038 OWL Classes and Axioms"}
{"asctime": "2021-11-02 08:07:01,427", "levelname": "INFO", "name": "pkt_kg.owlnets", "module": "owlnets", "funcName": "cleans_owl_encoded_entities", "lineno": 610, "message": "Decoding 65038 OWL Classes and Axioms"}
{"asctime": "2021-11-02 08:07:43,352", "levelname": "INFO", "name": "pkt_kg.owlnets", "module": "owlnets", "funcName": "cleans_owl_encoded_entities", "lineno": 610, "message": "Decoding 65038 OWL Classes and Axioms"}
{"asctime": "2021-11-02 08:12:06,872", "levelname": "INFO", "name": "pkt_kg.owlnets", "module": "owlnets", "funcName": "cleans_decoded_graph", "lineno": 206, "message": "Filtering Triples"}
{"asctime": "2021-11-02 08:15:37,045", "levelname": "INFO", "name": "pkt_kg.owlnets", "module": "owlnets", "funcName": "cleans_decoded_graph", "lineno": 206, "message": "Filtering Triples"}
{"asctime": "2021-11-02 08:17:56,512", "levelname": "INFO", "name": "pkt_kg.owlnets", "module": "owlnets", "funcName": "cleans_decoded_graph", "lineno": 206, "message": "Filtering Triples"}
{"asctime": "2021-11-02 08:20:01,999", "levelname": "INFO", "name": "pkt_kg.owlnets", "module": "owlnets", "funcName": "cleans_decoded_graph", "lineno": 206, "message": "Filtering Triples"}
{"asctime": "2021-11-02 08:21:33,746", "levelname": "INFO", "name": "pkt_kg.owlnets", "module": "owlnets", "funcName": "removes_disjoint_with_axioms", "lineno": 130, "message": "Removing owl:disjointWith Axioms"}
{"asctime": "2021-11-02 08:21:33,747", "levelname": "INFO", "name": "pkt_kg.owlnets", "module": "owlnets", "funcName": "removes_edges_with_owl_semantics", "lineno": 157, "message": "Filtering Triples"}
{"asctime": "2021-11-02 08:24:47,308", "levelname": "INFO", "name": "pkt_kg.owlnets", "module": "owlnets", "funcName": "cleans_owl_encoded_entities", "lineno": 610, "message": "Decoding 18729 OWL Classes and Axioms"}
{"asctime": "2021-11-02 08:24:47,628", "levelname": "INFO", "name": "pkt_kg.owlnets", "module": "owlnets", "funcName": "cleans_owl_encoded_entities", "lineno": 610, "message": "Decoding 18729 OWL Classes and Axioms"}
{"asctime": "2021-11-02 08:24:52,252", "levelname": "INFO", "name": "pkt_kg.owlnets", "module": "owlnets", "funcName": "cleans_owl_encoded_entities", "lineno": 610, "message": "Decoding 18729 OWL Classes and Axioms"}
{"asctime": "2021-11-02 08:25:26,246", "levelname": "INFO", "name": "pkt_kg.owlnets", "module": "owlnets", "funcName": "cleans_owl_encoded_entities", "lineno": 610, "message": "Decoding 18729 OWL Classes and Axioms"}
{"asctime": "2021-11-02 08:32:14,445", "levelname": "INFO", "name": "pkt_kg.owlnets", "module": "owlnets", "funcName": "cleans_decoded_graph", "lineno": 206, "message": "Filtering Triples"}
{"asctime": "2021-11-02 08:32:54,439", "levelname": "INFO", "name": "pkt_kg.owlnets", "module": "owlnets", "funcName": "cleans_decoded_graph", "lineno": 206, "message": "Filtering Triples"}
{"asctime": "2021-11-02 08:33:02,489", "levelname": "INFO", "name": "pkt_kg.owlnets", "module": "owlnets", "funcName": "cleans_decoded_graph", "lineno": 206, "message": "Filtering Triples"}
{"asctime": "2021-11-02 08:33:26,285", "levelname": "INFO", "name": "pkt_kg.owlnets", "module": "owlnets", "funcName": "cleans_decoded_graph", "lineno": 206, "message": "Filtering Triples"}
{"asctime": "2021-11-02 08:34:53,681", "levelname": "INFO", "name": "pkt_kg.owlnets", "module": "owlnets", "funcName": "removes_disjoint_with_axioms", "lineno": 130, "message": "Removing owl:disjointWith Axioms"}
{"asctime": "2021-11-02 08:34:53,682", "levelname": "INFO", "name": "pkt_kg.owlnets", "module": "owlnets", "funcName": "removes_edges_with_owl_semantics", "lineno": 157, "message": "Filtering Triples"}
{"asctime": "2021-11-02 08:40:42,490", "levelname": "INFO", "name": "pkt_kg.owlnets", "module": "owlnets", "funcName": "cleans_owl_encoded_entities", "lineno": 610, "message": "Decoding 63702 OWL Classes and Axioms"}
{"asctime": "2021-11-02 08:40:45,645", "levelname": "INFO", "name": "pkt_kg.owlnets", "module": "owlnets", "funcName": "cleans_owl_encoded_entities", "lineno": 610, "message": "Decoding 63702 OWL Classes and Axioms"}
{"asctime": "2021-11-02 08:40:48,594", "levelname": "INFO", "name": "pkt_kg.owlnets", "module": "owlnets", "funcName": "cleans_owl_encoded_entities", "lineno": 610, "message": "Decoding 63701 OWL Classes and Axioms"}
{"asctime": "2021-11-02 08:41:31,818", "levelname": "INFO", "name": "pkt_kg.owlnets", "module": "owlnets", "funcName": "cleans_owl_encoded_entities", "lineno": 610, "message": "Decoding 63701 OWL Classes and Axioms"}
{"asctime": "2021-11-02 08:54:09,937", "levelname": "INFO", "name": "pkt_kg.owlnets", "module": "owlnets", "funcName": "cleans_decoded_graph", "lineno": 206, "message": "Filtering Triples"}
{"asctime": "2021-11-02 08:54:34,397", "levelname": "INFO", "name": "pkt_kg.owlnets", "module": "owlnets", "funcName": "cleans_decoded_graph", "lineno": 206, "message": "Filtering Triples"}
{"asctime": "2021-11-02 08:54:52,950", "levelname": "INFO", "name": "pkt_kg.owlnets", "module": "owlnets", "funcName": "cleans_decoded_graph", "lineno": 206, "message": "Filtering Triples"}
{"asctime": "2021-11-02 08:55:50,180", "levelname": "INFO", "name": "pkt_kg.owlnets", "module": "owlnets", "funcName": "cleans_decoded_graph", "lineno": 206, "message": "Filtering Triples"}
{"asctime": "2021-11-02 08:57:26,093", "levelname": "INFO", "name": "pkt_kg.owlnets", "module": "owlnets", "funcName": "makes_graph_connected", "lineno": 663, "message": "Ensuring OWL-NETS Graph Contains a Single Connected Component"}
{"asctime": "2021-11-02 08:57:26,093", "levelname": "INFO", "name": "pkt_kg.owlnets", "module": "owlnets", "funcName": "makes_graph_connected", "lineno": 667, "message": "Obtaining node list"}
{"asctime": "2021-11-02 09:19:25,234", "levelname": "INFO", "name": "pkt_kg.owlnets", "module": "owlnets", "funcName": "makes_graph_connected", "lineno": 683, "message": "Updating graph connectivity"}
{"asctime": "2021-11-02 09:19:25,287", "levelname": "INFO", "name": "pkt_kg.owlnets", "module": "owlnets", "funcName": "makes_graph_connected", "lineno": 688, "message": "1100 triples added to make connected"}
{"asctime": "2021-11-02 09:23:55,212", "levelname": "INFO", "name": "pkt_kg.owlnets", "module": "owlnets", "funcName": "write_out_results", "lineno": 737, "message": "Serializing OWL-NETS Graph"}
{"asctime": "2021-11-02 09:39:00,282", "levelname": "INFO", "name": "pkt_kg.owlnets", "module": "owlnets", "funcName": "write_out_results", "lineno": 751, "message": "OWL-NETS Graph Stats: 780753 nodes, 5072062 edges, 441 self-loops, 5 most most common edges: http://www.w3.org/2000/01/rdf-schema#subClassOf:1204474, http://purl.obolibrary.org/obo/RO_0002436:1001464, http://purl.obolibrary.org/obo/RO_0001025:689128, http://purl.obolibrary.org/obo/RO_0002200:428374, http://purl.obolibrary.org/obo/RO_0000056:381721, http://purl.obolibrary.org/obo/RO_0002606:279052, average degree 6.496372092070091, 5 highest degree nodes: http://purl.obolibrary.org/obo/SO_0000673:190850, http://purl.obolibrary.org/obo/SO_0001483:130579, http://purl.obolibrary.org/obo/NCBITaxon_9606:117091, http://purl.obolibrary.org/obo/SO_0001217:105056, http://purl.obolibrary.org/obo/SO_0002113:29340, http://purl.obolibrary.org/obo/SO_0001503:27620, density: 8.320660199487278e-06, 1 component(s): {0: 780753}"}

{"asctime": "2021-11-02 09:39:00,284", "levelname": "INFO", "name": "pkt_kg.owlnets", "module": "owlnets", "funcName": "purifies_graph_build", "lineno": 703, "message": "Purifying Graph Based on Construction Approach"}
{"asctime": "2021-11-02 09:39:00,284", "levelname": "INFO", "name": "pkt_kg.owlnets", "module": "owlnets", "funcName": "purifies_graph_build", "lineno": 708, "message": "Determining what triples need purification"}
{"asctime": "2021-11-02 09:39:00,287", "levelname": "INFO", "name": "pkt_kg.owlnets", "module": "owlnets", "funcName": "purifies_graph_build", "lineno": 711, "message": "Processing 161 http://www.w3.org/1999/02/22-rdf-syntax-ns#type triples"}
{"asctime": "2021-11-02 09:43:24,714", "levelname": "INFO", "name": "pkt_kg.owlnets", "module": "owlnets", "funcName": "write_out_results", "lineno": 737, "message": "Serializing Subclass-Purified OWL-NETS Graph"}
{"asctime": "2021-11-02 09:58:01,555", "levelname": "INFO", "name": "pkt_kg.owlnets", "module": "owlnets", "funcName": "write_out_results", "lineno": 751, "message": "Subclass-Purified OWL-NETS Graph Stats: 780753 nodes, 5072064 edges, 441 self-loops, 5 most most common edges: http://www.w3.org/2000/01/rdf-schema#subClassOf:1204637, http://purl.obolibrary.org/obo/RO_0002436:1001464, http://purl.obolibrary.org/obo/RO_0001025:689128, http://purl.obolibrary.org/obo/RO_0002200:428374, http://purl.obolibrary.org/obo/RO_0000056:381721, http://purl.obolibrary.org/obo/RO_0002606:279052, average degree 6.496374653699697, 5 highest degree nodes: http://purl.obolibrary.org/obo/SO_0000673:190850, http://purl.obolibrary.org/obo/SO_0001483:130579, http://purl.obolibrary.org/obo/NCBITaxon_9606:117091, http://purl.obolibrary.org/obo/SO_0001217:105056, http://purl.obolibrary.org/obo/SO_0002113:29340, http://purl.obolibrary.org/obo/SO_0001503:27620, density: 8.3206634804646e-06, 1 component(s): {0: 780753}"}
{"asctime": "2021-11-02 09:58:01,557", "levelname": "INFO", "name": "pkt_kg.owlnets", "module": "owlnets", "funcName": "runs_owlnets", "lineno": 796, "message": "\n\nOWL-NETS Graph Stats: 5072062 triples, 780753 nodes, 290 predicates, 0 classes, 0 individuals, 0 object props, 0 annotation props;\nPurified OWL-NETS Graph Stats: 5072064 triples, 780753 nodes, 289 predicates, 0 classes, 0 individuals, 0 object props, 0 annotation props"}
{"asctime": "2021-11-02 09:58:06,686", "levelname": "INFO", "name": "pkt_kg.owlnets", "module": "owlnets", "funcName": "runs_owlnets", "lineno": 812, "message": "Decoded 474111 owl-encoded classes and axioms. Note the following:\nPartially processed 364 cardinality elements\nRemoved 1645 owl:disjointWith axioms\nIgnored: 3 misc classes; 19 classes constructed with owl:complementOf; 4813 classes containing negation (e.g. pr#lacks_part, cl#has_not_completed)\nFiltering removed 17563627 semantic support triples"}
{"asctime": "2021-11-02 09:58:07,196", "levelname": "INFO", "name": "pkt_kg.knowledge_graph", "module": "knowledge_graph", "funcName": "construct_knowledge_graph", "lineno": 604, "message": "*** Writing Knowledge Graph Edge Lists ***"}
{"asctime": "2021-11-02 09:58:07,197", "levelname": "INFO", "name": "pkt_kg.knowledge_graph", "module": "knowledge_graph", "funcName": "construct_knowledge_graph", "lineno": 609, "message": "*** Processing OWL Graph ***"}
{"asctime": "2021-11-02 10:06:19,866", "levelname": "INFO", "name": "pkt_kg.metadata", "module": "metadata", "funcName": "output_metadata", "lineno": 258, "message": "Writing Class Metadata"}
{"asctime": "2021-11-02 10:13:05,566", "levelname": "INFO", "name": "pkt_kg.knowledge_graph", "module": "knowledge_graph", "funcName": "construct_knowledge_graph", "lineno": 609, "message": "*** Processing OWL-NETS Graph ***"}
{"asctime": "2021-11-02 10:14:37,082", "levelname": "INFO", "name": "pkt_kg.metadata", "module": "metadata", "funcName": "output_metadata", "lineno": 258, "message": "Writing Class Metadata"}
{"asctime": "2021-11-02 10:15:59,257", "levelname": "INFO", "name": "pkt_kg.knowledge_graph", "module": "knowledge_graph", "funcName": "construct_knowledge_graph", "lineno": 609, "message": "*** Processing Purified OWL-NETS Graph ***"}
{"asctime": "2021-11-02 10:17:29,232", "levelname": "INFO", "name": "pkt_kg.metadata", "module": "metadata", "funcName": "output_metadata", "lineno": 258, "message": "Writing Class Metadata"}
{"asctime": "2021-11-02 10:23:46,066", "levelname": "INFO", "name": "pkt_kg.knowledge_graph", "module": "knowledge_graph", "funcName": "construct_knowledge_graph", "lineno": 620, "message": "\nLoading Full (Logic + Annotation) Graph"}
{"asctime": "2021-11-02 11:44:38,694", "levelname": "INFO", "name": "pkt_kg.knowledge_graph", "module": "knowledge_graph", "funcName": "construct_knowledge_graph", "lineno": 621, "message": "Deriving Stats"}
{"asctime": "2021-11-02 12:05:30,840", "levelname": "INFO", "name": "pkt_kg.knowledge_graph", "module": "knowledge_graph", "funcName": "construct_knowledge_graph", "lineno": 622, "message": "Full (Logic + Annotation) Graph Stats: 36201468 triples, 13680807 nodes, 355 predicates, 4348593 classes, 190 individuals, 853 object props, 635 annotation props"}
{"asctime": "2021-11-02 12:10:31,817", "levelname": "INFO", "name": "__main__", "module": "build_phase_3", "funcName": "main", "lineno": 125, "message": "STEP 3: UPLOAD KNOWLEDGE GRAPH DATA TO GOOGLE CLOUD STORAGE"}
{"asctime": "2021-11-02 12:10:31,818", "levelname": "INFO", "name": "__main__", "module": "build_phase_3", "funcName": "main", "lineno": 128, "message": "Removing Existing Data from Current Build Directory on Google Cloud Storage"}
{"asctime": "2021-11-02 12:10:48,261", "levelname": "INFO", "name": "__main__", "module": "build_phase_3", "funcName": "main", "lineno": 138, "message": "Copying Data FROM: archived_builds/release_v3.0.2/build_01NOV2021/data/ TO: current_build/data/"}
{"asctime": "2021-11-02 12:10:58,297", "levelname": "INFO", "name": "__main__", "module": "build_phase_3", "funcName": "main", "lineno": 143, "message": "Uploading Knowledge Graph Data from Docker to the archived_builds Directory"}
{"asctime": "2021-11-02 12:16:11,232", "levelname": "INFO", "name": "__main__", "module": "build_phase_3", "funcName": "main", "lineno": 148, "message": "Copying Graph Data from archived_builds to current_builds"}
{"asctime": "2021-11-02 12:16:16,599", "levelname": "INFO", "name": "__main__", "module": "build_phase_3", "funcName": "main", "lineno": 160, "message": "STEP 4: BUILD CLEAN-UP"}
{"asctime": "2021-11-02 12:16:16,599", "levelname": "INFO", "name": "__main__", "module": "build_phase_3", "funcName": "main", "lineno": 163, "message": "COMPLETED BUILD PHASE 3: 674.132 MINUTES"}
{"asctime": "2021-11-02 12:16:16,599", "levelname": "INFO", "name": "__main__", "module": "build_phase_3", "funcName": "main", "lineno": 163, "message": "EXIT BUILD PHASE 3"}
